# The dominant *Anopheles *vectors of human malaria in the Asia-Pacific region: occurrence data, distribution maps and bionomic précis

**DOI:** 10.1186/1756-3305-4-89

**Published:** 2011-05-25

**Authors:** Marianne E Sinka, Michael J Bangs, Sylvie Manguin, Theeraphap Chareonviriyaphap, Anand P Patil, William H Temperley, Peter W Gething, Iqbal RF Elyazar, Caroline W Kabaria, Ralph E Harbach, Simon I Hay

**Affiliations:** 1 Spatial Ecology and Epidemiology Group, Tinbergen Building, Department of Zoology, University of Oxford, South Parks Road, Oxford OX1 3PS, UK; 2 Public Health and Malaria Control Department, PT Freeport Indonesia, Kuala Kencana, Papua, Indonesia; 3 Institut de Recherche pour le Développement, Lab. d'Immuno-Physiopathologie Moléculaire Comparée, UMR-MD3/Univ. Montpellier 1, Faculté de Pharmacie, 15, Ave Charles Flahault, 34093 Montpellier, France; 4 Department of Entomology, Faculty of Agriculture, Kasetsart University, Bangkok, Thailand; 5 Eijkman-Oxford Clinical Research Unit, Jakarta, Indonesia; 6 Malaria Public Health and Epidemiology Group, Centre for Geographic Medicine, KEMRI - Univ. Oxford - Wellcome Trust Collaborative Programme, Kenyatta National Hospital Grounds, P.O. Box 43640-00100 Nairobi, Kenya; 7 Department of Entomology, The Natural History Museum, Cromwell Road, London, SW7 5BD, UK

## Abstract

**Background:**

The final article in a series of three publications examining the global distribution of 41 dominant vector species (DVS) of malaria is presented here. The first publication examined the DVS from the Americas, with the second covering those species present in Africa, Europe and the Middle East. Here we discuss the 19 DVS of the Asian-Pacific region. This region experiences a high diversity of vector species, many occurring sympatrically, which, combined with the occurrence of a high number of species complexes and suspected species complexes, and behavioural plasticity of many of these major vectors, adds a level of entomological complexity not comparable elsewhere globally. To try and untangle the intricacy of the vectors of this region and to increase the effectiveness of vector control interventions, an understanding of the contemporary distribution of each species, combined with a synthesis of the current knowledge of their behaviour and ecology is needed.

**Results:**

Expert opinion (EO) range maps, created with the most up-to-date expert knowledge of each DVS distribution, were combined with a contemporary database of occurrence data and a suite of open access, environmental and climatic variables. Using the Boosted Regression Tree (BRT) modelling method, distribution maps of each DVS were produced. The occurrence data were abstracted from the formal, published literature, plus other relevant sources, resulting in the collation of DVS occurrence at 10116 locations across 31 countries, of which 8853 were successfully geo-referenced and 7430 were resolved to spatial areas that could be included in the BRT model. A detailed summary of the information on the bionomics of each species and species complex is also presented.

**Conclusions:**

This article concludes a project aimed to establish the contemporary global distribution of the DVS of malaria. The three articles produced are intended as a detailed reference for scientists continuing research into the aspects of taxonomy, biology and ecology relevant to species-specific vector control. This research is particularly relevant to help unravel the complicated taxonomic status, ecology and epidemiology of the vectors of the Asia-Pacific region. All the occurrence data, predictive maps and EO-shape files generated during the production of these publications will be made available in the public domain. We hope that this will encourage data sharing to improve future iterations of the distribution maps.

## Background

The Asian-Pacific region experiences a notably high diversity of vector species, species complexes and suspected species complexes, many occurring sympatrically and exhibiting a high level of behavioural plasticity [1]. This complexity, and the taxonomic ambiguity of many of the dominant vector species (DVS) of the region, is a major contributing factor to the continuing impact of malaria in this area.

Second only to Africa, central and southeastern Asia suffers with 39% of the global malaria burden (estimated clinical cases of *P. falciparum *malaria only) [2], with pockets of medium to high endemicity found in Orissa State (eastern India), western Myanmar and the lowlands of New Guinea [3]. The impact of *Plasmodium vivax *is also considerable, with an estimated 41.9% of the global population at risk (PAR) of *P. vivax *transmission occurring in India alone. Seven other Asian nations (China, Indonesia, Pakistan, Vietnam, the Philippines, Myanmar and Thailand) are also listed within the top 10 countries with the highest *P. vivax *PAR estimates [4].

Of 41 DVS recognised globally [5], 19 occur in the Asian-Pacific region and of these, at least ten are now considered as species complexes [6] (Table 1). A species complex tends to encompass a group of closely related, morphologically indistinguishable species, which may occur in sympatry (but not interbreeding), yet still display behavioural differences that could confound any control efforts that ignore their bionomics and epidemiological importance [7,8]. Moreover, even amongst those species that are not members of a complex, behavioural differences are common depending upon location, such that a species can be considered a primary vector in one area, but of secondary or no importance elsewhere [8].

**Table 1:** Defining the dominant *Anopheles *vector species and species complexes of human malaria in the Asia-Pacific region.

Anopheline species or species complex	White [32]	Service [522,523]	Kiszewski [524]	Mouchet [525]	Malaria epidemiology zone (Bangs, unpub obs)	EO source
*An. aconitus*	y	y		y	8, 9, 10	[32] + TAG
*An. annularis*			y		8, 9, 10	TAG
*An. balabacensis*	y	y		y	10	[28,32] + TAG
An. barbirostris*			y		8, 9, 10	[29,32] + TAG
An. culicifacies*	y	y	y	y	6(?), 7, 8, 9, 10(?)	[8,32] + TAG
An. dirus*	y	y	y	y	9, 10	[8,29,32] + TAG
An. farauti*	y	y		y	12	[32] + TAG
*An. flavirostris*		y	y	y	10	[32] + TAG
An. fluviatilis*	y	y	y	y	8, 9	[8,32] + TAG
*An. koliensis*		y		y	12	[32] + TAG
*An. lesteri*		y	y	y	9(?), 10	Harbach (unpub obs) + TAG
*An. leucosphyrus & An. latens*	y	y			10	[8,28,32] + TAG
*An. maculatus *group	y	y	y	y	8, 9, 10	[8,32] + TAG
An. minimus*	y	y	y	y	8, 9, 10	[8,32] + TAG
An. punctulatus*		y	y	y	12	[32] + TAG
An. sinensis*	y	y	y	y	4(?), 8(?), 9, 10, 11	[32] + TAG
*An. stephensi*			y	y	6(?), 8, 9	[32] + TAG
An. subpictus*	y	y			8, 9, 10, 12	TAG
An. sundaicus*	y	y	y	y	9(?), 10	[8,25] + TAG

An asterisk (*) denotes that a "species" is now recognised as a species complex. Macdonald's malaria epidemiology zones [521] 6, Desert; 7, Ethiopian; 8, Indo-Persian; 9, Indo-Chinese Hills; 10, Malaysian; 11, Chinese 12, Australasian. A question mark (?) indicates uncertainty of presence in the listed zone. The final DVS species listed were defined during two separate Technical Advisory Group (TAG) meetings.

The correct identification of any vector implicated in malaria transmission is key to successful control. For example, in central Vietnam, where members of both the *An. dirus *and *An. minimus *species complexes were considered primary vectors, control was targeted twice a year to precede the period of malaria transmission attributed to each species complex. However, Van Bortel *et al*. [9], discovered that the mosquitoes previously identified as *An. minimus *were actually *An. varuna *(a member of the Funestus Group that also includes the Minimus Complex). *Anopheles varuna *is highly zoophilic in the study area and therefore a non-vector. This misidentification resulted in the misdirection of valuable and limited resources and highlights common difficulties in vector control in the Asian-Pacific region where the close relationship and sympatric distributions of many of the species can result in the application of unnecessary or unsuitable intervention methods. Moreover, in an increasingly changing environment, deforestation, the implementation of new irrigation programmes and expanding agricultural development can rapidly alter the composition of the local mosquito fauna [10-14], and subsequently influence the control methods required.

Nonetheless, vector control has proved highly successful in areas of the Asian-Pacific region with the WHO reporting a greater than 50% decrease in the number of malaria cases in Bhutan, the Democratic People's Republic of Korea, Sri Lanka and Thailand since 2000 [15]. This reduction is associated with intensive malaria intervention programmes, including indoor residual spraying (IRS) of insecticides and the distribution of insecticide-treated bednets (ITNs). Successful control has also been implemented to take advantage of species-specific behaviour, for example the introduction of small larvivorous fish into the intra-domestic water containers that served as larval habitats for the 'urban vector' *An. stephensi *[16].

The DVS of the Asian-Pacific region have been the subject of a number of comprehensive reviews (e.g. [7,8,17-26]) and attempts to establish their distribution ranging from simple maps identifying sampling locations (e.g. [18,27-31]), expert opinion maps (e.g. [8,25,32,33]), GIS overlays (e.g. [18,34-38]) and those employing methods to predict species distributions (e.g. [39-42]). Surprisingly few attempts have been made with the application of more sophisticated modelling methods to the DVS of Asia, essentially only those of Foley *et al*. [39,41,42].

This current work presents predicted distribution maps for the 19 DVS of the Asian-Pacific region, created using the Boosted Regression Tree (BRT) methodology and applied to a comprehensive database of contemporary (post 31 December 1984) occurrence data. The modelling method also benefits from the inclusion of updated expert opinion (EO) ranges for each species or species complex, specifically useful for those species with limited occurrence records. The predictive maps are presented alongside a bionomics summary of each species/species complex, highlighting the complexity of many of the species that occur in this region.

## Methods

An introduction to the MAP DVS project, including details on DVS selection, is given in Hay *et al*. [5]. A full description of the DVS bionomics and occurrence data assembly, modelling and mapping protocols, and climatic and environmental variable grid pre- and post-processing, is provided in Sinka *et al*. [43].

From a list of 41 DVS recognised globally, 19 species or species complexes are found within the Asian-Pacific region (Table 1). These species range from the Arabian Peninsula (e.g. *An. stephensi *and the *An. culicifacies *complex) across the Indian subcontinent, north into China and Korea (e.g. *An. lesteri*) and as far west as the Solomon Islands and Vanuatu (e.g. the *An. farauti *complex) and south into Queensland and the Northern Territory of Australia (e.g. the *An. farauti *complex).

### Data assembly, data checks and expert opinion maps

A systematic search of the published, peer-reviewed scientific and medical literature, using online bibliographic databases [44,45], was conducted and augmented with a range of focussed searches of other relevant data sources [46-49]. Searches were concluded on 31 October 2009, and all literature containing data meeting our search criteria [43] were reviewed. Following the protocol described in Hay *et al*. [5], data were extracted and processed through a series of rigorous checking procedures before migration into a web-based PostgreSQL database, where a final series of checks were conducted [43].

A total of 3857 publications and reports were amalgamated for review. Of these, 2276 fulfilled the inclusion criteria [5,43], culminating in the assembly of DVS occurrence data for 147 countries. The 19 Asian-Pacific species and species complexes were distributed across 31 countries from data abstracted from 875 sources.

Preliminary maps were produced by overlaying occurrence data points over expert opinion (EO, see Table 1) species distribution ranges (Additional file 1: Expert opinion distribution maps for the 19 DVS of the Asian-Pacific region (Raster prediction files are available on request)). These maps were examined and refined by a technical advisory group (TAG, see acknowledgments) of *Anopheles *experts, and any data points that had fallen outside of the known EO ranges were checked and, where necessary, the EO ranges were adjusted to incorporate any confirmed areas of species presence.

### Boosted Regression Trees, climatic/environmental variables and model protocol

The Boosted Regression Tree method uses open access, reliable and well-supported R code and benefits from a flexibility that allows it to utilise both categorical and continuous data [50-52]. The maps produced are easy to interpret and are accompanied by a clear ranking of each environmental or climatic predictor variable identified by the model as relevant to the distribution of the species being mapped (see below). Moreover, in a review that rigorously tested 16 species modelling methods, BRTs were shown to consistently perform well [53]. The BRT method is described in detail in Elith *et al*. [50] and summarised in Sinka *et al*. [43] with specific reference to its implementation in DVS mapping. The evaluation statistics produced using this method (Deviance, Correlation, Discrimination (Area Under the operating characteristic Curve: AUC) and Kappa (κ)) were used to guide the assessment of the predictive performance of the models.

A suite of open access, environmental and climatic variable 5 × 5 km resolution grids, relevant to the ecology and biology of the DVS, were assembled. These included a digital elevation model (DEM) [54-56], precipitation [57,58], land surface temperature (LST), middle infra-red radiation (MIR) and the Normalized Difference Vegetation Index (NDVI) (Advanced Very High Resolution Radiometer (AVHRR) [59-62]) plus 22 individual categories of land cover including three grouped classes: flooded areas, forested areas and dry areas (Globcover [63] (A full list of the variables applied is given in Additional file 2: Summary tables showing evaluation statistics for all mapping trials and final BRT environmental and climatic variable selections for the final, optimal predictive maps). Each grid underwent a series of processing steps to ensure all land and sea pixels exactly corresponded. Nearest-neighbour interpolation was used to fill in any small gaps in the data, for example, those areas obscured by cloud cover. Temporal Fourier analysis was applied to all multi-temporal data to generate seven products: the overall mean, maximum and minimum of the data cycles, the amplitude (maximum variation of the cycle around the mean) and the phase (timing of the cycle) of the annual and bi-annual cycles [64].

A detailed protocol describing the modelling procedures followed to attain the 'optimal' mapping outputs are given in Sinka *et al*. [43]. Briefly, these included running a series of model iterations to assess the effects of incorporating half-weighted pseudo-presence data (randomly allocated from within the known EO range of each DVS), the number of pseudo-absences required and the extent of buffer from which they should be drawn. The buffer was established by extending the range limit of the EO maps to provide an area of terrain next to the known range of each DVS, and therefore close to the feasible range of conditions for the species to exist. Pseudo-absences were assigned within the buffer area at random. The evaluation metrics provided by the BRT could only be used as a guide to the predictive performance of each series of maps, as each iteration was created using different data inputs. Thus the 'optimal' settings chosen should be considered as subjective, as they were based on a combination of visual assessment guided by, but not relying on, the evaluation metrics.

### Bionomics

A bionomics summary of each of the Asian-Pacific DVS is provided to accompany the predictive maps. The large geographic ranges, presence of a relatively high proportion of identified and suspected species complexes and a level of behavioural plasticity amongst many of the DVS across this region, adds a level of complexity to summarising each of the species' behaviour. However, understanding their bionomics is crucial to the success of any interventions applied to control these malaria vectors. The review provided here does address some of the complexities, but is a general summary provided with the caveat that local expertise should always be additionally consulted when evaluating the possible vector control methods to be applied in specific locations.

The bionomics summaries do not include a detailed assessment of insecticide resistance amongst the DVS of the Asian-Pacific region. Whilst resistance appears widespread in many of the species, and is therefore an important aspect that must be considered before the application of any chemical-based control intervention, it was not possible to do full justice to this area of vector biology within the confines of this current work. However, insecticide resistance is being addressed by a number of other research groups and projects (e.g. MALVECASIA [65,66]) and a number of recent publications (e.g. [23,67-77]) provide detailed information that should be considered alongside this current work.

The full protocol applied to extracting bionomics data from the available literature (Table 2) is provided in the supplemental information accompanying Sinka *et al*. [43]. Due to the large number of studies available for some of the DVS in this region, an additional filtering step was necessary to maintain a reasonable sized data source for summary. Where the number of citations remained significantly high (> 100) after following the steps outlined in Sinka *et al*. [43], which included filtering the literature using the terms 'behaviour', 'larva', 'biting' etc., the remaining citations were manually searched to provide a minimum of 30 articles, ensuring the most recent studies that examined all the relevant bionomics were included in the summary.

**Table 2:** Search results for bionomics survey for the 19 Asian and Pacific DVS.

Species	References
*An. aconitus*	[10,100,154,156-158,161,163,164,174-177,179,181,182,186,187,189-191]

*An. annularis*	[10,12,13,92,154,156-158,161,163-165,174,179,182,186,187,189-191,215,216,230,262,301,304,478,479,481,482,487,490,526-528]

*An. balabacensis*	[11,221,222,227,230,232,237,308,529]

An. barbirostris*	[10,12,92,100,154,156-158,161,163,164,174,177,179,182,186,187,189-191,215,247,262,349,472,479,481,482,487,528-531]

An. culicifacies*	[10,12,13,156-158,161,163,164,186,190,191,215,216,252,262,322,472,479,481,482,487,490,526,527,530-542]

An. dirus*	[9,35,155,175,181,182,189,215,275,278,280-282,284,285,289-294,348,349,351,533,543-553]

An. farauti*	[30,31,371,381,389-391,397,408,409,411,413,420,554-563]

*An. flavirostris*	[174,187,227,230,296,301,303-305,307,308]

An. fluviatilis*	[156-158,161,163,191,203,216,283,294,316,319-324,490,537,564-566]

*An. koliensis*	[31,383,386,391,402,406,408,412,425,561,567,568]

*An. lesteri*	[36,121,467,485,569-573]

*An. leucosphyrus & An. latens*	[221,329]

*An. maculatus *group	[40,92,100,155,156,158,174-177,181,182,187,189,215,247,281,283,284,304,305,319,343,348,349,472,546,553,574-576]

An. minimus*	[27,35,40,143,150,155,181,182,189,215,247,281,283,294,348-351,360,364,369,528,533,545-548,577-580]

An. punctulatus*	[31,371,381,386,389-391,401,403,408,411-413,425,581,582]

An. sinensis*	[14,36,100,121,128,138,139,141-143,177,181,182,189,467,485,569,573,583-587]

*An. stephensi*	[16,161,163,190,191,322,428,438,440-443,464,476,479,487,489,490,527,542,588-597]

An. subpictus*	[10,12,13,16,154,156-158,161,163-165,179,186,190,191,230,252,262,452,458,467,470,472,474,476,478-490,527,598]

An. sundaicus*	[181,187,461,486,497,505,518,529,599-603]

Libraries created from MAP database. Filter terms were: 'behaviour', 'behavior', 'larva', 'biting', 'resting' and 'habitat'. An asterisk (*) denotes that a "species" is now recognised as a species complex.

## Results

The presence of one or more Asian-Pacific DVS was reported by 875 sources from 10116 independent sites, of which 8853 were successfully geo-referenced. These data related to 19110 occurrences (i.e. a study that sampled at one site on one occasion results in one occurrence and one site, a study that samples every month for a year at the same site results in one site but 12 occurrences) of which 15410 were from geo-referenced locations (Table 3).

**Table 3:** Geo-referenced independent site and occurrence (includes multiple sampling at a single site) data for the 19 Asian-Pacific species by country.

	Site			Occurrence		
	
Country	All	Data	Polygons	All	Data	Polygons
Afghanistan	14	1	13	55	1	54

Australia	505	493	12	658	645	13

Bangladesh	35	14	21	39	18	21

Cambodia	43	37	6	46	40	6

China	355	160	195	665	328	337

Eritrea	1	1	0	1	1	0

India	1529	673	856	3935	1933	2002

Indonesia	890	865	25	931	906	25

Iran	59	49	10	161	120	41

Iraq	4	1	3	4	1	3

Japan	7	6	1	10	9	1

Korea, Democratic People's Republic of	30	30	0	44	44	0

Korea, Republic of	242	234	8	319	267	52

Lao People's Democratic Republic	139	121	18	210	192	18

Malaysia	145	132	13	317	297	20

Myanmar	1830	1791	39	2777	2724	53

Nepal	33	33	0	263	263	0

Pakistan	54	41	13	209	144	65

Papua New Guinea	1503	1487	16	1742	1725	17

Philippines	124	113	11	188	147	41

Singapore	1	1	0	1	1	0

Solomon Islands	160	157	3	291	270	21

Sri Lanka	303	273	30	1229	1121	108

Taiwan Province of China	14	6	8	15	7	8

Thailand	505	412	93	908	791	117

Timor-Leste	1	1	0	1	1	0

Turkey	1	0	1	1	0	1

United Arab Emirates	1	1	0	1	1	0

Vanuatu	36	33	3	36	33	3

Viet Nam	275	255	20	334	314	20

Yemen	14	9	5	19	9	10

**Total**	**8853**	**7430**	**1423**	**15410**	**12353**	**3057**

'Data' include points (≤10 km^2^) and wide areas (10-25 km^2^) both of which are used in the BRT model and displayed on the predictive maps (Additional file 3). 'Polygons' include small (25-100 km^2^) and large (>100 km^2^) polygons which are not included in the models or shown on the maps.

Focussing only on the geo-referenced data, 7430 sites (12353 occurrences) were reported at a resolution that could be applied to the BRT models, including 6952 points (<10 km^2^) and 478 wide-areas (between 10-25 km^2^). Data from small and large polygons (25-100 km^2 ^and >100 km^2 ^respectively) were not used in the BRT models.

Of the 31 countries with a reported presence of one or more of the 19 DVS, the greatest number of sites were across Myanmar, including 1791 mappable locations (i.e. both points and wide areas collectively, henceforth referred to as 'points'). The greatest number of occurrences (3935) were reported from India; however, this included a very high number reported from polygons (2002), so the number of occurrences from points (1933) was subsequently lower than those from Myanmar (2724 points from 2777 total occurrences) (Table 3). Four countries only had a single site where DVS presence was reported (Singapore: *An. maculatus *subgroup, Timor-Leste: *An. subpictus *complex, Turkey: *An. barbirostris *and United Arab Emirates: *An. stephensi*).

The *An. farauti *complex was reported from the greatest number of independent sites, of which 1465 were points (Table 4). This was due, in part, to the inclusion of two comprehensive surveys in Papua New Guinea (PNG) [31] and northern Australia [30], which provided a total of 846 points. *Anopheles leucosphyrus*/*An. latens *were identified from the fewest number of locations (14, of which 12 were points), with *An. balabacensis *also reported from relatively few sites (17, of which 14 were points) (Table 4).

**Table 4:** Geo-referenced and non geo-referenced data by species and area type: 'Point'  is all mapped data included in the BRT model: point (≤10 km^2^), wide areas  (10-25 km^2^) and 'Polygon' details data not incorporated in BRT model: small (25-100 km^2^) and large (>100 km2) polygons, for the 19 Asian-Pacific DVS (geographically independent sites (Site) and temporal independent occurrences (Occ)).

	Geo-referenced				Non geo-referenced			
	**Point and wide** **area ('Point')**		**Polygon**		**Point and wide** **area ('Point')**		**Polygon**	

**Species**	**Site**	**Occ**	**Site**	**Occ**	**Site**	**Occ**	**Site**	**Occ**

*An. aconitus*	424	616	74	115	42	54	32	67

*An. annularis*	496	851	156	332	82	188	32	87

*An. balabacensis*	14	42	3	3	4	9	2	8

An. barbirostris*	872	1064	69	93	70	94	24	56

An. culicifacies*	550	1568	271	774	178	930	64	371

An. dirus*	372	727	60	87	31	60	12	26

An. farauti*	1465	1737	25	28	35	50	1	1

*An. flavirostris*	103	122	11	33	4	4	4	4

An. fluviatilis*	83	318	138	352	80	330	27	149

*An. koliensis*	325	363	7	7	24	26	2	3

*An. lesteri*	47	80	65	89	17	18	8	14

*An. leucosphyrus & An. latens*	12	12	2	2	2	2	0	0

*An. maculatus *group	471	765	83	145	75	188	24	113

An. minimus*	445	711	93	111	75	113	59	153

An. punctulatus*	379	581	9	26	30	42	2	3

An. sinensis*	568	792	121	293	43	108	12	22

*An. stephensi*	261	646	81	220	19	41	12	59

An. subpictus*	410	1143	127	317	87	219	27	67

An. sundaicus*	133	215	28	30	12	12	9	9

**Total**	**7430**	**12353**	**1423**	**3057**	**910**	**2488**	**353**	**1212**

An asterisk (*) denotes that a "species" is now recognised as a species complex.

Myanmar appeared to contain the greatest number of DVS, with 10 species recorded at 16 sites. These included the concurrent presence of *An. aconitus*, *An. annularis*, *An. barbirostris *complex, *An. culicifacies *complex, *An. dirus *complex, Maculatus Group, *An. minimus *complex, *An. sinensis *complex, *An. stephensi *(replaced by *An. sundaicus *complex in some coastal sites) and *An. subpictus *complex [22,78].

Larval collection was by far the most popular sampling method employed in the capture of the DVS from the Asian-Pacific region with 199 sources reporting this sampling method from 2123 sites. A total of 84 sources did not mention the sampling methods used at one or more of their sampled sites (1349 sites).

Of those studies that described the methodologies applied in species identification, morphological examination was the most common and was used on samples from 3766 sites, reported from 265 sources.

### Mapping trials

The mapping trials, evaluated visually and guided by the statistical metric output of the BRT models, indicated that the 'optimal' output for the Asian Pacific species maps included a 1500 km buffer where pseudo-absences at a ratio of 10:1 (pseudo-absences to presence data) were randomly allocated. The 'hybrid' model, where occurrence data were supplemented with 500 half-weighted pseudo-presences allocated within the EO species range, created maps with a much greater predictive performance than those based on 1000 pseudo-presences randomly allocated in the EO range alone. Those maps produced using the occurrence data alone, without any supplemental pseudo-presences, produced the lowest, and therefore 'better' deviance values. However, on inspection, the maps were judged to be visually poorer, with less predictive value, specifically for those species with limited available data (e.g. *An. leucosphyrus*/*An. latens*, *An. balabacensis*). This 'hybrid' method, was also judged to provide the best maps for those DVS found in Africa, Europe, the Middle East and the Americas [43,79] suggesting that, especially in areas where data is sparse, an educated addition of pseudo-presences can greatly improve the final mapping output. The results for each mapping trial conducted are provided in Additional file 2 (Additional file 2: Summary tables showing evaluation statistics for all mapping trials and final BRT environmental and climatic variable selections for the final, optimal predictive maps).

### Predictive maps

**Figure 1:**
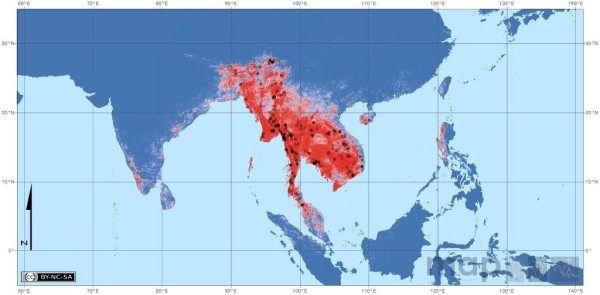
**Map details: The predicted distribution of the *Anopheles dirus* species complex mapped using hybrid data (372 occurrence data plus 500 pseudo-presences weighted at half that of the occurrence data and randomly selected from within the Expert Opinion (EO) range).** Pseudo-absences (3720) were generated at a ratio of 10:1 absence to presence points, and were randomly selected from within the 1500 km buffer surrounding the EO (EO shown in the inset map). Predictions are not shown beyond the buffer boundary. The black dots show the 372 occurrence records for the complex. Map statistics: Deviance = 0.1738, Correlation = 0.8793, Discrimination (AUC) = 0.9857, Kappa = 0.8451. Environmental variables: 1. LST (P1), 2. Prec (A1), 3. MIR (P1), 4. NDVI (mean), 5. LST (P2), (see Additional file 2 for abbreviations and definitions). Copyright: Licensed to the Malaria Atlas Project [520] under a Creative Commons Attribution 3.0 License. Citation: Sinka *et al*. (2011) The dominant *Anopheles *vectors of human malaria in the Asia Pacific region: occurrence data, distribution maps and bionomic précis, *Parasites & Vectors *2011, **4**:89.

The final predictive maps for the 19 Asian-Pacific DVS are given in Additional file 3 (Additional file 3: Predictive species distribution maps for the 19 DVS of the Asian-Pacific region). Spatial constraints prevent all species being discussed in detail, however, the *An. dirus *complex (Figure 1), due to its longevity and the highly anthropophilic behaviour of its members, is considered to be the dominant vector group in any area where its species exist (Manguin, unpub obs) and, therefore, is discussed below.

The maps produced for the *An. dirus *complex (and for all other species complexes) do not differentiate between the members of the complex. Simply, this is due to a lack of consistent sibling species identification reported in the current literature, a status that will improve with the increasing development of reliable molecular identification methods and the rising acceptance that understanding bionomics differences in sibling species is a prerequisite for efficient control.

Behaviourally, the members of the *An. dirus *complex have clear differences (see below), but ecologically, they tend to occupy the same ecological niche and are generally considered as forest-dwelling species, specifically in mountainous areas and foothills, with an affinity for humid, shaded environments where they make use of transient or temporary larval habitats such as pools and puddles.

The phase of the annual cycle of LST (LST (P1)) was selected by the BRT model as the most influential variable (58.9%). Interestingly, for all *An. dirus *complex outputs in each of the mapping trials conducted, this variable was always found to be the most influential. LST (P1) was also chosen as the primary influence for the distribution of *An. aconitus *(47.55%), *An. annularis *(37.14%), the *An. maculatus *group (64.0%) and the *An. subpictus *complex (34.21%) and, to a slightly lesser degree, for *An. minimus *complex (31.46%). The common characteristic to each of these species and species complexes appears to be a distribution that includes hilly or forested hilly areas, both of which (altitude and an increased evapotranspiration rate over forest, specifically during the rainy season) could be highly influential in any satellite-derived LST measurement.

The amplitude of the annual cycle of precipitation, ranked second by the BRT model, exerted an influence of 10.47% on the distribution of the *An. dirus *complex. This corresponds to the suggested influence of seasonal LST (P1), and to the creation of temporary pools and puddles utilised for female oviposition and as a habitat for the immatures of the *An. dirus *complex.

The final three variables selected within the top five, in ranked order, are MIR, the phase of the annual cycle (8.70%); mean NDVI (7.91%); and LST, phase of the biannual cycle (3.95%). MIR discriminates land cover and is able to penetrate better through aerosol particles, including water, and is thus able to distinguish between vegetation, soil, rock and water [61] which, alongside the NDVI, may also refer to the influence of the forested areas in the distribution of the *An. dirus *complex.

### Bionomics

Tables 5, 6, 7, 8, 9, 10, 11, 12 show a summary of larval and adult bionomics data extracted from individual studies reported in the literature ('Summary') combined with the common 'accepted' bionomics of each species ('TAG').

**Table 5:** Larval site characteristics.

		Light intensity		Salinity		Turbidity		Movement		Vegetation	
		
Species	Source	Helio- philic	Helio- phobic	High (brackish)	Low (fresh)	Clear	Turbid	Still or stagnant	Flowing	Higher plants, algae etc	No Veg
*An. aconitus*	Summary	-	-	-	-	1	1	-	3	2	1

*An. aconitus*	TAG	●			●	●	○	●	○	●	

*An. annularis*	Summary	1	-	-	1	1	1	1	2	7	2

*An. annularis*	TAG	●			●	●	○	●	○	●	○

*An. balabacensis*	Summary	-	1	-	-	-	-	-	-	-	1

*An. balabacensis*	TAG	○	●		●			●		○	○

An. barbirostris*	Summary	2	1	-	-	4	3	1	1	5	2

An. barbirostris*	TAG	●	●			●	○			●	

An. culicifacies*	Summary	2	-	1	2	3	1	3	3	3	2

An. culicifacies*	TAG	●		○	●	●	○	●	●	●	●

An. dirus*	Summary	1	10	-	2	4	3	1	-	-	2

An. dirus*	TAG		●		●	●	●	●	○		●

An. farauti*	Summary	1	6	8	14	2	4	2	1	18	2

An. farauti*	TAG	●	○	●	●	●	○	●		●	●

*An. flavirostris*	Summary	-	2	-	-	-	-	-	2	1	1

*An. flavirostris*	TAG		●		●	●		○	●	●	○

An. fluviatilis*	Summary	1	-	-	1	-	-	1	3	2	1

An. fluviatilis*	TAG	●			●			○	●	●	○

*An. koliensis*	Summary	-	-	-	1	-	-	-	-	1	1

*An. koliensis*	TAG	●	○		●	●		●		●	○

TAG: ● = typical, ○ = examples exist.

Numbers indicate the number of studies that found larvae under each listed circumstance. An asterisk (*) denotes that a "species" is now recognised as a species complex.

**Table 6:** Larval site characteristics (cont.).

		Light intensity		Salinity		Turbidity		Movement		Vegetation	
		
Species	Source	Helio- philic	Helio- phobic	High (brackish)	Low (fresh)	Clear	Turbid	Still or stagnant	Flowing	Higher plants, algae etc	No Veg
*An. lesteri*	Summary	-	1	-	-	-	-	-	-	1	-

*An. lesteri*	TAG				●						

*An. leucosphyrus & An. latens*	Summary	-	-	-	-	-	-	-	-	-	-

*An. leucosphyrus & An. latens*	TAG		●		●	●	●	●			

*An. maculatus *group	Summary	2	1	-	-	2	1	2	1	1	1

*An. maculatus *group	TAG	●			●	●		●	●	●	

An. minimus*	Summary	1	1	-	-	2	-	3	2	3	1

An. minimus*	TAG	○	●		●	●		●	●	●	

An. punctulatus*	Summary	3	-	-	1	2	1	-	-	2	4

An. punctulatus*	TAG	●	○		●	●	●	●		○	●

An. sinensis*	Summary	-	-	-	-	1	-	2	2	2	1

An. sinensis*	TAG				●	●		●	○	●	

*An. stephensi*	Summary	2	1	1	1	3	2	2	1	2	1

*An. stephensi*	TAG	○	●	○	●	●	●	●		●	●

An. subpictus*	Summary	2	-	7	4	3	4	2	1	6	2

An. subpictus*	TAG	●		●	●	●	●	●		●	

An. sundaicus*	Summary	3	1	7	4	1	-	1	-	2	1

An. sundaicus*	TAG	●	○	●	○	●	●	●	○	●	○

TAG: ● = typical, ○ = examples exist.

Numbers indicate the number of studies that found larvae under each listed circumstance. An asterisk (*) denotes that a "species" is now recognised as a species complex.

**Table 7:** Large larval sites.

		Large natural water collections					Large man-made water collections				
		
Species	Source	Lagoons	Lakes	Marshes	Slow flowing rivers	Other	Borrow pits	Rice fields	Fish ponds	Irrigation channels	Other
*An. aconitus*	Summary	-	1	-	2	3	-	5	1	2	2

*An. aconitus*	TAG		○	●	○		○	●	●	●	

*An. annularis*	Summary	-	-	1	4	2	-	14	-	4	6

*An. annularis*	TAG			●				●			

*An. balabacensis*	Summary	-	-	1	-	-	-	1	-	-	-

*An. balabacensis*	TAG							○			

An. barbirostris*	Summary	-	-	3	5	4	-	17	3	5	6

An. barbirostris*	TAG		●	●	●			●	●	●	

An. culicifacies*	Summary	-	1	1	5	3	-	14	-	7	5

An. culicifacies*	TAG				●			●		●	

An. dirus*	Summary	-	-	-	-	3	-	3	1	-	-

An. dirus*	TAG							○			

An. farauti*	Summary	5	-	-	2	11	-	2	2	-	3

An. farauti*	TAG	●	○	●	○		○	○	○	○	

*An. flavirostris*	Summary	-	-	-	1	-	-	-	-	-	-

*An. flavirostris*	TAG				○			○	○	○	

An. fluviatilis*	Summary	-	-	-	5	2	-	6	-	1	-

An. fluviatilis*	TAG				●			●		●	

*An. koliensis*	Summary	-	-	-	-	-	-	-	1	-	1

*An. koliensis*	TAG	○		●			○		○	●	

TAG: ● = typical, ○ = examples exist.

Numbers indicate the number of studies that found larvae under each listed circumstance. An asterisk (*) denotes that a "species" is now recognised as a species complex.

**Table 8:** Large larval sites (cont.).

		Large natural water collections					Large man-made water collections				
		
Species	Source	Lagoons	Lakes	Marshes	Slow flowing rivers	Other	Borrow pits	Rice fields	Fish ponds	Irrigation channels	Other
*An. lesteri*	Summary	-	-	-	-	4	-	3	-	1	1
*An. lesteri*	TAG		●	●				●			
*An. leucosphyrus & An. latens*	Summary	-	-	-	-	-	-	-	-	-	-
*An. leucosphyrus & An. latens*	TAG										
*An. maculatus *group	Summary	-	-	-	3	4	-	8	-	1	-
*An. maculatus *group	TAG		●					●		●	
An. minimus*	Summary	-	-	-	-	5	-	8	5	1	2
An. minimus*	TAG							●	●		
An. punctulatus*	Summary	-	-	-	-	1	-	-	1	-	-
An. punctulatus*	TAG								○		
An. sinensis*	Summary	-	-	1	-	3	-	11	-	2	2
An. sinensis*	TAG			●				●		●	
*An. stephensi*	Summary	-	1	1	5	3	-	5	-	4	3
*An. stephensi*	TAG				●			●		●	
An. subpictus*	Summary	1	-	2	3	4	-	13	-	6	5
An. subpictus*	TAG	●		●				●		●	
An. sundaicus*	Summary	-	-	2	-	3	-	2	1	-	1
An. sundaicus*	TAG	●		●					●		

TAG: ● = typical, ○ = examples exist.

Numbers indicate the number of studies that found larvae under each listed circumstance. An asterisk (*) denotes that a "species" is now recognised as a species complex.

**Table 9:** Small larval sites.

		Small natural water collections						Small man-made water collections							Artificial sites
		
Species	Source	Small streams	Seepage springs	Pools	Wells	Dips in the ground	Other	Overflow water	Irrigation ditches	Borrow pits	Wheel ruts	Hoof prints	Puddles near rice fields	Other	Empty cans, shells **etc**.
*An. aconitus*	Summary	3	-	5	2	1	-	-	-	2	1	1	-	1	-

*An. aconitus*	TAG	○	○	●	○	○	○	○	●	○	○	○	○		○

*An. annularis*	Summary	2	-	10	4	-	1	-	1	2	1	2	1	1	1

*An. annularis*	TAG	○	○	●	○				○	○	○	○	○		

*An. balabacensis*	Summary	-	-	1	-	-	-	-	-	-	-	-	-	-	-

*An. balabacensis*	TAG	○		●		●			●		●	●	○		○

An. barbirostris*	Summary	10	1	13	5	1	3	-	-	2	1	1	-	2	-

An. barbirostris*	TAG	●	●	●	●						●	●			

An. culicifacies*	Summary	5	-	14	5	-	5	-	2	5	1	4	1	4	2

An. culicifacies*	TAG	●		●	●				●	●	●	●	●		

An. dirus*	Summary	3	-	13	4	1	3	-	-	1	5	4	-	3	-

An. dirus*	TAG	●		●	●	●				●	●	●			

An. farauti*	Summary	10	-	22	3	-	8	-	-	1	10	7	-	12	-

An. farauti*	TAG			●		○		○	○	○	○	○			○

*An. flavirostris*	Summary	5	-	1	-	-	-	-	-	-	-	-	-	-	-

*An. flavirostris*	TAG	●	○	○	○	○		○	○						

An. fluviatilis*	Summary	7	-	3	3	-	4	-	2	3	-	1	-	2	-

An. fluviatilis*	TAG	●		○	○				●	○					

*An. koliensis*	Summary	1	-	1	1	-	1	-	-	-	1	1	-	1	-

*An. koliensis*	TAG	○		●		●			●	○	○	○			

TAG: ● = typical, ○ = examples exist.

Numbers indicate the number of studies that found larvae under each listed circumstance. An asterisk (*) denotes that a "species" is now recognised as a species complex.

**Table 10:** Small larval sites (cont.).

		Small natural water collections						Small man-made water collections							Artificial sites
		
Species	Source	Small streams	Seepage springs	Pools	Wells	Dips in the ground	Other	Overflow water	Irrigation ditches	Borrow pits	Wheel ruts	Hoof prints	Puddles near rice fields	Other	Empty cans, **shells etc**.
*An. lesteri*	Summary	1	-	4	-	-	1	-	2	-	1	-	-	2	-

*An. lesteri*	TAG	●		●					●						

*An. leucosphyrus & An. latens*	Summary	-	-	-	-	-	-	-	-	-	-	-	-	-	-

*An. leucosphyrus & An. latens*	TAG	●	●	●							●	●			

*An. maculatus *group	Summary	10	2	9	1	2	5	-	-	-	1	1	-	1	-

*An. maculatus *group	TAG	●	●	●	●	●					●	●			

An. minimus*	Summary	18	2	9	1	1	4	-	-	-	2	1	3	1	-

An. minimus*	TAG	●	●	●											

An. punctulatus*	Summary	3	-	4	1	1	5	-	-	-	4	1	-	3	-

An. punctulatus*	TAG	○		○		●		○	○	●	●	●			○

An. sinensis*	Summary	4	-	7	-	1	2	-	4	-	1	-	-	2	3

An. sinensis*	TAG	●	●	●					●		●				

*An. stephensi*	Summary	2	-	9	9	1	5	1	1	4	1	3	-	14	11

*An. stephensi*	TAG	●		●	●							○			●

An. subpictus*	Summary	2	-	10	4	-	2	-	2	2	1	2	-	5	2

An. subpictus*	TAG			●	●				●						●

An. sundaicus*	Summary	3	-	2	2	-	1	-	-	1	-	-	-	3	-

An. sundaicus*	TAG			●											

TAG: ● = typical, ○ = examples exist.

Numbers indicate the number of studies that found larvae under each listed circumstance. An asterisk (*) denotes that a "species" is now recognised as a species complex.

**Table 11:** Adult feeding and resting behaviour.

		Feeding habit		Biting habit		Biting time				Pre-feeding resting habit		Post-feeding resting habit	
		
Species	Source	Anthro- pophilic	Zoo- philic	Exo- phagic	Endo- phagic	Day	Dusk	Night	Dawn	Exo- philic	Endo- philic	Exo- philic	Endo- philic
*An. aconitus*	Summary	1	3	4	3	-	2	5	-	-	1	1	2

*An. aconitus*	TAG	○	●	●	●		○	●		●	○	●	○

*An. annularis*	Summary	-	8	4	1	-	3	5	-	1	3	1	5

*An. annularis*	TAG	○	●	●	●		○	●		○	●	○	●

*An. balabacensis*	Summary	4	-	3	2	-	1	4	-	-	1	-	2

*An. balabacensis*	TAG	●		●	●		●	●		○	○	○	○

An. barbirostris*	Summary	-	3	2	1	-	5	3	-	2	1	3	2

An. barbirostris*	TAG	○	●	●	○		●	●		●	○	●	○

An. culicifacies*	Summary	-	9	2	2	-	3	11	-	1	4	1	5

An. culicifacies*	TAG	●	●	●	●		●	●		○	●	○	●

An. dirus*	Summary	12	1	10	7	-	-	17	-	2	1	2	-

An. dirus*	TAG	●		●	●		○	●		●		●	

An. farauti*	Summary	5	-	3	-	-	5	10	1	1	-	1	-

An. farauti*	TAG	●	○	●	●	○	○	●		●	○	●	○

*An. flavirostris*	Summary	2	4	6	3	-	-	4	-	-	1	1	1

*An. flavirostris*	TAG	●	●	●	●			●		●	○	●	

An. fluviatilis*	Summary	5	6	3	4	-	2	5	1	3	5	5	7

An. fluviatilis*	TAG	●	●	●	●		●	●	○				

*An. koliensis*	Summary	3	-	4	6	-	1	6	1	-	-	-	-

*An. koliensis*	TAG	●	○	●	●	○	○	●	○	●	○	●	○

TAG: ● = typical, ○ = examples exist.

Numbers indicate the number of studies that found adults under each listed circumstance. An asteriosk (*) denotes that a "species" is now recognised as a species complex.

**Table 12:** Adult feeding and resting behaviour (cont.).

		Feeding habit		Biting habit		Biting time				Pre-feeding resting habit		Post-feeding resting habit	
		
Species	Source	Anthro- pophilic	Zoo- philic	Exo- phagic	Endo- phagic	Day	Dusk	Night	Dawn	Exo- philic	Endo- philic	Exo- philic	Endo- philic
*An. lesteri*	Summary	2	2	-	-	-	1	1	-	-	2	-	2

*An. lesteri*	TAG	●	●				●	●			●		●

*An. leucosphyrus & An. latens*	Summary	-	-	-	-	-	1	2	-	-	-	-	-

*An. leucosphyrus & An. latens*	TAG	●		●	●		○	●		●		●	

*An. maculatus *group	Summary	1	9	12	4	-	7	12	1	2	1	3	1

*An. maculatus *group	TAG	○	●	●	●		●	●					

An. minimus*	Summary	6	14	12	6	-	6	12	1	3	2	4	4

An. minimus*	TAG	●	●	●	●		●	●	○	●	●	●	●

An. punctulatus*	Summary	2	-	3	3	-	-	7	2	-	-	-	-

An. punctulatus*	TAG	●	○	●	●	○	○	●	○	●	○	●	○

An. sinensis*	Summary	1	9	2	-	-	3	5	-	1	-	1	-

An. sinensis*	TAG		●	●			●	●		●		●	

*An. stephensi*	Summary	2	4	-	-	-	1	2	-	-	8	-	8

*An. stephensi*	TAG	○	●	○	●		●	●			●		●

An. subpictus*	Summary	1	9	3	2	-	6	5	-	1	6	1	7

An. subpictus*	TAG		●	●	●		●	●			●		●

An. sundaicus*	Summary	1	1	1	3	-	-	3	-	1	1	1	1

An. sundaicus*	TAG	●	○	●	●			●		●	●	●	●

TAG: ● = typical, ○ = examples exist.

Numbers indicate the number of studies that found adults under each listed circumstance. An asterisk (*) denotes that a "species" is now recognised as a species complex.

### *Anopheles *(*Anopheles*) *barbirostris *van der Wulp species complex (Barbirostris Complex)

*Anopheles barbirostris *and 12 related species comprise the medically important and taxonomically complex Barbirostris Group of malaria vectors in the Oriental Region. Six of these species, including *An. barbirostris*, comprise the Barbirostris Subgroup of species that are almost identical in adult morphology but differ in their roles in the transmission of malaria and filariasis in Southeast Asia.

Mosquitoes traditionally identified as *An. barbirostris *are common and widely distributed from India through mainland Southeast Asia and southward through Indonesia to Sulawesi, all of the Lesser Sunda island chain to Timor Island and possibly the eastern fringe of the Maluku (Mollucas) archipelago [80,81]. Records of this species in the Maluku region and New Guinea are questionable and are more likely to be misidentifications of related species, notably *An. vanus *Walker. Published records of *An. barbirostris *in the Philippines refer to other species of the Barbirostris Group [82]. *Anopheles barbirostris *is generally found in highland areas [81,83], but in western Timor it is considered a coastal species [83].

Sequence data for the COI mtDNA gene and the ITS2 rDNA locus [84-86], as well as electrophoretic profiles of salivary gland proteins [87], indicate that *An. barbirostris *is a complex of three to five sibling species with undefined distributions. The question of how many species constitute the Barbirostris Complex needs to be resolved because correct recognition and identification has important implications in the choice of methods applied to malaria and lymphatic filariasis control. *Anopheles barbirostris *is considered an important vector of malaria and Brugian filariasis in Sulawesi, Flores and Timor [88-92], whereas it appears to be a non-vector in other regions [82]. A recent survey in northern Sumatra identified *An. barbirostris *as a potential vector of malaria [93], and Limrat *et al*. [94] and Apiwathnasorn *et al*. [95] reported that either *An. barbirostris *or *An. campestris *Reid (these species could not be reliably distinguished) is a probable vector of malaria in Sa Kaeo Province of Thailand where high numbers of females were captured landing on humans both indoors and outdoors.

Saeung *et al*. [84,85] provided strong evidence for at least two species within the Barbirostris Subgroup based on a series of cross-mating experiments (also Choochote *et al*. [96]), cytogenetic studies and sequence analysis of ITS2, COI and COII using isolines derived from wild-caught females. Unfortunately, a comparison of COI sequence data obtained by Paredes-Esquivel *et al*. [86] with those from Saeung *et al*. [85] proved to be impossible because the regions sequenced do not overlap. The A3 form of Saeung *et al*. [85] has a much smaller ITS2 amplicon than the corresponding region investigated by Paredes-Esquivel *et al*. [86], suggesting that it is not closely related to *An. barbirostris s.l*. Sequence comparisons showed that Clades I and II of Paredes-Esquivel *et al*. [86] were not included in the analyses of Saeung *et al*. [85], and that Clades III and V of Paredes-Esquivel *et al*. [86] correspond to form A1 and *An. campestris *of Saeung *et al*. [85], which they described as a zoophilic and more anthropophilic species, respectively. Zoophilic and anthropophilic forms of *An. barbirostris *were previously reported by Lien *et al*. [90], but these behavioural differences, which would influence their capacity to transmit malaria protozoa or filarial nematodes, could not be associated with distinct morphological characters [91] and may only reflect relative availability of different hosts. Saeung *et al*. [85] identified specimens with ITS2 sequences similar to Clade IV of Paredes-Esquivel *et al*. [86] as *An. barbirostris*, however specimens of Clade IV are morphologically distinct from *An. barbirostris*. Based on available data, it is not possible to determine which genetic species correspond to vector populations. Further analyses require extensive sampling in areas where *An. barbirostris *has been reported to be anthropophilic, such as Sulawesi [90] and Flores [91]. Molecular analyses indicate that Clades I and II of Paredes-Esquivel *et al*. [86] occur in the type locality of *An. barbirostris *in eastern Java (H. Townson & R. Harbach, pers com), but which of these two genetic species is conspecific with *An. barbirostris s.s*. is unknown.

Females of *An. barbirostris s.l*. bite humans but generally prefer to feed on other animals, especially bovids [81,91,97-99]. Feeding apparently takes place outdoors, but adults have been collected resting inside houses and animal shelters as well as outside [81]. Outdoor biting in peninsular Malaysia near the Thai border takes place throughout the night [100] (Table 11). Reports that *An. barbirostris *is a vector of malarial and filarial parasites came before the recognition of the Barbirostris Complex, and these reports may refer to other species of very similar morphology. In view of the feeding preferences and behaviour of females, *An. barbirostris s.s*. probably plays little if any role in the transmission of malaria and filariasis in most areas where it occurs. Since Clades III and IV of Paredes-Esquivel *et al*. [86] appear to be predominantly zoophilic, they may be of limited importance in the transmission of human pathogens. Unfortunately, there is very limited information on the habitats of Clades I and II, and none on their blood-feeding preferences. *Anopheles barbirostris s.l*. is a confirmed vector of *P. falciparum *malaria in Sri Lanka [99] and Timor-Leste [92] based on the enzyme-linked immunosorbent assay (ELISA) detection of sporozoites in the head-thorax portions of females, which in the case of the latter study were collected in human-landing catches. Both *P. vivax *and *P. falciparum *have been detected by ELISA in females of *An. barbirostris s.l*. in Bangladesh, but it is not known whether sporozoites, oocysts or both were present as whole mosquitoes were assayed for infection [101].

Larvae can occupy a great variety of aquatic habitats throughout the range of the complex. *Anopheles barbirostris s.l*. is a swamp breeder, typically found in deep fresh water that is still or slow moving. However, it is not uncommon in or near rice fields and is tolerant of relatively high levels of organic pollution including sewage, and can be found in ground pools with high concentrations of animal dung. Other habitats vary from sunlit to moderately shaded ground-water bodies, including river and stream margins and pools, ditches, moats, lakes, permanent and temporary ground pools, rice fields, wells, canals, marshes, rock pools, ponds, springs, swamps and animal footprints. The habitats usually contain some vegetation [80,81] (Tables 5, 7, 9).

### *Anopheles *(*Anopheles*) *lesteri *Baisas & Hu

*Anopheles lesteri*, originally described from Luzon Island in the Philippines, is a member of the Hyrcanus Group of mosquitoes within the Myzorhynchus Series [6]. Xu & Feng [102] described and named *An. anthropophagus *as a subspecies of *An*. *lesteri *from mosquitoes collected in Jhangsu (as Kiangsu) Province, China. Ma [103] raised *anthropophagus *to species status based on its morphology, distribution and vectorial capacity. Wilkerson *et al*. [104], however, synonymised *An. anthropophagus *with *An. lesteri *based on identical ITS2 sequences found in *An. lesteri *from its type locality in Laguna Province, the Philippines, and *An. anthropophagus *from Jhangsu Province, China. Ma & Xu [105] compared ITS2 sequences among 12 species of the Hyrcanus Group in China and agreed that *An. anthropophagus *is a synonym of *An. lesteri*. Likewise, Hwang *et al*. [106] reported that sequences of the ITS2 region provide strong evidence that *An. lesteri *in Japan and *An. anthropophagus *in China are the same species.

*Anopheles lesteri *readily attacks humans and is considered a primary vector of malaria in eastern, central and southern areas of China [102,103,107-109] [as *An*. *anthropophagus*], and is believed to be a principal vector in Japan and Korea [110-112]. Shin *et al*. [113] showed that *An. lesteri *was able to develop sporozoites of *P. vivax *after feeding on a Korean malaria patient. Joshi *et al*. [114] detected high densities of sporozoites in salivary glands of *An. lesteri *infected with the Korean strain of *P. vivax *and concluded that *An. lesteri *should be a highly competent vector of *P. vivax *malaria provided its survival is sufficiently long in the field. Molecular [115] and morphological identifications [116] have extended the distribution of *An. lesteri *northward in China to 42° N 120° W and 42.5° N 123.41° W, respectively; localities which are farther north than the Korean Peninsula. The biting behaviour of *An. lesteri *is unknown in the Philippines and Guam, but it is not known to transmit malarial parasites in these areas [117]. This species is also regarded as an important vector of lymphatic filariasis (*B. malayi*) in China [118].

Larvae of *An. lesteri *are found in fresh-water ground pools, ditches, margins of streams and ponds, rice fields, marshes, swamps, lakes and other impounded waters [112,119-121] (Tables 6, 8, 10). Adults of *An. lesteri *rest in cool and shaded places. Adult populations reach peak densities during the summer in Hokkaido [122], and during June and October in Honshu and Kyushu, Japan [123]. In Hong Kong, *An. lesteri *commonly occurs in hilly areas and grassy fields [124]. Basio & Reisen [125] found larvae in a wallow on Guam and Whang [126] collected adults in cow sheds and houses in villages during malaria surveys in Korea. *Anopheles lesteri *has been confused with *An. sinensis *and other members of the Hyrcanus Group, and some published records of its distribution and bionomics are likely not to be accurate, particularly in Japan, Korea and China [117].

### *Anopheles *(*Anopheles*) *sinensis *Wiedemann

*Anopheles sinensis *is also a member of the Hyrcanus Group in the Myzorhynchus Series [6]. It is widely distributed in southern Asia from Afghanistan to northern China, Japan, Korea, Taiwan and southward into western Indonesia (Sumatra and West Kalimantan) [81,112,127-130]. There is evidence that *An. sinensis *is refractory to *P. falciparum *[131,132], but it is still considered an important vector of *P. vivax *malaria (and *B. malayi*) in both China and Korea [104,108,118,133-137]. It is the most common anopheline species in Japan [112,138], where it is regarded as an important 'historical' vector of malaria [123]. *Anopheles sinensis *is also considered to be a minor malaria vector in Indonesia (Sumatra only) [130]. *Anopheles sinensis *has little or no involvement in malaria transmission in Thailand due to its zoophilic and exophilic behaviour and its prevalence primarily in areas where there is little or no malaria [81] (Table 12).

Along the border between North and South Korea, Strickman *et al*. [139] reported that *An. sinensis *(based on morphological identifications) comprised 80% of the anopheline mosquitoes attacking humans during an outbreak of *P. vivax *malaria. Lee *et al*. [140] found that a third of *An. sinensis *females that fed on volunteers infected with a Korean strain of *P. vivax *became infective based on the ELISA detection of sporozoites. The mosquitoes were collected in a non-epidemic area of the country to ensure that uninfected mosquitoes were used in the study. Joshi *et al*. [114] performed feeding experiments with the Korean strain of *P. vivax *and found that *An. sinensis *females develop oocysts but only a reduced number of sporozoites were detected in salivary glands compared with females of *An. lesteri*.

Lee *et al*. [141] found that *An. sinensis *delivered a relatively high biting rate (87.6 bites/person/night) during human-bait collections conducted in Paju, South Korea. The parity of *An. sinensis *from human-bait collections fluctuated from 41-71% (mean 48.8%) from June (mean 35.2%) to July (mean 55.0%) and August (mean 66.2%). From these data, Lee *et al*. [141] estimated that the probability of daily survival of *An*. *sinensis *in the summer season is 0.79, with an assumed three-day gonotrophic cycle [142] and the expectancy of infective life through 11 days as 0.073. In contrast, Ree *et al*. [142] calculated parity rates of 75.2% in July, 56.5% in August, 78.5% in September and 60.0% in October, and a slightly higher probability of daily survival rate (0.89) for *An. sinensis *in Gyonggi-do, South Korea. Based on blood meal analysis (ELISA), Lee *et al*. [141] reported that only 0.8% of *An. sinensis *females obtained blood meals from human hosts, as opposed to 61.8% from cows. In comparison, Ree *et al*. [142] found that only 0.7% of females had fed on humans, 89.8% on bovines and the remaining on either swine (3.3%), dogs (0.7%), chickens (1.6%) or both bovines and swine (0.7%). Both studies concluded that the malaria transmission potential of *An. sinensis *is very low despite the high number of females that attack humans, i.e. vectorial capacity would be high only in the presence of large population densities. Chang *et al*. [143] reported that females of *An. sinensis *collected from resting sites in villages in Taiwan, 86.4% were found to have fed on pig, 9.1% on bovine and 4.5% on horse, as determined by polymerase chain reaction (PCR) analyses of blood meals. Females were routinely collected outside human dwellings and near larval habits, but none were collected inside human habitations. Mwandawiro *et al*. [144] studied the host preferences of *An. sinensis *females in an extensive rice growing area at Nishi Arita, Saga Prefecture, Japan by collecting resting mosquitoes from animal sheds. Blood meal analysis (ELISA) found that females preferred cows and pigs to chickens in both terraced hill-side and rice field locations. None were found to have fed on humans or dogs.

Lee *et al*. [145] found *An. sinensis *to be the most abundant *Anopheles *mosquito captured from cowshed resting sites in both high and low-risk malaria areas in South Korea. However, *An. pullus *Yamada and *An. kleini *Rueda had higher concentrations of circumsporozoite antigen for *P. vivax *when analysed by ELISA, indicating greater numbers of sporozoites present in salivary glands. Moreover, *An. kleini *and *An. pullus *developed higher infection rates than *An. sinensis *in laboratory studies by feeding on malaria-infected blood from patients. The findings suggest that *An. sinensis *is a less effective vector of malaria in Korea than other members of the Hyrcanus Group.

*Anopheles sinensis *is prevalent in Korea from late April/early May to October, with populations peaking in late June to mid-July and declining in August [128,133]. The species is present throughout the year in southern Taiwan, with peak densities in spring (February-March) and autumn (September-October) that coincide with the two periods of rice cultivation [133].

Female *An. sinensis *feed throughout the night, with peak activity apparently occurring at different hours depending on locality [108,128,133,139]. Whang [146] observed that the biting activity of *An. sinensis *is influenced by wind speed and direction. Under normal circumstances, females are predominantly zoophilic and exophilic, infrequently biting humans in the presence of their preferred hosts (buffalo and cattle), and are rarely found inside human habitations (Table 12).

The immature stages of *An. sinensis *are primarily found in lowland, shallow, fresh-water habitats with emergent and/or floating vegetation in open agriculture lands (mainly rice fields). They also utilise stream margins, irrigation ditches, ponds, marshes, swamps, bogs, pits, stump ground holes, grassy pools, flood pools, stream pools, rock pools, seepage-springs and wheel tracks [29,81,112,128,130,138,147] (Tables 6, 8, 10). Shading requirements vary, but this species is more often associated with exposed and sunlit aquatic environments.

In northern temperate climates, *An. sinensis *females hibernate in sheltered places from the end of October, when the temperature drops to 13-15°C, to April when temperatures begin to reach 19°C [108,133]. Hibernating mosquitoes are nulliparous but mated.

### *Anopheles *(*Cellia*) *aconitus *Dönitz

*Anopheles aconitus *is a member of the Funestus Group of the Myzomyia Series [148,149]. There are three recognised members in the Aconitus Subgroup (*An. aconitus, An. pampanai *Büttiker & Beales and *An. varuna *Iyengar). All three species can be found in sympatry in mainland areas of Southeast Asia. Only *An. aconitus *has an extensive geographical range. Adults are similar to those of the Minimus Subgroup and the two taxa exhibit overlapping characters. Consequently, molecular techniques have been developed for differentiating these species [149-152].

*Anopheles aconitus *is broadly distributed throughout Indochina from southern Asia, through Southeast Asia and into the western fringe of the Australasian Region. Its range extends from Sri Lanka, southern and eastern India and southern Nepal eastward to southern China (Hainan Island and Yunnan Province), south into Indonesia as far east as Babar Island in the southern Maluku archipelago. The species is present in Bangladesh [153,154], Bhutan, Cambodia [155], China, India [156-165], the Indonesia archipelago [83,166-174], including Alor, Babar, Bali, Flores, Java, southeast Kalimantan, Kisar, Lombok, Pantar, Sulawesi, Sumatra, Sumba, Sumbawa, Timor and Timor-Leste [92], Laos [175], peninsular Malaysia [100,176,177], Myanmar [178], Nepal [127], Singapore [80], Sri Lanka [10,179], Thailand [29,180] and Vietnam [150,181,182]. Although suitable habitats exist, *An. aconitus *has not been reported in the Philippine Islands [183] or Taiwan. Three chromosomal forms (karyotypes A, B, C) have recently been described for *An. aconitus *[29,184], however little is known about their individual bionomics and epidemiological significance. Forms B and C do not appear to be distinct species, rather cytological races of the same species.

*Anopheles aconitus *can be found from sea level to upland hill zones at higher altitudes (600-800 m), but is generally restricted to below 1000 m. Depending on the season (rainfall and/or agricultural cycle), it can be a very abundant mosquito [174,185]. Larvae are frequently found in open country near foothills and forest fringes with rice fields (active and fallow), various shallow pools (rock, stream, seepage, flood) and slow moving streams [154,158] with grassy margins (Tables 7, 9). Both coastal plain and upland rice fields (young and older plants) are particularly favoured habitats [37,161,163,169,174,182,186,187], especially when plants are closer to maturity and greater than 1.5 m in height [127,163,188]. Larvae can also be found in abundance in fallow rice fields and rain-fed pools in dry fields (Tables 7, 9). Aquatic habitats are almost exclusively clear (non-polluted but sometimes turbid or slightly cloudy), stagnant or slow-flowing fresh water, mostly sun-exposed (heliophilic), and only on occasion are larvae found in small running (lotic) streams [158,174,189]. In most cases, common larval habitats contain various floating higher plants (e.g. water hyacinth) and algae [29,154,164] (Table 5). Other natural and human-made larval sites include lakes [154], swamps, marshes, flooded grassland [182], shallow ponds [154,190], ground depressions [154,182], pools in rocks, creeks and river beds [158,174,182,190,191], irrigation channels [10,174,190], fish ponds [177], roadside storm water drains, open ditches and tanks (reservoirs) with grassy margins [29] (Table 7, 9). On rare occasions this species has been found in wells, borrow pits, wheel ruts, hoof prints or small container habitats [158,191] (Table 9).

Adult mosquitoes can be found throughout the year in many localities but often show strong seasonal population peaks and periodicities that coincide with the time of rice harvest. Females are primarily zoophilic, sometimes strongly so, and although larger animals (e.g. bovids) are the commonly preferred hosts, when they are scarce, they will feed on humans as an alternative host [10,180-182] (Table 11). Females will feed on humans both inside and outside houses and in varying proportions, depending on location, generally with no strong preference reported [100,175,187]. Feeding can occur throughout the evening, typically beginning at dusk [100,176], with the majority of females feeding on humans before midnight [161,179,192,193] (Table 11). In Timor-Leste, peak feeding commonly occurred during the first hour of the evening and continued only sporadically for the remainder of the evening [92]. Variation in feeding habits has been noted by location (e.g. coastal vs upland) and season [161,187]. Some blood-fed females will rest indoors by day [156,157], but overall this species is considered strongly exophilic throughout its range [10,156,162,171,181] (Table 11). Natural outdoor adult resting places include steep, shaded stream banks, irrigation ditches and low shaded undergrowth [188]. Common human-made resting sites are found in and around animal shelters. Little is known about adult flight range and dispersal. Older literature has described movement as limited (0.5 to 1 km) whereas others have indicated this species is capable of much longer flights [188].

Throughout much of its geographical range *An. aconitus *is considered a secondary (incidental) malaria vector [80,180] and has been implicated in the transmission of Bancroftian filariasis [194]. However, under 'ideal conditions', this species can play a major role in malaria transmission, and thus its inclusion as a DVS. This species has been incriminated as a secondary, but important regional vector of malarial parasites in Thailand [195,196] and Bangladesh [153]. In Indonesia, it is considered a primary, but focal, vector throughout much of Java and areas of Sumatra, especially in locations with intense rice cultivation [185,197]. It appears to play no, or only a very minor, role as a vector in Sulawesi, Kalimantan and the Lesser Sunda island group (e.g. Bali, Lombok, etc.). In general, vectorial capacity is diminished by marked tendencies for zoophilic feeding behaviour but can be compensated by large seasonal or continuous biting densities. Even in areas where *An. aconitus *is still regarded as a primary vector (e.g. upland areas of Java and Sumatra) [174,193], its epidemiological importance appears density dependent (both mosquito and human) and is likely to be influenced by the number of cattle or buffalo present in relation to humans. During seasonal peak periods when large numbers of adults are in close proximity to more concentrated human populations, especially when fewer cattle or other non-human hosts are available, its medical importance can dramatically increase [80]. The close association of *An. aconitus *with rice cultivation practices and periodic adult population peaks has been linked to increased malaria transmission in central Java during the two main periods of harvest (March-April and August-September) [185]. In fact, in the early decades of malaria control in Indonesia, knowledge of the close relationship of this species with rice and irrigation schemes lead to the development of successful, non-chemical, vector control practices using environmental and mechanical interventions such as intermittent irrigation and drainage schemes [188].

### *Anopheles *(*Cellia*) *annularis *van der Wulp

*Anopheles annularis *is the nominotypical member of the Annularis Group in the Neocellia Series [6]. The Group currently includes five formally named species in southern Asia: *An. annularis*, *An. nivipes *(Theobald) and *An. philippinensis *Ludlow, which are widespread in the region, *An. pallidus *Theobald, which is known in Sri Lanka, India and Myanmar, and *An. schueffneri *Stanton, which occurs in Java and Sumatra. *Anopheles annularis *is widely distributed in southern Asia from Afghanistan eastward through areas of Pakistan, India, Nepal, Sri Lanka, Bangladesh, Myanmar, southern China, Taiwan, Thailand, Cambodia, Malaysia, Indonesia, Timor-Leste, Vietnam and the Philippines. It is an important vector of malaria in India, Nepal and Sri Lanka [13,198-206], but is considered to be of minor importance elsewhere [80,199]. It plays a role in malaria transmission in Myanmar [207] and has been incriminated as a vector along border areas of Thailand and Cambodia [208]. Differences observed in the vectorial capacity of *An. annularis *may be due to variation in population densities or genetic structures in different localities. The species has been reported to occur, for example, in very large numbers in Sri Lanka [204] and India [13] in association with irrigation, and was incriminated as a vector of *P. vivax *in villages with river-irrigated rice fields in Afghanistan [209]. *Anopheles annularis *is regarded as a secondary vector in Myanmar [22], but is responsible for epidemic outbreaks of malaria in the Rakhine coastal region where population densities increase dramatically after major cyclone activity [210,211]. Similarly, *An. annularis *may transmit malaria in areas where humans are the most available hosts, for example, Maheswary *et al*. [212] found a high rate of sporozoite infections in *An. annularis *during a *P. vivax *epidemic in a village in the Narayanganj District of Bangladesh where cattle were absent near houses.

Differences observed in the banding patterns of the ovarian polytene chromosomes led Atrie *et al*. [213] to conclude that *An. annularis *consists of two sibling species in India, which they provisionally designated as species A and B. More recently, Alam *et al*. [214] developed PCR-restriction-fragment length polymorphism (PCR-RFLP) assays based on endonuclease restriction sites in the ITS2 and D3 regions of rDNA which accurately distinguished the two species. However, the assays were developed using specimens collected from areas where Atrie *et al*. [213] found chromosomal forms A and B, i.e. assays were not directly correlated with cytologically identified specimens.

Species A and B are sympatric in the Shahjahanpur and Ghaziabad Districts in Uttar Pradesh, India, but only species A has been found in Assam, Haryana, Orissa and Rajasthan. Both species have been collected in non-riverine and canal-irrigated ecotypes in Shahjahanpur District. Species A has been found in similar ecotypes in districts where species B is not known to occur, and has also been found in hilly-forested areas [213]. *Anopheles annularis *is only considered to be a vector in areas of Assam and Orissa States where species B is absent. Consequently, the realisation that *An. annularis *consists of two species does not explain why it is a vector in only certain areas of India. Whether species A and B are more widely distributed in India and in other countries needs to be investigated.

Larvae of *An. annularis *are typically found in clean, still bodies of water with abundant vegetation, especially ponds, swamps and rice fields [80] (Tables 5, 7). They are strongly associated with hill rice fields in Java [83] and have been found in a wide variety of habitats in Thailand, including ponds, swamps, marshes, ditches, pits, wells, sand pools, ground pools, flood pools, stream pools, stream margins, seepage springs, rice fields, animal footprints and rock pools [29] (Tables 7, 9). Harbach *et al*. [215] collected larvae of *An. annularis *in association with larvae of *An. minimus *Theobald, *An. nivipes *(Theobald) and *An. vagus *Dönitz in a rice field pool near the Thai- Myanmar border. Females will enter human dwellings and animal shelters [80,216]. They are primarily zoophilic (e.g. Parida *et al*., [216]) but are known to bite humans in the presence of cattle [217] (Table 11).

### *Anopheles *(*Cellia*) *balabacensis *Baisas

*Anopheles balabacensis *is a member of the Leucosphyrus Complex, which is placed in the Leucosphyrus Subgroup of the Leucosphyrus Group within the Neomyzomyia Series. The complex also includes *An. leucosphyrus *Dönitz, *An*. *latens *Sallum & Peyton and *An. introlatus *Colless [28]. All but *An. introlatus *are vectors of human malaria.

*Anopheles balabacensis *inhabits forested areas of the Philippine Islands (Balabac, Culion, Palawan), Brunei, Malaysian Borneo (eastern Sarawak, Sabah) and Indonesia (East Kalimantan, South Kalimantan, Java, Lombok, Sumbawa, Sumba) [28,166,218-222]. The immature stages are principally found in shaded temporary pools of stagnant fresh water, including puddles, animal footprints, wheel tracks, ditches and rock pools (Tables 5, 9). Larvae have been collected in animal wallows in primary forest in Sabah ([223] Harbach, unpub obs). They are sometimes found at the edges of swamps, streams and rice fields, and less frequently in containers (e.g. coconut shells, cocoa pods, barrels, drums and buckets) in shaded, partially shaded or sunny locations (Tables 7, 9).

*Anopheles balabacensis *is considered the main vector of human malaria in northern and eastern areas of Borneo [193,223-228], central Java [169,229] and in the mountainous area of Lombok Island [167]. Harbach *et al*. [221] found an infection rate of 1.3% for *P. falciparum *in South Kalimantan, Indonesia based on the ELISA detection of sporozoites. On Banggi Island off the northern coast of Sabah, *An. balabacensis *was found positive for *P. falciparum *sporozoite antigen by IRMA [227]. Based on human-landing rates and sporozoite positive females, Hii *et al*. [227] calculated an entomological inoculation rate of 160 infective bites per person per year, and estimated vectorial capacity to be 1.44-19.70 in Kapitangan and 7.44-9.97 in Palau Darat (Indonesia). *Anopheles balabacensis *is considered to be a secondary vector of malaria on Palawan Island (the Philippines) [230], and Vythilingam *et al*. [11] found that *An*. (*Anopheles*) *donaldi *Reid appears to have replaced *An. balabacensis *as the main vector in the Kinabatangan area of Sabah as a result environmental changes (deforestation) and malaria control activities. In central Java, this species is closely associated with heavily forested (natural and agricultural) foothill environments and has been collected in shaded salak palm (*Salacca edulis*) plantations (Bangs, unpub obs). *Anopheles balabacensis *is also involved in the transmission of Brugian and Bancroftian lymphatic filariasis [226,231-233].

Schultz [230] found that *An. balabacensis *on Palawan Island entered houses and fed on humans principally between 20:00-03:00 h. In Sabah, *An. balabacensis *females mainly feed outdoors, with peak activity between 22:00-02:00 h, but will also feed indoors and rest outdoors afterwards [234-238]. Chiang *et al*. [239], however, observed that peak biting activity occurred shortly after midnight in three villages in Sabah. In contrast, Vythilingam *et al*. [11] found that *An. balabacensis *feed outdoors throughout the night with peak activity between 19:00 and 20:00 h, whereas indoor feeding peaked between 22:00 and 23:00 h. The biting activity of *An. balabacensis *is strongly exophagic in the mountainous area of Lombok Island, Indonesia where biting activity was highest from 19:00-21:00 h and gradually decreased toward morning [167] (Table 11).

### *Anopheles *(*Cellia*) *culicifacies *Giles species complex (Culicifacies Complex)

*Anopheles culicifacies *is a complex of species within the Funestus Group of the Myzomyia Series [6]. The Culicifacies Complex includes five isomorphic species informally designated species A, B, C, D and E. The members of the complex have been cytogenetically separated and exhibit biological differences in their behaviour, seasonal prevalence, distribution and vectorial capacity [240,241]. More recently, molecular assays have been developed, including PCR-RFLP assays [242,243], allele-specific PCR assays [244,245], and a multiplex PCR [246]. However, no single currently available application can directly identify all five species, indicating that the techniques are weak, raising some doubt about their validity. More sequences need to be analysed in order to identify those that show more clear species differences (Manguin, unpub obs).

The Culicifacies Complex is widely distributed across Southeast Asia, including southern China, India, Pakistan, southern Afghanistan and Iran, with a western extension into the Arabian Peninsula (Yemen) and Ethiopia [99,209,247-250]. The bionomics and ecology of the species within this complex have been largely studied in India [23,241,251] and Sri Lanka [99,252,253], but there is a general lack of detailed information from other regions, especially the western areas [8]. Four species of the complex (A, C, D, E) are reportedly malaria vectors in India where they are apparently responsible for transmitting 60-65% of all cases of malaria in peri-urban and urban environments [254]. *Anopheles culicifacies *E, due to its high anthropophilic and endophilic behaviour [255,256], is the most important and efficient vector of *P. falciparum *and *P. vivax *in southern India and Sri Lanka. Species A, C and D appear to be mainly zoophilic with very low anthropophilic indices of 3-4% [251] (Table 11). Therefore, these three species generally play very minor roles in malaria transmission compared to species E [257]; however, species C was found to be responsible for local malaria transmission in deforested riverine areas of central India [258]. Due to its highly zoophilic behaviour, species B is considered to be a poor or non-vector [241,259] (Table 11). This species has the widest distribution of all members of the complex, occurring from Iran and Sri Lanka to Southeast Asia, and is the only species of the complex found in the far eastern areas of southern China (Sichuan), Vietnam, Laos, Cambodia and northwestern Thailand [180,247-250,253,260]. Species B occurs in sympatry with other species of the complex, particularly species E, in western areas of its distribution. Sympatric populations of two or more sibling species are also common in India [254].

Species of the Culicifacies Complex are abundant in plains, hilly and mountainous areas up to elevations of 1500 to 2000 m in Afghanistan (Kabul region) and the Indian Himalayas [23,261]. They occur in different ecotypes, ranging from forested areas with perennial streams to deforested riverine ecosystems and irrigated areas. Larval habitats include irrigated canals, stream margins, seepages, borrow pits, hoof marks, rock pools, sandy pools near rice fields, rock quarries, newly dug pits, ponds, domestic wells, tanks and gutters [23,249,252,254,262-264] (Tables 7, 9). Immature stages develop in fresh-water habitats but tolerance to moderate salinity has been reported in Oman where larvae have been collected in concrete reservoir tanks containing brackish water [265] (Table 5). Similarly, species E is able to tolerate variable salinity due to monsoonal rain in Sri Lanka [252] where it otherwise exploits a wide range of aquatic habitats, reflecting the significant environmental adaptability of this malaria vector [264]. Nanda *et al*. [258] studied the presence of species A, B and C in forested and deforested ecosystems in Orissa (India) and found that *An. culicifacies *C (71%) greatly outnumbered species B in forested areas, whereas species C (78%), B (21%) and A (1%) were present in quite different proportions in deforested areas. These data also reflect the ability of *An. culicifacies *C to inhabit different ecosystems. In India, species A has been shown to be more abundant in villages with domestic wells, whereas species B was found in higher densities in villages with streams [23]. Studies have shown that adult biting activity occurs during the first half of the night in cooler months (November-March) and during the second and third quarters of the evening in the warmer months (September-October), whereas others reported peak biting activity occurring around 23:00 h to midnight [266,267]. Post-feeding behaviour of the species showed a higher tendency for resting indoors, mainly in cattle sheds, but outdoor resting has also been reported [268,269]. As members of the Culicifacies Complex exhibit distinctly different vectorial capacities and behaviour, a more thorough study of the bionomics of each species must be undertaken to specifically and efficiently target control efforts against those species involved in malaria transmission.

### *Anopheles *(*Cellia*) *dirus *Peyton & Harrison species complex (Dirus Complex)

Species of the Dirus Complex are closely related to members of the Leucosphyrus Complex, and this has been the cause of considerable confusion in the published literature [8]. Numerous studies, mainly based on crossing experiments, cytogenetics, allozyme data and more recently molecular methods, have been necessary to recognise the individual species and to confirm their taxonomic status [28,220,270-274].

Members of the Dirus Complex inhabit forested mountains and foothills, cultivated forests, plantations (e.g. rubber) and forest fringes. As Rosenberg *et al*. [275] stated, "The danger from *An*. *dirus s.l*. is not only that it is very resistant to control within its habitat but that it is an extraordinarily efficient vector, so long-lived and anthropophilic that only a small population is necessary to maintain high malaria endemicity". The situation is, however, more complicated as the Dirus Complex includes seven species that vary from highly competent vectors of malaria and Bancroftian filariasis to non-vectors. Each member of the complex has now been formally named: *An*. *dirus *(formerly *An. dirus *species A), *An*. *cracens *Sallum & Peyton (formerly sp. B), *An*. *scanloni *Sallum & Peyton (formerly sp. C), *An*. *baimaii *Sallum & Peyton (formerly sp. D), *An*. *elegans *(James) (formerly sp. E), *An*. *nemophilous *Peyton & Ramalingam (formerly sp. F) and *An. takasagoensis *Morishita [218,274,276,277]. The primary disease vectors are *An. dirus *and *An. baimaii*, which transmit *P. falciparum *and *P. vivax*, as well as *Wuchereria bancrofti *[7,19,278-281]. Both species are highly anthropophilic, exophagic as well as endophagic and exophilic [181,271,278,280,282-284] (Table 11). Studies have shown that biting activity is species-specific, for example in Thailand, *An. dirus *has a tendency to bite between 20:00 and 23:00 h and *An. baimaii *from 22:00 h to 02:00 h [28,274,285], although in India earlier biting at 20:00-21:00 h was also recorded for *An. baimaii *[278] (Table 11). *Anopheles scanloni *is also anthropophilic and plays a more focal role in malaria transmission of both *P. falciparum *and *P. vivax *in Thailand [286]. This is an early evening biter with peak activity starting at dusk, between 18:00-19:00 h [285]. There is no clear evidence that *An. cracens *(restricted to the Thai-Malaysian peninsular) and *An. elegans *(only present in hill forests of southwestern India) are involved in malaria transmission [274,286].

The recent development of two allele-specific PCR assays that identify sympatric species, such as *An. dirus*, *An. cracens*, *An. scanloni*, *An. baimaii *and *An. nemophilous *[287,288], will allow for more precise determination of the degree by which each species may be involved in malaria transmission. The two remaining species of the complex, *An. nemophilous *and *An. takasagoensis*, the latter species being restricted to Taiwan, appear to be non-vectors of human malaria due to their strict zoophilic behaviour [271,276].

Larvae of the Dirus Complex typically inhabit small, shallow, usually temporary, mostly shaded bodies of fresh, stagnant (or very slowly flowing) water, such as pools, puddles, small pits (e.g. gem pits), animal footprints (e.g. elephant footprints), wheel ruts, hollow logs, streams and even wells located in primary, secondary evergreen or deciduous forests, bamboo forests and fruit or rubber plantations [7,271,289-292] (Tables 5, 9). Water can be clear or turbid [293], and habitats with nitrogenous wastes, due to elephant and buffalo excreta or rotten leaves, appear more productive [291] (Table 5). These species are most abundant during the rainy (monsoon) season due to the larval requirement and oviposition preference for small temporary pools [271,284,293,294].

### *Anopheles *(*Cellia*) *farauti *Laveran species complex (Farauti Complex)

See *Anopheles *(*Cellia*) *punctulatus *Dönitz species group below.

### *Anopheles *(*Cellia*) *flavirostris *(Ludlow)

*Anopheles flavirostris *is a member of the Minimus Subgroup within the Myzomyia Series [295]. For many decades, this species was regarded as a subspecies of *An. minimus*, but once elevated, its species status has been supported by most authorities [119,180]. Somboon *et al*. [296] presented conclusive evidence supporting specific status using hybridisation experiments and internal morphology (cibarial armature). Molecular studies of rDNA have further substantiated *An. flavirostris *as a valid species [295,297,298]. All previous records of *An. minimus *from the Philippines, Sabah and Indonesia are now considered invalid with only *An. flavirostris *regarded as present in these areas.

*Anopheles flavirostris *occurs extensively throughout the Philippines, through much of Indonesia, in eastern Malaysia (Sabah, Borneo) [19,80,296,298] and Timor-Leste [92]. In Indonesia, it has been reported on the larger islands of Sumatra, Java, Kalimantan and Sulawesi, and is scattered across the smaller islands of the Lesser Sunda island group, extending as far east as Timor [173]. Records from smaller islands include Bali, Lombok, Sumbawa, Sumba, Flores and western Timor [166-168,170,296,298,299] (Bangs, unpub obs). Older records of its presence in the Maluku island group (Seram) have not been verified. Interestingly, species distribution on the island of Borneo (Kalimantan) appears confined to the eastern side of the island and nearer the coast. It has not been reported from northwestern (Sarawak, Malaysia) or western Kalimantan. It occurs in sympatry with three other species of the Minimus Group: *An. aconitus *(Indonesia), *An. filipinae *Manalang and *An. mangyanus *(Banks) (the Philippines). The absence of a pale fringe spot at vein 1A on the hind margin of the wing distinguishes this species from *An. aconitus *and *An. filipinae*. The species differs from *An. mangyanus *(an incidental malaria vector in the Philippines) in usually lacking a presector pale spot on the costa or the absence of pale scales basal to the sector pale spot.

Epidemiologically, this species has been incriminated frequently as a vector of human malarial parasites in the Philippines [300,301] and is regarded as the primary vector throughout much of the country [302]. Despite normally low numbers of sporozoite-infected mosquitoes detected, under favourable circumstances low infective rates remain sufficient to maintain endemic transmission or cause outbreaks [302]. It has been implicated in malaria transmission above 1000 m elevation in Luzon (Villanueva & Kalaw *in *[302]). It has also been incriminated as a vector of *W. bancrofti *on Luzon and Palawan Islands in the Philippines [80]. It is a confirmed malarial vector in Sabah (Malaysian Borneo) along the eastern coast (Banggi Island, Semporna, Pitas) [223] (Bangs, unpub obs). Only a few historical records of natural infections are known from Indonesia [185,188], specifically in western Java, Sulawesi and Palau Laut in southeastern Kalimantan (Borneo). In Indonesia, this species is seldom encountered in human-landing collections and is regarded as only an incidental, focal vector.

*Anopheles flavirostris *is quintessentially a 'foothill', stream-breeding species but is by no means entirely restricted to such lotic environments (Table 5). In the Philippines, *An. flavirostris *can be found from the coastal plains near sea level to elevations up to 1500 m [302], although it is more commonly found no higher than 600 m elevation throughout its range [119,167,188].

Given its importance as a malaria vector, the majority of bionomic information on this species has come from the Philippines [302,303]. Characteristically, this species has a high preference for clear, slow-moving fresh-water habitats that are typically partly shaded by surrounding overhead vegetation and with margins containing emergent plants or grasses [304] (Table 5). In the foothills of western Java, it has been commonly collected from margins of forested streams with moderate to high flow rates [174]. It can also be found at the edges of seepage pools, slow-flowing, grassy river edges, canals and irrigation ditches. It has been reported from natural wells and occasionally stagnant pools, and very rarely from rice fields or ponds [188] and pools in stream beds [305] (Tables 7, 9). It typically has a low tolerance for salinity [119] and prefers more alkaline (7.3-8.2) water [302]. Foley *et al*. [306] also reported that most larvae occurred in areas dominated by overhanging vegetation other than grass. They also found early instar larvae were more likely to be present in heavily shaded sites, suggesting sunnier areas were less preferred as oviposition sites, whereas overall larval abundance was higher in less shady locations. Late instars were more evenly dispersed and their presence only weakly related to available shaded conditions (Table 5). Larval habitats have been described as being relatively close to human habitation compared to many other species [174,306]. In western Java, Stoops *et al*. [174] examined environmental determinants of spatial distribution for *Anopheles *and found *An. flavirostris *associated with lower elevation foothill sites, lower water temperatures with less acidity, greater water depth, higher water current, rocky substrate, higher canopy cover, greater forested riparian vegetation and higher amounts of low emergent vegetation compared to most other anopheline species in the area.

Adult females are primarily zoophilic, preferring to feed on larger animals (e.g., water buffalo, cows), although they will readily attack humans both indoors and outdoors [167,301,305,307]. Hii *et al*. [227,308] described this species as primarily human-biting and endophagic in Sabah. It is regarded as exophagic, but varies depending on the circumstances and season [307]. This species may be opportunistic in feeding habits and can show a varying preference for biting location that appears dependent on the availability of hosts. Near equal biting proportions between indoors and outdoors have also been reported [187,227,230,301,305,307]. Females blood-feed throughout the evening with lower numbers in the early evening gradually increasing to peak biting frequency on humans nearer midnight and for several hours afterwards (22:00-03:00 h), with a sharp drop off in activity before dawn [187,230,305,307,308] (Table 11).

Females are strongly exophilic, resting during the day on low vegetation, often near cool, damp overhanging stream banks close to larval habitats [302]. Very seldom are they found resting indoors during daylight hours, although pre- and post-feeding indoor resting does occur [227,309], but rarely for long periods before exiting the house (Table 11). The flight range is considered short, at a maximum of 1-2 km from origin (Russell & Santiago *in *[302]).

### *Anopheles *(*Cellia*) *fluviatilis *James species complex (Fluviatilis Complex)

*Anopheles fluviatilis *is a complex of species within the Funestus Group of the Myzomyia Series [6]. Members of the complex are widely distributed in forested hills and mountainous regions of southwestern Asia, including Iran, Pakistan, Afghanistan, India, Nepal, Bangladesh and Myanmar [160,161,249,258,283,310]. However, little detailed information is available on the bionomics, ecology and distribution of the species outside of India and Iran [8]. The complex includes three sibling species, informally designated S, T and U, based on cytogenetic differences [310], and a form V of uncertain status [311]. Molecular techniques have been developed to distinguish the three sibling species [312-314]. Species T has the widest distribution, which includes India, Nepal, Pakistan and Iran [311]. Species U has been recorded in northern India and Iran, and species S appears to be restricted to India [311,315]. *Anopheles fluviatilis *S is the most anthropophilic and endophilic species of the complex [258], and is regarded as a highly efficient malaria vector in hilly regions of India [257,316]. *Anopheles fluviatilis *T and U are primarily zoophilic, exophagic and exophilic, and are considered to be poor or non-vectors in India [316] (Table 11). However, in Pakistan, Nepal and Iran, species T has been recorded as an important malaria vector in general, or a localised vector for maintaining malaria in mountainous and hilly regions [199,317,318]. Biting activity begins around 19:00 h and peaks between 20:00 h and 21:00 h, but may also occur throughout the night until dawn without an apparent peak [161] (Table 11). A study conducted in Orissa State of India showed that members of the complex are essentially absent in deforested areas, but *An. fluviatilis *S is the predominant species in forested areas (98% species S; 2% species T) [258].

Larvae of *An. fluviatilis *are generally associated with slow-flowing streams or river margins, in direct or diffuse sunlight. They have also been reported from rice fields, often in low numbers, possibly washed into the fields from the irrigation channels where they tend to be found in higher densities [158,163,191,294,319-324] (Tables 5, 7, 9)

### *Anopheles *(*Cellia*) *koliensis *Owen

See *Anopheles *(*Cellia*) *punctulatus *Dönitz species group below.

### *Anopheles *(*Cellia*) *leucosphyrus *Dönitz and *An*. (*Cel*.) *latens *Sallum & Peyton

*Anopheles leucosphyrus *and *An. latens *are members of the Leucosphyrus Complex, which is placed in the Leucosphyrus Subgroup of the Leucosphyrus Group within the Neomyzomyia Series. The subgroup also includes *An. balabacensis *Baisas and *An. introlatus *Colless [28]. All but *An. introlatus *are competent vectors of human malaria.

*Anopheles leucosphyrus *and *An. latens *are sister species [325]. The former is found in forested areas of Sumatra and the latter in forested areas from the extreme south of Thailand through peninsular Malaysia, and northern and eastern areas of Borneo (excluding Sabah except its western border area with Sarawak) [274]. Both species are important vectors of malaria in areas where they occur. *Anopheles leucosphyrus *and *An. latens *were regarded as the same species (*An. leucosphyrus*) until Baimai *et al*. [326] provided evidence from mitotic karyotypes and cross-mating studies that they were separate species. Most of the published literature on "*An. leucosphyrus*" refers to *An. latens*, and little information pertains to the genetic species in Sumatra that is now known as *An. leucosphyrus*.

Females of *An. leucosphyrus *are attracted to humans inside and outside houses situated at the edge of forest [274,326] (Table 12). This species has been shown to be a vector of human malaria in Sumatra ([80] and in older literature cited by Sallum *et al*. [274]). Little else is known about its bionomics but it is presumably similar to other members in the Leucosphyrus Group.

*Anopheles latens *is a primary vector of human malaria in forested areas and villages near forests in Sarawak [327-330]. Females bite throughout the night, but peak activity occurs at different times in different locations. de Zulueta [328] found that biting females were more abundant between 24:00 h and 02:00 h in mountainous areas, Colless [327] observed peaks of activity from 22:00-04:00 h during the dry season and from 22:00-24:00 h in the wet season in the Akah River region, and Chang *et al*. [329] recorded peak activity around midnight in forested areas and soon after dusk in village settlements in the Baram District. Adults do not rest in houses by day, but will enter to bite at night, mostly after 22:00 h [327] (Table 12). Macdonald & Traub [331] and Wharton *et al*. [332] noted that *An. latens *was collected more frequently in the canopy than at ground level in lowland secondary dipterocarp forests in peninsular Malaysia. The species also occurs in environments that have been altered by human activities, for example, areas of secondary forest with fruit and rubber plantations [274].

In Sarawak, Chang *et al*. [329,330] found *An. latens *more abundant and malaria transmission more intense at farms located in forest fringe areas than in village settlements further removed from forest. The entomological inoculation rate for *An. latens *was calculated at 0.11 infective bites per person per night in a village perimeter site and 0.15 in a forested area in the Baram District [329]. In contrast, Chang *et al*. [330] estimated the inoculation rate in farm huts in the Belaga District to be 0.023 [330]. Harbach *et al*. [221] recorded a sporozoite infection rate of 1.0% in a remote village in South Kalimantan, Indonesia, where *An. latens *females attacked humans in higher numbers than in nearby forest.

In addition to human malarial parasites, *An. latens *is also known to transmit the monkey malarial parasite *Plasmodium knowlesi *to humans in the Kapit District of Sarawak [333]. Tan *et al*. [334] found that *An. latens *is the main vector of *P. knowlesi *in dense jungle and forest fringes in the district. Nearly 90% of females attracted to humans were collected in forest (50%) and at a farm located amid fruit trees and secondary vegetation (40%). In contrast, only 10% were collected in a longhouse (traditional home of indigenous people) surrounded by trees and shrubs near a river, and of these, 71% were collected outdoors. The inoculation rates of *P. knowlesi *by *An. latens *in the forest and farm were estimated at 4.6 and 7.8 infective bites per person per year, respectively.

Like other members of the Leucosphyrus Group, larval habitats of *An. latens *are mostly shaded temporary pools and natural containers of clear or turbid water on the ground in forest areas (Table 6). Wharton [335] noted that larvae of *An*. *latens *were usually found in clear seepage pools in forest swamps in peninsular Malaysia. In Sarawak, Colless [327] found larvae in pools beside a forest stream and in swampy patches in hilly country. Habitats occupied by *An. latens *in Thailand include stump ground holes, sand pools, ground pools, flood pools, rock pools, stream pools, stream margins, seepage-springs, wheel tracks and elephant footprints [29,274] (Table 10).

### *Anopheles *(*Cellia*) *maculatus *Theobald species group (Maculatus Group)

*Anopheles maculatus *belongs to the Maculatus Subgroup within the Maculatus Group of the Neocellia Series. In addition to *An. maculatus*, the group includes eight other formally named species [6,336]: *An. dravidicus *Christophers (the second member of the Maculatus Subgroup); *An*. *notanandai *Rattanarithikul & Green, *An. rampae *Harbach & Somboon (formerly *An. maculatus *species K; see Somboon *et al*. [336]) and *An. sawadwongporni *Rattanarithikul & Green, which belong to the Sawadwongporni Subgroup, and four species, *An. dispar *Rattanarithikul & Harbach, *An. greeni *Rattanarithikul & Harbach, *An. pseudowillmori *(Theobald) and *An. willmori *(James), which are unplaced within the group [337]. Members of the group have a varied distribution from Afghanistan and Pakistan to southern China, Indonesia and the Philippines. Two species, *An. dispar *and *An. greeni*, are found exclusively in the Philippines [338,339] and can be identified using a PCR-RFLP assay [339]. The application of this molecular method may help shed light on the vector status of these two species as data collected on previously undifferentiated *An*. *maculatus s.l*. in the Philippines are considered unreliable. In addition, two allele-specific PCR assays have been developed to distinguish *An. dravidicus*, *An. maculatus*, *An*. *pseudowillmori*, *An. sawadwongporni *and either *An. willmori *[337] or *An. rampae *[340]. These species are variously involved in malaria transmission [8,341-343]. However, again, the vector role of each species is not precisely known due to previous misidentifications based solely on overlapping morphological characters. Further uncertainty arises within this group as the vectorial capacity of a species appears to vary depending on geographical location. In general, females are more strongly attracted to cattle than humans, but freely bite people both inside and outside houses (Table 12). *Anopheles maculatus *and *An*. *sawadwongporni *appear to be the least zoophilic of the species and exhibit early biting activity, peaking between 18:00 h and 21:00 h [155,182,215]. Even though *An. maculatus *has the widest distribution of all species of the group, it is only an important vector of human malarial parasites in hilly areas of eastern India, southern Thailand, peninsular Malaysia and south-central Java [169,196,281,341,344]. *Anopheles sawadwongporni *has been found with malaria sporozoite rates of 1-2% in Thailand where it is considered an important vector [345,346]. *Anopheles pseudowillmori *is a secondary vector in northwestern Thailand along the Myanmar border [196,272]. *Anopheles willmori *is one of the primary vectors in Nepal [347], but it is seldom collected in Thailand and does not appear to be involved in malaria transmission there. *Anopheles dispar *and *An. greeni*, regarded as secondary vectors in the Philippines, exhibit strong exophagic and zoophilic behaviours, with a biting rate on water buffalo that is 50 times greater than on humans [305]. *Anopheles notanandai*, *An. dravidicus *and *An. rampae *are not known to be involved in malaria transmission [33,336].

Members of the Maculatus Group are typically found in or near hilly and mountainous areas. Larvae have been collected in a diverse number of permanent or semi-permanent bodies of clean water that are often exposed to direct sunlight, including ponds, lakes, swamps, ditches, wells, different types of pools (grassy, sandy, ground, flood, stream), margins along small, slow-flowing streams, gravel pits along stream margins, seepages, springs, rice fields, foot and wheel prints, and occasionally tree holes and bamboo stumps [29,189,215,348-351] (Tables 6, 8, 10). More specific studies have shown that each species has a preferred habitat. For instance, larvae of *An. willmori *are found only along stream margins at altitudes between 990 and 1450 m in northern Thailand, whereas larvae of *An. pseudowillmori *have been collected primarily in rice fields, stream margins, ponds, pits and wells [29,351]. *Anopheles maculatus *prefers to use pools of water formed on the banks of rivers and waterfalls. The most common larval habitats are shallow pools 5-15 cm deep with clear water, mud substrate and emergent plants. This species also requires, or strongly prefers, open to partially shaded habitats. Habitats are commonly located at 100-400 m from the nearest human settlement [352] (Table 6). The combination of the early evening biting activity of these malaria vectors (particularly *An*. *maculatus *and *An*. *sawadwongporni*) and their zoophilic and exophilic tendencies indicates that they will be less affected by vector control methods based on IRS and ITNs. However, a strategy of creating a barrier using insecticide on vegetation near cattle or other animal hosts may prove significant in the control of these vectors [182].

### *Anopheles *(*Cellia*) *minimus *Theobald species complex (Minimus Complex)

The Minimus Complex belongs to the Minimus Subgroup within the Funestus Group of the Myzomyia Series. *Anopheles minimus s.l*. is considered a primary malaria vector taxon in the hilly forested regions of mainland Southeast Asia. *Anopheles minimus s.l*. comprises three sibling species, namely *An. minimus *(formerly species A), *An. harrisoni *Harbach & Manguin (formerly sp. C) and *An. yaeyamaensis *Somboon & Harbach (formerly sp. E) [353-355]. Whereas *An. minimus *and *An. harrisoni *have a broad distribution in much of Southeast Asia [8], *An. yaeyamaensis *is restricted to the Ryukyu Archipelago in southern Japan where it played a major role as a disease vector until 1962 when malaria was eradicated [356,357]. Despite historical records of *An. minimus *in Indonesia, all are considered invalid and now regarded as *An. flavirostris*. The two other species, *An. minimus *and *An. harrisoni*, are vectors of malaria parasites throughout their respective distributions, although further investigation needs to be conducted on *An. harrisoni *as its implication in malaria transmission appears weaker than that of *An. minimus *[24]. *Anopheles minimus *is also involved in the transmission of *W. bancrofti *in southern China [358] and most likely in Thailand, as demonstrated under laboratory conditions by Pothikasikorn *et al*. [359]. Larvae are generally found in small to moderate-sized streams or canals with slow-running, clear and cool water, partially shaded and with grassy margins where females prefer to lay their eggs [180,189,351,360] (Tables 6, 10). They develop in various pools (rock, ground, stream and seepage) [180,221] (Table 10). Unusual larval habitats for *An. minimus *(e.g. rain water tanks) have also been reported in the suburbs of Hanoi, Vietnam [361]. *Anopheles minimus s.l*. is commonly found at elevations ranging from 200 to 900 m and is rare at altitudes above 1500 m [22,180,362]. In northern Vietnam and western Thailand, *An*. *minimus *occupies a greater variety of habitats, ranging from dense canopy forest to open agricultural fields, particularly traditional rice agro-ecosystems (Table 8). *Anopheles harrisoni *has a narrower habitat preference, being more closely linked to recently altered agro-ecosystems (e.g. maize cultivation) in deforested areas [24,34]. These differences in habitat choice may explain the wider distribution of *An. minimus *in Southeast Asia, although *An. minimus *and *An. harrisoni *are found sympatrically in several regions, including southern China, northern and central Vietnam [38], northern Laos, western and northern Thailand (Somboon, pers comm) and central and eastern Myanmar [8]. *Anopheles minimus *is the only species of the Minimus Complex found in Cambodia and northwestern India, as well as other regions of Southeast Asia not mentioned above [24,65,363]. In contrast, only in the central part of China (up to 32.5°N latitude) can *An. harrisoni *be found in the absence of *An. minimus *[358].

The adult behaviour of *An. minimus s.l*. is reported as highly diverse for two main reasons: (1) most studies do not differentiate *An. minimus *and *An. harrisoni *and (2) these two species are highly opportunistic in their habits, exhibiting considerable behavioural and ecological plasticity [24]. Females of *An. minimus *mainly bite humans (up to 93% in Assam, India), but the degree of anthropophily/zoophily depends on the availability of alternative hosts (e.g. cattle) [181,364,365]. This species is mainly endophagic in India, Thailand and central Vietnam, and more exophagic in Cambodia and northern Vietnam [181,360,366] (Table 12). Studies showed that housing in central Vietnam, made with incomplete walls of split bamboo and very large eaves, allows easy entry of the mosquito which would otherwise show exophagic behaviour [181]. Its resting behaviour is reported as exophilic in southern China, Thailand and Vietnam, and mainly endophilic in India [181,360,366]. However, the degree of endophagy and endophily of *An*. *minimus *is also largely influenced by the use of IRS, provoking either a modified behavioural response [367] or a drastic reduction in population density [368]. In contrast, the few studies conducted on *An. harrisoni *have shown a greater tendency for exophagy, exophily and zoophily and thus its role in malaria transmission is more questionable [181,369,370], despite it being reported as a main vector in China [358]. *Anopheles harrisoni *exhibits two peaks of biting activity in western Thailand, the first in the early evening, between 18:00-21:00 h, with a second, smaller peak from midnight to 02:00 h or from 03:00-06:00 h [364,369]. The early evening peak (before 22:00 h) has also been observed in northern Vietnam [181]. *Anopheles minimus *tends to bite later, with peak activity occurring around 22:00 h in Cambodia and Thailand, after 22:00 h in Vietnam and between 01:00-04:00 h in Assam, India [181,215,360] (Table 12).

Clearly more studies are required on *An. minimus *and *An. harrisoni *across a wider geographical area as many uncertainties exist in relation to their respective habitats, behaviour, involvement in malaria transmission and geographic distribution. These studies will need to utilise molecular assays to distinguish the sibling species, as well as related sympatric species such as *An. aconitus*, *An. pampanai *Büttiker & Beales and *An. varuna *Iyengar [151,152]. Moreover, the many older records of *An. minimus *from the Indonesian archipelago require confirmation, either based on adults with associated larval and pupal exuviae or, preferably, DNA analysis (Harbach, unpub obs).

### *Anopheles *(*Cellia*) *punctulatus *Dönitz species group (Punctulatus Group)

The Punctulatus Group is comprised of at least 12 sibling species which collectively span most tropical areas of the Australasian region [21,371]. Some members in the group are major vectors of malaria, and in many areas they also transmit the nocturnal periodic form of Bancroftian filariasis (*W. bancrofti*) [372-387]. The major malaria vectors include *An. punctulatus*, *An. koliensis *Owen, *An. farauti *Laveran, *An. hinesorum *Schmidt and *An. farauti *No. 4 [388-392] (Bangs, unpub data). The important characteristics these species share include an ability to occur in high densities, a predilection to feed on humans (Tables 11, 12) and a high vectorial competence (i.e. ability to develop human malaria parasites). Most of the information available on vector incrimination is based on spatially and temporally limited studies from a small number of localities, predominately in PNG.

The species group extends from the far eastern regions of Indonesia (Maluku island group and Papua), PNG (including the Bismarck Archipelago) and into the southwestern Pacific to the limits of all anopheline species distributions (Solomon Islands and Vanuatu) [393]. The group occupies a variety of different habitats, predominately in the lowlands, but extending from the coastal zone to elevations as high as 2250 m above sea level. Only recently has the taxonomy and phylogeny of this group become better defined using DNA-based molecular methods to overcome the inherent problems of accurately identifying both allopatric and sympatric populations morphologically (because of identical or overlapping characters) and those that comprise a complex of near-identical cryptic species (*Anopheles farauti s.l*.) [393-396]. The geographical isolation of numerous insular populations, and in some cases populations separated by significant physical barriers, has lead to the genetic divergence, speciation and radiation in the group. By far, the centre of evolution for this group has taken place on the island of New Guinea [31]. This has resulted in some species showing niche-specific habitat preferences whereas others show a much wider selection and diversity in habitats and behaviour. However, differences in biology and behaviour among the members of the Punctulatus Group do not appear to be reliable characters for determining phylogenetic relationships, and even closely related sympatric species (e.g. small rDNA genetic distances) can show very dissimilar bionomics [371,397].

Across the range of species, oviposition sites appear variable [31] and often dependent on seasonal availability. Generally, most species utilise earthen-bound (often non-porous, clay-like substrates) collections of fresh water that are exposed to direct sunlight either entirely or partially. Water sources shaded by thick jungle are unfavourable. Only a few members of the Farauti Complex show salt-tolerance, but brackish water is not obligatory for oviposition [30,371,398]. Ideally, water is stagnant, clear to muddy (turbid), but never heavily polluted [399] (Tables 5, 6). For those species that have been studied in more detail (*An. punctulatus*, *An. koliensis *and *An. farauti s.l*.), most show a high degree of synanthropy, and although females appear to be opportunistic blood-feeders that attack a wide range of hosts, they often have a stronger preference for humans. Other animals (pigs, dogs, cats, cattle, goats, fowl) can serve as alternative or primary blood sources, depending on locality [384]. In general, the primary vector species of the group are more exophilic in resting habits and facultatively exo- and endophagic in host blood feeding (Tables 11, 12). Outside resting sites are largely unknown or otherwise poorly described. Blood-feeding activity is predominately nocturnal whereas frequency and peak activity appears variable by locality, prevailing environmental conditions, season, time and investigative methods used for study [377,384,400-402]. Other studies suggest physiological condition (e.g. age, parity) and malaria infection can influence biting frequency and time [401,403]. Larval habitats of all vector species of the group are generally found in close proximity with human habitation.

The relatively recent understanding of the inter-specific morphological variation and genetic diversity in this taxon casts doubt on past data on mosquito behaviour, larval habitat preferences [377,384,404] and on the interpretation of those studies conducted before the advent and common use of biochemical and molecular methods to accurately identify species [371,389,391,394,396,405-407]. Moreover, intra-species heterogeneity in both bionomics and behaviour over each species' range further complicates interpretation of the data collected before the existence of sibling species within the group was fully recognised and appreciated. Variations in study designs also undoubtedly contribute to the apparent heterogeneity, and sometimes, conflicting observations. More recently, studies have begun to identify specific differences in ecology and behaviour (e.g., larval habitats, biting cycles and host preferences) of some of the sibling species using molecular-based (primarily nuclear and mitochondrial DNA) identification techniques [391,394,408,409]. However, much more work is needed on the group to better understand the bionomics, respective role and epidemiological contribution of each species/morphotype in malaria and filarial transmission and to improve vector control strategies.

In general, there is more published work describing species distribution and adult behaviour in relation to disease transmission and control but far less on larval biology and habitat characteristics [31,371,410,411]. Moreover, compared to PNG, northern Australia and the malaria endemic island groups in the southwestern Pacific, there is a significant paucity of information on this species group from Papua (western half of New Guinea Island) and the Maluku Archipelago in eastern Indonesia.

### *Anopheles *(*Cellia*) *punctulatus *Dönitz species complex (Punctulatus Complex)

*Anopheles punctulatus *is the nominotypical member of the Punctulatus Group of the Neomyzomyia Series. *Anopheles punctulatus s.l*. comprises two apparent species, *An. punctulatus *and *An*. sp. near *punctulatus *[393]. *Anopheles *sp. near *punctulatus *is relatively uncommon and has only been found in a few remote highland localities on the island of New Guinea (Papua, Indonesia and PNG), and very little is known about its biology or role in disease transmission [31,392,412,413]. *Anopheles punctulatus*, however, is highly susceptible to infection by *Plasmodium *parasites and is an efficient and important vector of human malaria in many areas throughout its range. This species is also a vector of periodic Bancroftian filariasis in New Guinea and Guadalcanal [374,378,386,387]. It can be found in lowland river valleys and plains with extensions up to elevations above 1700 m, possibly extending above 2000 m on occasion [31]. Its distribution appears to be very limited west of New Guinea Island (excluding Halmahera Island in northern Maluku, its presence remains questionable throughout the rest of the island chain) and is apparently of less epidemiological importance moving eastward of the PNG mainland into the Bismarck Archipelago and the Solomon Islands chain [371,390].

The immature stages of *An. punctulatus *prefer small, scattered, shallow, sunlit (although partial shade is tolerated) temporary pools of fresh water (Tables 6, 10). Oviposition has been observed on muddy pools and even moist soil [399]. Water can be clear or turbid (muddy), but never brackish. In some cases, high organic content (e.g. animal excrement, typically pigs) is tolerated (Bangs, unpub data) (Table 6). Most transient sites are maintained by rainfall or are found in areas with high water tables. Other natural sites include sand or gravel ground pools in small streams and riverbeds, and larvae are only occasionally found in rock pools [31] (Table 10). Often sites are completely free of natural predators with little or no vegetation (occasionally algae or sparse emergent plants). This species will generally only thrive in areas with perennial rainfall, however eggs can withstand desiccation and larvae have the ability to survive in damp mud in the absence of free water for several days during limited periods of drought [399]. Often man-made small ground depressions or those created by animals (rooting pigs, hoof prints) become ideal habitats, as well as recently disturbed areas such as land clearing for gardens and road construction, and natural landslides. Larvae are able to withstand water temperatures exceeding 40°C and typically have rapid growth and a short development period to adults (5-9 days). High densities of larvae and general abiotic conditions of recently created sites can result in significant cannibalism as a survival mechanism. Not infrequently, this species is found in habitats with *An. farauti s.l*. and *An. koliensis*..

*Anopheles punctulatus *is particularly effective at exploiting disturbed ecology [414]. Populations may reach high densities in very short periods of time when environmental and seasonal conditions are favourable. Under optimal conditions with rapid, synchronous larval development, this species can quickly invade recently disturbed (cleared) and previously uncolonised areas to produce large numbers of adults. The efficiency by which this species can quickly exploit sudden and dramatic changes in habitat (e.g. temporary pools formed by recession of rivers in drought conditions) has resulted in severe and unexpected outbreaks of malaria in the highlands of New Guinea [415].

Adults are often found in close proximity to human habitation and larval habitats, with females readily attacking humans outdoors but also entering houses in search of hosts. Individuals that feed in houses may rest indoors for the entire evening and daytime but the vast majority of females leave before dawn to rest outdoors [399,416,417]. Feeding frequency and peak activity is variable by locality, environmental conditions and season with peak activity occurring around or after midnight in some areas, and the majority of biting occurring before midnight in other localities (Table 12). Flight dispersal is regarded as limited, generally under 1 km (0.4-2.4 km) [399].

### *Anopheles *(*Cellia*) *farauti *Laveran species complex (Farauti Complex)

The Farauti Complex is comprised of eight cryptic (isomorphic) species and shows the widest distribution and greatest genetic divergence among members in the Punctulatus Group [393,418]. The complex extends from the Maluku island group (Moluccas) in Indonesia to the western Pacific (Vanuatu) in the east. *Anopheles farauti s.s*., *An. hinesorum *Schmidt (formerly *An. farauti *No. 2) and *An. farauti *No. 4 are the only members that are considered to be important malaria vectors [391,392]. A newly identified taxon, tentatively named *An. farauti *No. 8 [419] has also been incriminated as a vector of malaria in PNG, whereas *An. farauti *No. 6 appears, based on circumstantial evidence, to be a major vector in the highland river valleys and intramontane plains of New Guinea up to 2000 m or higher [31,392]. *Anopheles farauti s.s*. has by far the widest distribution of any member in the Punctulatus Group. This species is predominately found within 1 km of coastal areas and is replaced by other members in the complex further inland [31]. Salinity tolerance appears to be a major factor in species distribution within the complex. For example, *An. hinesorum *has far less tolerance for salinity than *An. farauti *and appears restricted to inland fresh-water locations [398,409,420]. However, the degree of tolerance may be variable within a species range [397,398] (Table 5).

In general, more data are available on *An. farauti s.l*. compared to the other major malaria vectors of the Punctulatus Group yet still relatively little is known definitively about the ecology and behaviour of most of the species in the Farauti Complex. Unidentified members of the complex have been found in a wide range of aquatic habitats and adults appear to exhibit a preference for certain hosts in different regions. The time of peak biting activity also varies by locality. The heterogeneous behavioural patterns and wide range of aquatic habitats are proving to be attributable to specific differences between the individual species of the complex [391,399,409].

*Anopheles irenicus *Schmidt (formerly *An. farauti *7) lives in sympatry with *An. farauti s.s*. and appears to be restricted to the Solomon Islands [409]. Experimentally, this species shares the high salt-tolerance capabilities of *An. farauti s.s*. [397]; however, it is only found in fresh-water habitats. This species is not anthropophilic and therefore not considered to have an important role in malaria transmission, whereas *An. farauti s.s*., which readily bites humans, is an important vector in the Solomon Islands [371,388,389] and the more northerly islands such as Buka and Bougainville, PNG [390].

Larvae of *An. farauti s.l*. are commonly found in natural, rain-fed temporary pools to larger semi-permanent to permanent bodies of ground water, usually with some varying degree of floating or emergent vegetation (e.g. *Ipomoea aquatica*). They also sometimes occur in artificial containers, drums, coconut shells, canoes and other unusual habitats (Tables 9, 11). Collectively, the complex shows an ability to use a great variety of aquatic habitats. Habitat selection is also dependent on availability and influenced by dry and wet season precipitation patterns. Sites can be shaded or sunlit but usually consist of open areas away from heavy shade (canopy) (Table 5). The complex, being large and ecologically diverse, can utilise tidal and coastal brackish zones and a variety of inland fresh-water sites. *Anopheles farauti s.s*. can occupy brackish water pools high in organic debris and subject to tidal fluctuations in areas where vast stretches of mangroves occupy the coastline. Natural sites ranging from swamps (non-peat), dead river arms (oxbows) and open river flats to artificial sites including fish ponds and large ditches, burrow pits, pig wallows, garden pools and pools created along stream and river margins (Tables 7, 9). *Anopheles farauti s.s*. is not uncommonly found with *An. punctulatus *even though it generally prefers more undistributed ecology.

Feeding of *An. farauti s.l*. is mainly nocturnal and continues throughout the evening, however, daytime biting can occur. Although females will feed on birds and mammals, and when near human habitation, will often feed on domestic dogs, pigs and cattle, they have a higher proclivity for biting humans in most areas (Table 11). Host preference also appears to depend on the availability of host types. Adult females will readily attack humans inside and outside houses. Indoor resting behaviour before and after feeding occurs but habits are varied and daytime indoor resting has been observed. Individuals that feed inside houses may rest indoors for a period of time but the majority will exit before dawn to rest outdoors (Table 11). Daytime resting sites include cool, damp and darkened places near ground level.

Feeding frequency and peak activity is variable by locality and influenced by prevailing conditions and season, with some areas recording fairly uniform biting throughout the night. Other locations have seen peaks around or after midnight with other sites showing the majority of biting occurring before midnight [399,416,417]. Early evening peaks have also been recorded in some localities (Table 11). Flight dispersal is regarded as limited, with most adults remaining close to their larval sites, generally under 1 km [399,421].

### *Anopheles *(*Cellia*) *koliensis *Owen

*Anopheles koliensis *is an important vector of human malaria throughout its distribution. This species has been found naturally infected with *W. bancrofti *in PNG and the Solomon Islands. It is still regarded as a single species; however, it may be a complex of two or more cryptic species based on the recent discovery of three independently evolving rDNA genotypes that also apparently differ in biting behaviour [391]. Outside of New Guinea island, its distribution and occurrence becomes more limited and patchy [371], with some areas entirely devoid of the species despite the presence of apparently acceptable environmental conditions [390,411].

The larval habitats of *An. koliensis *appear to be intermediate between those of *An. farauti s.l*. and *An. punctulatus *[417,422]. They typically prefer more permanent collections of fresh water, such as irrigation ditches and ponds containing floating and emergent vegetation, temporary pools in open grassland and along the margins of jungle, mostly exposed to sunlight (Tables 5, 7, 9). They sometimes occur in temporary pools also preferred by *An. punctulatus*. Other sites include still pools in *Sago *swamps and shallow-water fish ponds, often in association with *An. farauti s.l*. *Anopheles koliensis *larvae are rarely, if ever, found in artificial containers and never in brackish water. Larval habitats are often in close association with human habitation.

The biting habits of *An. koliensis *have been observed on New Guinea island [377,384,391,402,412,423,424] and the Solomon Islands [425]. Females are generally strongly anthropophilic but they will also feed on animals (birds, dogs and pigs). This species readily bites outdoors and will freely enter houses to feed but does not rest indoors for long periods of time either before or after feeding [422]. Females have been found resting inside dwellings throughout the evening and during the day, but this is rare [399]. Similar to *An. punctulatus*, biting occurs throughout the night both indoors and outdoors with the greatest activity often occurring later in the evening between midnight and dawn. Feeding frequency and peak activity are variable by locality and season; in some areas biting peaks occur before midnight and in others the majority of biting occurs during the early hours of the morning [422] (Table 11).

### *Anopheles *(*Cellia*) *stephensi *Liston

*Anopheles stephensi *is an unplaced member of the Neocellia Series. It occupies a geographical range in southern Asia that extends across the Indian subcontinent with a westward extension through Iran and Iraq into the Middle East and Arabian Peninsula and eastward in Bangladesh, southern China, Myanmar and Thailand [426-428]. This species was first incriminated as a vector of malaria in Mumbai in 1911, Gujarat and Madras in 1938, Ahmedabad in 1943 and Broach in 1967 [429,430]. *Anopheles stephensi *has been recognised as an important vector of malaria in urban areas bordering the Persian Gulf, including western and northwestern India [430,431]. It includes three egg phenotypes, *mysorensis *Sweet & Rao, typical and intermediate, based on egg dimensions and the numbers of ridges on the egg float [432]. The type form is an efficient vector of urban malaria whereas the *mysorensis *form is restricted to rural areas and has a poor vectorial capacity due to its highly zoophilic behaviour [433] (Table 12). Subbarao [434] indicated that the *mysorensis *form is considered an important vector in Iran. Sporozoite rates from southern Iran have been reported to range from 0.5 to 47% [427,435-437]. The intermediate form is typically found in rural villages and peri-urban areas, but very little is known about its vector status.

Larvae of *An. stephensi *breed in various artificial containers in homes and collections of water associated with construction sites and other industrial locations. In rural areas, *An. stephensi *larvae utilise fresh-water pools, stream margins and stream beds, catch basins, seepage canals, wells and domestic water-storage containers [428] (Table 10). Larvae have also been found in domestic wells, overhead water tanks, room coolers, cisterns and roof gutters in the city of Delhi [438,439], but greater numbers of larvae are typically found outdoors compared with indoors [440]. Larvae of the *mysorensis *form appear to exclusively inhabit stone pots and earthenware containers [441].

*Anopheles stephensi *is generally considered to be an endophilic and endophagic species even though it will bite outdoors during the warmer summer months due to greater outdoor activity of humans and domestic animals [442,443]. This species rests primarily in temporary or poorly constructed human and animal shelters rather than brick structures [444]. Outdoor blood-feeding activity varies seasonally, with females feeding later in the night during the summer months compared to the winter months [445]. However, indoor biting frequencies of *An. stephensi *appear to show no marked seasonal variation during different months of the year [430] (Table 12). In rural areas of Gujarat, *An. stephensi *is associated with canal-irrigated, non-irrigated and riverine villages all year round, but generally in low densities. In urban areas, *An. stephensi *is found throughout the year, but is most abundant in the summer months (between June and August) which coincides with the peak period of malaria transmission.

Blood-meal analyses of *An. stephensi *females collected in urban areas indicated an increased tendency to feed on humans rather than cattle [446] and other indications of variable anthropophily have been observed, depending on the availability of alternative hosts [430]. For example, in Delhi the anthropological index (AI) of *An. stephensi *varies from 0.45% (near the riverine zone) to 1.40% (non-riverine zone). In Kheda (city), the AI was found to be 1.03%. However, higher AI values of 8.6% and 4.9% were recorded in the cities of Ahmedabad and Surat, respectively [430]. In addition, sporozoite rates of females in the south of Iran reportedly range between 0.5 and 47% [427,435-437,447].

### *Anopheles *(*Cellia*) *subpictus *Grassi species complex (Subpictus Complex)

The Subpictus Complex belongs to the Pyretophorus Series [6]. *Anopheles subpictus *was traditionally considered to be comprised of three subspecies, *An. s. subpictus*, *An. s. malayensis *Hacker and *An. s. indefinitus *(Ludlow), until Reid [448] concluded that the distinct morphological characteristics of their larvae indicated that *An. subpictus *was actually two separate species, *An. subpictus *and *An. indefinitus *(of which *An. s. malayensis *is a synonym), with partially overlapping distributions. *Anopheles subpictus *larvae were considered to mostly be found in brackish water habitats, with *An. indefinitus *to be primarily a fresh-water species. Reid [448], based on previous reports of distinguishing features of egg morphology, also suggested that *An. subpictus *may be a complex consisting of two or more sibling species. *Anopheles subpictus *and *An. indefinitus *were classified, along with *An. vagus *Dönitz, as being within a 'Subpictus Group' by Rattanarithikul *et al*. [29], but this grouping has not been universally recognised [6].

After the separation of the confounding fresh-water *An. indefinitus *from *An. subpictus*, further investigation identified a fresh-water type and subsequent morphological and chromosomal examinations of specimens collected from inland and coastal localities in India confirmed the existence of two sibling species [449,450], provisionally designated as species A and B. Continued investigation has led to the detection of two additional species, thus, the Subpictus Complex is currently considered to include four sibling species, designated species A, B, C and D [451]. Species B is the only species restricted to coastal brackish-water habitats [451-453], with species A, C, and D generally found in fresh-water sites including riverine pools and rice fields [451]. Abhayawardan *et al*. [452] reported the presence of species A in brackish-water coastal habitats, showing some level of salt tolerance, but densities of species A at these sites increased only after rain diluted the percentage of salinity. Indeed, Suguna *et al*. [451] reported the presence of all four species in waters with salinity ranging between 0.56 and 5.36% but it appears that only species B is found in great numbers under such conditions, or is able to tolerate the higher levels of salt content [453]. Recent investigations have, however, added doubt to some of these classifications, particularly where they are based solely on morphological characteristics. Surendran *et al*. [454] analysed rDNA from larval and adult specimens morphologically classified as species B, collected mainly from the Eastern Province of Sri Lanka. They demonstrated that the majority of these specimens were actually members of the Sundaicus Complex, another group of sibling species that are able to utilise both salt- and fresh-water larval habitats (see below). A smaller number of those specimens initially characterised as species B did belong to the Subpictus Complex but were genetically related to species A, C or D.

The Subpictus Complex has a wide distribution, ranging from northeastern Pakistan, across India, Sri Lanka, Bangladesh, Myanmar, Thailand and along coastal regions of southern Cambodia, Vietnam and coastal areas of Malaysia, Indonesia, Timor-Leste, Papua New Guinea and extending as far east as the Solomon Islands [67,154,174,181,329,345,451,452,455-473]. Species identification has not been widely reported and therefore informally named members of the complex are only known from limited areas of India, Sri Lanka, the Philippines and Thailand [451,452,471,474,475].

Larvae of the Subpictus Complex are found in both clear and turbid waters but have been reported from highly polluted habitats including sites contaminated with organic waste such as waste stabilisation ponds [476], street pools and drains [477]. Habitats may be exposed and sunlit [262,472] and larvae are frequently associated with floating algae or other vegetation [164,262,452,476,478] (Table 6). Natural larval habitats for members of the complex include lagoons, shallow ponds, marshes, slow-flowing rivers, natural pools and margins of small streams [12,158,190,191,262,467,470,472,479,480] (Tables 8, 10), but the species are also highly associated with rice fields [12,13,154,158,163,186,190,191,479-483] and irrigation schemes [13,190,479] (Tables 8, 10), specifically in the earlier stages of rice cultivation [13,163,481,482]. Larvae have also been collected from small, artificial containers, including intra-domestic earthen pots, tanks and barrels [16,479] (Table 10).

Members of the Subpictus Complex are generally zoophilic (Table 12), however species B will readily bite humans. Abhayawardana *et al*. [452] reported a high human biting rate, but this conclusion was based on only a single night of human-landing catches. Blood-meal analyses from resting collections have revealed a preference for bovine blood. For example, blood analysed from females collected from inland locations in Sri Lanka (considered to represent species A) revealed that 87.2% contained bovine blood [452]. Other studies conducted in Sri Lanka, West Bengal and Orissa, also reported high percentages of females to have fed on bovine hosts with few, if any, having fed on humans [165,458,484]. Landing collections also indicate zoophily, for example a study conducted during the implementation of the Mahaweli Development Project in eastern Sri Lanka found 37.4% of *An. subpictus *females collected were attracted to cattle compared to only 0.1% attracted to humans [10]. A similar result was reported from collections made in northwestern coastal Malaysia, with 166 females collected in cow-baited traps compared to none in human-baited nets, and only 14 captured in human-landing collections [485].

Where human-landing catches have been conducted, no clear preference for either indoor or outdoor biting has been reported [452,483] (Table 13). However, Kawada *et al*. [486], in a study conducted on Lombok Island, Indonesia, reported *An. subpictus *as one of the dominant anophelines collected outdoors. Indeed, Dash *et al*. [165] described *An. subpictus *as a zoophilic species that feeds outside and then enters houses to rest. Amerasinghe *et al*. [484] stated that *An subpictus *is the most abundant endophilic anopheline in Sri Lanka accounting for >90% of specimens collected resting indoors. The majority of studies summarised (Table 12) indicate an endophilic resting habit [157,483,487-490], with only one study conducted in Jaffna District, Sri Lanka, suggesting a higher level of exophilic behaviour [474]. However, this conclusion was based on the assumption that the collections from cattle-baited huts can be interpreted as indicative of indoor resting behaviour whereas those from cattle-baited nets indicated outdoor resting. Authors reported species B, C and D being collected in higher numbers by cattle-baited net traps.

Two recent reviews have focussed on the Subpictus Complex and the capacity of its members as vectors, but the role in malaria transmission played by each species is still not clear [26,491]. *Anopheles subpictus s.l*. is confirmed as a malaria vector in Malaysia and Indonesia [26,491] and has been reported naturally infected with malaria parasites and *W. bancrofti *in parts of eastern Indonesia (Flores, Timor and nearby islands) [194,279,492,493]. However the true identity of what has been called *An. subpictus *in Timor is questionable and may turn out to be another species [92]. Species B is frequently reported as a vector in coastal areas of southeastern India based on the work of Panicker *et al*. [494]. There is also evidence of sporozoite-positive members of the complex identified from inland areas of India and Sri Lanka [10,157,484,495], yet with limited specific information and some doubt as to the classification of species B [454], further work is needed to confirm the vectorial capacity and distribution of each species across the wide geographical range of the complex [491].

### *Anopheles *(*Cellia*) *sundaicus *(Rodenwaldt) species complex (Sundaicus Complex)

The Sundaicus Complex belongs to the Pyretophorus Series [6]. Members of the complex are predominately coastal vectors as their immature stages develop primarily in habitats containing levels of salinity ranging from low, brackish to sea water concentrations. Populations have also been recorded further inland in association with fresh water, particularly in northeastern India, Car Nicobar Island, peninsular Malaysia, Malaysian Borneo (Miri, Sarawak), northern Sumatra and Java, Indonesia [80,496-499] (Table 6). This ecological difference and behavioural heterogeneity led Reid [500] to suspect that *An. sundaicus *was a species complex. This was confirmed by Sukowati *et al*. [499,501] who provided cytogenetic and allozyme evidence for the presence of three species (informally designated species A, B and C) in Sumatra, Java and Thailand. Based on molecular approaches, *An*. *sundaicus *is currently regarded a complex of at least four species that do not exhibit ecological differences such as fresh-water/brackish-water preference [502,503]. *Anopheles sundaicus s.s*. based on a neotype from the Lundu District of Sarawak, Malaysia is distributed along the coast of Borneo [504]; *An. epiroticus *Linton & Harbach (formerly *An. sundaicus *species A) occurs most often along the mainland coastal areas from eastern India to Thailand, southern Vietnam and peninsular Malaysia; *An. sundaicus *species D appears to be restricted to the Nicobar and Andaman Islands of India [502,505]; and *An. sundaicus *species E is found in Sumatra and Java, Indonesia [503]. These four species are mainly allopatric but this does not preclude sympatry in some areas (e.g. *An. epiroticus *and *An. sundaicus *species E may coexist in northern Sumatra). A molecular assay developed to identify the species of the complex will help investigate the potential sympatry of the species [506]. The distribution of these species, especially *An. epiroticus*, often occurs in distinct foci along the coast of Thailand and Cambodia.

The immature stages generally require sunlit habitats containing pooled stagnant water, algae and non-invasive vegetation (Table 6). Filamentous floating algae and aquatic plants are crucial for the development of the larvae as they provide food (micro-algae and bacteria) and protection against predators. Particularly favourable habitats include ponds, swamps, lagoons, open mangrove, rock pools and coastal shrimp or fish ponds (active or abandoned/poorly maintained impoundments such as in Indonesia), as well as irrigated inland sea-water canals [25,496,507-510]. The close association of *An. epiroticus *with aquaculture (shrimp and fish farms) in southern Vietnam [510,511] requires special attention as this economic activity is increasing throughout Southeast Asia. With an increase in vector density, the risk for malaria epidemics, as previously recorded in Indonesia [512], is of constant concern (Table 8).

Females are mainly anthropophilic and exhibit both endophagic and exophagic feeding habits. Peak biting activity typically occurs between 20:00 h and 03:00 h depending on locality. Blood-engorged females can be found resting inside or outside houses (Table 12). Varying degrees of indoor and outdoor resting occurs and some members of the complex have been reported to be predominantly endophilic during the gonotrophic cycle.

Species of the complex are considered as either major or secondary malaria vectors depending on location [7]. They are regarded as the main vectors of malaria along the coastal areas of India, southern Vietnam and much of Indonesia [170,199,510,513,514] where they transmit both *P*. *falciparum *and *P. vivax*, and are responsible for local outbreaks [498,514-516]. However, their current role in malaria transmission along coastal areas of Thailand, Cambodia, Malaysia and Nicobar Island remains questionable [507,517,518], as well as the more recent role of *An. epiroticus *in the Mekong Delta (southern Vietnam) where it was found with a null sporozoite rate in Bac Lieu Province despite very high biting densities (12.78 bites per hour) [519]. The ecological and behavioural plasticity of species of the Sundaicus Complex poses difficulties for developing efficient and appropriate vector control strategies [25].

## Discussion

The predictive maps presented here have been created using the most up-to-date information available, including the EO species range maps, examined and updated by the TAG, many of whom have very specific and in-depth knowledge of the 19 DVS of this region. The occurrence data are maintained in what we believe to be the most comprehensive database of global DVS occurrence currently available. The climatic and environmental variables, all from open access sources, also represent what we consider to be the best data available. However, despite these efforts, the maps can still not be considered as a true and precise representation of the ranges of each of the species and species complexes.

Any species mapping process will always be limited by the available data, in terms of its quantity, quality and distribution. The methodology applied in sampling mosquitoes in any given location can have an inherent effect on the abundance, but also in some cases, on the species collected. Moreover, a great deal of work on malaria vectors will, understandably, be conducted in areas where malaria is being transmitted to humans. Thus a great deal of sampling will occur in locations near human habitations or activity, and therefore will be a spatially biased sample. Nonetheless, maps that indicate a species distribution, accepting a human 'co-variable', are clearly of use, particularly where there is a need to focus limited resources on vector control efforts. However, by applying pseudo-presences taken from within the EO range of each species or species complex, some of this bias may be removed, and a better distribution of the full range of the taxon produced. As far as we are aware, there are no other DVS maps currently available that have incorporated EO ranges within the model, and thus while the maps produced will not be the true representation of each DVS distribution, they may be the best and most accurate currently available.

### Bionomics

The bionomics summaries presented are the culmination of a joint effort by leading *Anopheles *experts. The need for continued research into the behaviour and ecology, combined with confirmed identification of the evolving and emerging sibling species and the complex status of many of the DVS in the Asian-Pacific region, is highlighted repeatedly. Simple, universal species-specific statements regarding the biology of these vectors are nearly impossible due to the locational diversity in behaviour and sympatric distributions of sibling species that contributes to a level of complexity not seen amongst the DVS of other regions. Here we have indicated the behavioural plasticity and locational variation in species behaviour where possible, and also where known and suspected species complexes exist. However, until the taxonomic situation is resolved, the behaviour of many of these DVS will remain unclear.

## Conclusions

This is the third in a series of three articles presenting the global distribution maps of 41 of the most important malaria vectors currently known [5,43,79]. In each case, the maps are presented with the caveat that they represent only the beginning of a process to establish the distribution of these DVS, and that each will be greatly improved as more data become available. Moreover, the corresponding bionomics summaries will also evolve as more information and a clarification of the taxonomy of many of these species are reported. These three articles have been produced in collaboration with a number of *Anopheles *experts, willing to share both their time and their data to ensure the best information is presented. We have been continually surprised by the generosity of the vector research community in providing data and assistance, and in this spirit, and according to the open access principles of the MAP, all our data will be made available to the research community. In return, we hope to continue improving and adapting our maps and to cultivate new collaborations to ensure we can maintain a database of the most comprehensive DVS occurrence and bionomics available.

## List of abbreviations

DVS: Dominant Vector Species; EO: Expert Opinion; BRT: Boosted Regression Tree; PAR: Population at Risk; IRS: Indoor Residual Spraying; ITNs: Insecticide Treated Bednets; TAG: Technical Advisory Group; AUC: Area Under the operating characteristic Curve; DEM: Digital Elevation Model; LST: Land Surface Temperature; MIR: Middle Infra-red Radiation; NDVI: Normalized Difference Vegetation Index; AVHRR: Advanced Very High Resolution Radiometer; PNG: Papua New Guinea; ELISA: Enzyme-Linked Immunosorbent Assay; PCR: polymerase chain reaction; PCR-RFLP: PCR-restriction-fragment length polymorphism; AI: Anthropological Index; MAP: Malaria Atlas Project.

## Competing interests

The authors declare that they have no competing interests.

## Authors' contributions

SIH conceived the study and managed its design and implementation. MES wrote the first draft of the manuscript and assembled the occurrence data with assistance from CWK and IRFE, CWK also digitised and edited all the expert opinion maps. WHT designed and maintained the databases and implemented the map figures. APP implemented the BRT scripts for predictive mapping. PWG processed the environmental and climatic data grids. Experiments were devised by SIH and MES and implemented by MES. MJB, SM, REH and TC advised on bionomics and nomenclature issues, and provided additional comments and input to the manuscript. All authors participated in the interpretation of results and in the writing and editing of the manuscript. All authors read and approved the final manuscript.

## Supplementary Material

Additional file 1 Shapefiles of the expert opinion distribution maps for the 19 DVS of the Asian-Pacific region. Click here for file

Additional file 2 Summary tables showing evaluation statistics for all mapping trials and final Boosted Regression Tree environmental and climatic variable selections for the final, optimal predictive maps. Click here for file

Additional file 3 Predictive species distribution maps for the 19 DVS of the Asian-Pacific region. Click here for file
