# Supplementary material for: The dominant Anopheles vectors of human malaria in the Asia-Pacific region: occurrence data, distribution maps and bionomic précis
Source: Parasit Vectors. 2011 May 25;4:89. doi: 10.1186/1756-3305-4-89 (PMC3127851; doi:10.1186/1756-3305-4-89)
Supplement: Additional file 2 — Summary tables showing evaluation statistics for all mapping trials and final Boosted Regression Tree environmental and climatic variable selections for the final, optimal predictive maps. [file 1756-3305-4-89-S2.DOC]

**Additional file 2:** Summary tables showing evaluation statistics for all mapping trials and final BRT environmental and climatic variable selection for final, optimal predictive maps.

**Table 2.1**. Environmental and climatic variables grids available to the BRT species mapping listing the abbreviations used in the mapping figures.

| **File name** | **Abbreviation** | **Description** |
| --- | --- | --- |
| Wd0103a0 | MIR (mean) | Middle Infrared (MIR) - mean |
| wd0103a1 | MIR (A1) | Middle Infrared (MIR) - amplitude of the annual cycle |
| wd0103a2 | MIR (A2) | Middle Infrared (MIR) - amplitude of the bi-annual cycle |
| wd0103p1 | MIR (P1) | Middle Infrared (MIR) - phase of the annual cycle |
| wd0103p2 | MIR (P2) | Middle Infrared (MIR) - phase of the bi-annual cycle |
| wd0103mn | MIR (min) | Middle Infrared (MIR) - minimum |
| wd0103mx | MIR (max) | Middle Infrared (MIR) - maximum |
| wd0107a0 | LST (mean) | Land Surface Temperature (LST) - mean |
| wd0107a1 | LST (A1) | Land Surface Temperature (LST) - amplitude of the annual cycle |
| wd0107a2 | LST (A2) | Land Surface Temperature (LST) - amplitude of the bi-annual cycle |
| wd0107p1 | LST (P1) | Land Surface Temperature (LST) - phase of the annual cycle |
| wd0107p2 | LST (P2) | Land Surface Temperature (LST) - phase of the bi-annual cycle |
| wd0107mn | LST (min) | Land Surface Temperature (LST) - minimum |
| wd0107mx | LST (max) | Land Surface Temperature (LST) - maximum |
| wd0114a0 | NDVI (mean) | Normalized Difference Vegetation Index - mean |
| wd0114a1 | NDVI (A1) | Normalized Difference Vegetation Index - amplitude of the annual cycle |
| wd0114a2 | NDVI (A2) | Normalized Difference Vegetation Index - amplitude of the bi-annual cycle |
| wd0114p1 | NDVI (P1) | Normalized Difference Vegetation Index - phase of the annual cycle |
| wd0114p2 | NDVI (P2) | Normalized Difference Vegetation Index - phase of the bi-annual cycle |
| wd0114mn | NDVI (min) | Normalized Difference Vegetation Index - minimum |
| wd0114mx | NDVI (max) | Normalized Difference Vegetation Index - maximum |

**Table 2.1 (cont.)**. Environmental and climatic variables grids available to the BRT species mapping listing the abbreviations used in the mapping figures.

| **File name** | **Abbreviation** | **Description** |
| --- | --- | --- |
| mod_dem | DEM | Digital Elevation Model (DEM) |
| mod_lst_a0 | LST (mean) | Land Surface Temperature (LST) - mean |
| mod_lst_a1 | LST (A1) | Land Surface Temperature (LST) - amplitude of the annual cycle |
| mod_lst_a2 | LST (A2) | Land Surface Temperature (LST) - amplitude of the bi-annual cycle |
| mod_lst_p1 | LST (P1) | Land Surface Temperature (LST) - phase of the annual cycle |
| mod_lst_p2 | LST (P2) | Land Surface Temperature (LST) - phase of the bi-annual cycle |
| mod_evi_a0 | EVI (mean) | Enhanced Vegetation Index (LST) - mean |
| mod_evi_a1 | EVI (A1) | Enhanced Vegetation Index (LST) - amplitude of the annual cycle |
| mod_evi_a2 | EVI (A2) | Enhanced Vegetation Index (LST) - amplitude of the bi-annual cycle |
| mod_evi_p1 | EVI (P1) | Enhanced Vegetation Index (LST) - phase of the annual cycle |
| mod_evi_p2 | EVI (P2) | Enhanced Vegetation Index (LST) - phase of the bi-annual cycle |
| prec57a0 | Prec (mean) | Precipitation - mean |
| prec57a1 | Prec (A1) | Precipitation - amplitude of the annual cycle |
| prec57a2 | Prec (A2) | Precipitation - amplitude of the bi-annual cycle |
| prec57mn | Prec (min) | Precipitation - minimum |
| prec57mx | Prec (max) | Precipitation - maximum |
| prec57p1 | Prec (P1) | Precipitation - phase of the annual cycle |
| prec57p2 | Prec (P2) | Precipitation - phase of the bi-annual cycle |
| globcover5k | GLOB (ch. no.) | See table 3.2 |
| gc5k_dry | GLOB (dry) | Globcover – dry land cover classes [140, 150, 200] – see table 3.2 |
| gc5k_flo | GLOB (flood) | Globcover – flooded land cover classes [160, 170, 180] – see table 3.2 |
| gc5k_frs | GLOB (forest) | Globcover – forested land cover classes [40, 50, 60, 90, 100] – see table 3.2 |

**Table 2.2.** Globcover channels (land cover classes) available to the BRT species mapping (Channels 210: water bodies; 220: Permanent snow and ice and 230: no data were not included in the modelling).

| **Channel** | **Description** |
| --- | --- |
| 11 | Post-flooding or irrigated croplands (or aquatic) |
| 14 | Rainfed croplands |
| 20 | Mosaic cropland (50-70%)/vegetation (grassland/shrubland/forest) (20-50%) |
| 30 | Mosaic vegetation (grassland/shrubland/forest) (50-70%)/cropland (20-50%) |
| 40 | Closed to open (>15%) broadleaved evergreen or semi-deciduous forest (>5m) |
| 50 | Closed (>40%) broadleaved deciduous forest (>5m) |
| 60 | Open (15-40%) broadleaved deciduous forest/woodland (>5m) |
| 70 | Closed (>40%) needleleaved evergreen forest (>5m) |
| 90 | Open (15-40%) needleleaved deciduous or evergreen forest (>5m) |
| 100 | Closed to open (>15%) mixed broadleaved and needleleaved forest (>5m) |
| 110 | Mosaic forest or shrubland (50-70%) / grassland (20-50%) |
| 120 | Mosaic grassland (50-70%) / forest or shrubland (20-50%) |
| 130 | Closed to open (>15%) (broadleaved or needleleaved, evergreen or deciduous) shrubland (<5m) |
| 140 | Closed to open (>15%) herbaceous vegetation (grassland, savannas or lichens/mosses) |
| 150 | Sparse (<15%) vegetation |
| 160 | Closed to open (>15%) broadleaved forest regularly flooded (semi-permanently or temporarily) - Fresh or brackish water |
| 170 | Closed (>40%) broadleaved forest or shrubland permanently flooded - Saline or brackish water |
| 180 | Closed to open (>15%) grassland or woody vegetation on regularly flooded or waterlogged soil - Fresh, brackish or saline water |
| 190 | Artificial surfaces and associated areas (Urban areas >50%) |
| 200 | Bare areas |

**Table 2.3.1**: Evaluation statistics and the top five environmental/climatic variables selected by the BRT for the 19 DVS in the Asia-Pacific region using a combination of data and 500 pseudo-presences generated within the EO range, but given a weight rating of half the true data (‘hybrid’), and 10:1 pseudo-absence:presence generated from within a 1500 km buffer area. The asterisk (*) denotes that a “species” is now recognized as a species complex.

| Species | Evaluation | | Environmental variables |
| --- | --- | --- | --- |
| *An. aconitus* (424) | Deviance: | 0.206 | | 1 | LST (P1) | | --- | --- | | 2 | Prec (A1) | | 3 | Prec (P1) | | 4 | MIR (P1) | | 5 | Prec (max) | |
|  | Correlation: | 0.858 |  |
|  | Discrimination (AUC): | 0.980 |  |
|  | Kappa: | 0.809 |  |
| *An. annularis* (496) | Deviance: | 0.182 | | 1 | LST (P1) | | --- | --- | | 2 | Prec (P1) | | 3 | Prec (max) | | 4 | Prec (A1) | | 5 | MIR (P1) | |
|  | Correlation: | 0.868 |  |
|  | Discrimination (AUC): | 0.984 |  |
|  | Kappa: | 0.822 |  |
| *An. balabacensis* (14) | Deviance: | 0.374 | | 1 | Prec (P2) | | --- | --- | | 2 | LST (mean) | | 3 | NDVI (mean) | | 4 | Prec (mean) | | 5 | Prec (P1) | |
|  | Correlation: | 0.762 |  |
|  | Discrimination (AUC): | 0.934 |  |
|  | Kappa: | 0.712 |  |
| *An. barbirostris** (872) | Deviance: | 0.156 | | 1 | Prec (max) | | --- | --- | | 2 | Prec (A1) | | 3 | LST (P1) | | 4 | Prec (min) | | 5 | Prec (P1) | |
|  | Correlation: | 0.866 |  |
|  | Discrimination (AUC): | 0.985 |  |
|  | Kappa: | 0.827 |  |
| *An. culicifacies** (550) | Deviance: | 0.186 | | 1 | NDVI (P1) | | --- | --- | | 2 | Prec (A2) | | 3 | LST (P1) | | 4 | Prec (P1) | | 5 | NDVI (min) | |
|  | Correlation: | 0.867 |  |
|  | Discrimination (AUC): | 0.977 |  |
|  | Kappa: | 0.832 |  |
| *An. dirus** (372) | Deviance: | 0.174 | | 1 | LST (P1) | | --- | --- | | 2 | Prec (A1) | | 3 | MIR (P1) | | 4 | NDVI (mean) | | 5 | LST (P2) | |
|  | Correlation: | 0.879 |  |
|  | Discrimination (AUC): | 0.986 |  |
|  | Kappa: | 0.845 |  |
| *An. farauti** (1465) | Deviance: | 0.169 | | 1 | Prec (mean) | | --- | --- | | 2 | MIR (P2) | | 3 | Prec (A1) | | 4 | Prec (P2) | | 5 | Prec (max) | |
|  | Correlation: | 0.857 |  |
|  | Discrimination (AUC): | 0.983 |  |
|  | Kappa: | 0.825 |  |
| *An. flavirostris* (103) | Deviance: | 0.333 | | 1 | Prec (mean) | | --- | --- | | 2 | Prec (P2) | | 3 | GLOB (40) | | 4 | Prec (min) | | 5 | Prec (P1) | |
|  | Correlation: | 0.844 |  |
|  | Discrimination (AUC): | 0.969 |  |
|  | Kappa: | 0.796 |  |
| *An. fluviatilis** (83) | Deviance: | 0.202 | | 1 | Prec (P1) | | --- | --- | | 2 | LST (P1) | | 3 | LST (P2) | | 4 | Prec (A2) | | 5 | NDVI (P1) | |
|  | Correlation: | 0.899 |  |
|  | Discrimination (AUC): | 0.980 |  |
|  | Kappa: | 0.869 |  |
| *An. koliensis* (325) | Deviance: | 0.134 | | 1 | Prec (mean) | | --- | --- | | 2 | LST (max) | | 3 | Prec (P2) | | 4 | LST (mean) | | 5 | Prec (min) | |
|  | Correlation: | 0.922 |  |
|  | Discrimination (AUC): | 0.993 |  |
|  | Kappa: | 0.905 |  |

**Table 2.3.2**: Evaluation statistics and the top five environmental/climatic variables selected by the BRT for the 19 DVS in the Asia-Pacific region using a combination of data and 500 pseudo-presences generated within the EO range, but given a weight rating of half the true data (‘hybrid’), and 10:1 pseudo-absence:presence generated from within a 1500 km buffer area. The asterisk (*) denotes that a “species” is now recognized as a species complex.

| Species  (no. of presence data) | Evaluation | | Environmental variables |
| --- | --- | --- | --- |
| *An. lesteri* (47) | Deviance: | 0.192 | | 1 | Prec (min) | | --- | --- | | 2 | LST (min) | | 3 | LST (A1) | | 4 | LST (mean) | | 5 | LST (P2) | |
|  | Correlation: | 0.918 |  |
|  | Discrimination (AUC): | 0.990 |  |
|  | Kappa: | 0.885 |  |
| *An. leucosphyrus & An. latens* (12) | Deviance: | 0.174 | | 1 | LST (mean) | | --- | --- | | 2 | LST (max) | | 3 | LST (P2) | | 4 | Prec (max) | | 5 | Prec (min) | |
|  | Correlation: | 0.910 |  |
|  | Discrimination (AUC): | 0.979 |  |
|  | Kappa: | 0.905 |  |
| *An. maculatus* subgroup (471) | Deviance: | 0.156 | | 1 | LST (P1) | | --- | --- | | 2 | MIR (P1) | | 3 | Prec (max) | | 4 | Prec (P1) | | 5 | NDVI (mean) | |
|  | Correlation: | 0.894 |  |
|  | Discrimination (AUC): | 0.979 |  |
|  | Kappa: | 0.868 |  |
| *An. minimus** (445) | Deviance: | 0.193 | | 1 | LST (P1) | | --- | --- | | 2 | Prec (A1) | | 3 | MIR (P1) | | 4 | LST (mean) | | 5 | LST (P2) | |
|  | Correlation: | 0.856 |  |
|  | Discrimination (AUC): | 0.979 |  |
|  | Kappa: | 0.812 |  |
| *An. punctulatus** (379) | Deviance: | 0.203 | | 1 | LST (max) | | --- | --- | | 2 | NDVI (P1) | | 3 | Prec (min) | | 4 | MIR (mean) | | 5 | MIR (max) | |
|  | Correlation: | 0.867 |  |
|  | Discrimination (AUC): | 0.982 |  |
|  | Kappa: | 0.832 |  |
| *An. sinensis* (568) | Deviance: | 0.161 | | 1 | Prec (mean) | | --- | --- | | 2 | DEM | | 3 | Prec (P1) | | 4 | Prec (A1) | | 5 | LST (mean) | |
|  | Correlation: | 0.883 |  |
|  | Discrimination (AUC): | 0.981 |  |
|  | Kappa: | 0.851 |  |
| *An. stephensi* (261) | Deviance: | 0.169 | | 1 | LST (P1) | | --- | --- | | 2 | NDVI (P1) | | 3 | Prec (min) | | 4 | Prec (P1) | | 5 | Prec (P2) | |
|  | Correlation: | 0.904 |  |
|  | Discrimination (AUC): | 0.984 |  |
|  | Kappa: | 0.878 |  |
| *An. subpictus** (410) | Deviance: | 0.281 | | 1 | LST (P1) | | --- | --- | | 2 | Prec (P1) | | 3 | Prec (A2) | | 4 | NDVI (P1) | | 5 | Prec (min) | |
|  | Correlation: | 0.819 |  |
|  | Discrimination (AUC): | 0.960 |  |
|  | Kappa: | 0.780 |  |
| *An. sundaicus** (131) | Deviance: | 0.460 | | 1 | DEM | | --- | --- | | 2 | NDVI (P1) | | 3 | Prec (mean) | | 4 | Prec (max) | | 5 | LST (mean) | |
|  | Correlation: | 0.743 |  |
|  | Discrimination (AUC): | 0.935 |  |
|  | Kappa: | 0.661 |  |

**Table 2.4.1**: Evaluation metrics of mapping trials of data only maps (‘data’); expert opinion maps where 500 pseudo-presences were generated randomly within the EO range (‘EO’); and a combination of data and 500 pseudo-presences generated within the EO range, but given a weight rating of half the true data (‘hybrid’). All maps were run using a 1000 km buffer against 1000 pseudo-absences at 5 x 5 km resolution. The asterisk (*) denotes that a “species” is now recognized as a species complex.

|  |  | Metrics | | | |
| --- | --- | --- | --- | --- | --- |
| Species  (no. of presence data) | Trial | Deviance  (0-1) | Correlation  (0-1) | Discrimination (AUC)  (0-1) | Kappa (κ)  (-1 to 1) |
| *An. aconitus* (424) | **data** | 0.233 | 0.923 | 0.990 | 0.906 |
|  | **EO** | 0.403 | 0.874 | 0.974 | 0.843 |
|  | **hybrid** | 0.302 | 0.878 | 0.977 | 0.838 |
| *An. annularis* (496) | **data** | 0.229 | 0.934 | 0.987 | 0.916 |
|  | **EO** | 0.411 | 0.875 | 0.973 | 0.853 |
|  | **hybrid** | 0.298 | 0.884 | 0.978 | 0.855 |
| *An. balabacensis* (14) | **data** | 0.110 | 0.381 | 0.832 | 0.231 |
|  | **EO** | 0.566 | 0.821 | 0.952 | 0.781 |
|  | **hybrid** | 0.425 | 0.763 | 0.936 | 0.704 |
| *An. barbirostris** (872) | **data** | 0.201 | 0.948 | 0.992 | 0.939 |
|  | **EO** | 0.420 | 0.865 | 0.972 | 0.829 |
|  | **hybrid** | 0.252 | 0.899 | 0.984 | 0.862 |
| *An. culicifacies** (550) | **data** | 0.226 | 0.934 | 0.990 | 0.917 |
|  | **EO** | 0.454 | 0.858 | 0.968 | 0.824 |
|  | **hybrid** | 0.302 | 0.885 | 0.975 | 0.854 |
| *An. dirus** (372) | **data** | 0.176 | 0.935 | 0.994 | 0.918 |
|  | **EO** | 0.426 | 0.868 | 0.971 | 0.831 |
|  | **hybrid** | 0.272 | 0.889 | 0.980 | 0.859 |
| *An. farauti** (1465) | **data** | 0.266 | 0.925 | 0.982 | 0.901 |
|  | **EO** | 0.459 | 0.852 | 0.967 | 0.804 |
|  | **hybrid** | 0.303 | 0.883 | 0.974 | 0.851 |
| *An. flavirostris* (103) | **data** | 0.141 | 0.880 | 0.989 | 0.844 |
|  | **EO** | 0.547 | 0.819 | 0.954 | 0.776 |
|  | **hybrid** | 0.406 | 0.800 | 0.952 | 0.739 |
| *An. fluviatilis** (83) | **data** | 0.209 | 0.859 | 0.985 | 0.815 |
|  | **EO** | 0.264 | 0.926 | 0.989 | 0.917 |
|  | **hybrid** | 0.262 | 0.896 | 0.981 | 0.861 |
| *An. koliensis* (325) | **data** | 0.172 | 0.931 | 0.994 | 0.905 |
|  | **EO** | 0.355 | 0.894 | 0.978 | 0.867 |
|  | **hybrid** | 0.273 | 0.893 | 0.981 | 0.864 |

**Table 2.4.2**: Evaluation metrics of mapping trials of data only maps (‘data’); expert opinion maps where 500 pseudo-presences were generated randomly within the EO range (‘EO’); and a combination of data and 500 pseudo-presences generated within the EO range, but given a weight rating of half the true data (‘hybrid’). All maps were run using a 1000 km buffer against 1000 pseudo-absences at 5 x 5 km resolution. The asterisk (*) denotes that a “species” is now recognized as a species complex.

|  |  | Metrics | | | |
| --- | --- | --- | --- | --- | --- |
| Species  (no. of presence data) | Trial | Deviance  (0-1) | Correlation  (0-1) | Discrimination (AUC)  (0-1) | Kappa (κ)  (-1 to 1) |
| *An. lesteri* (47) | **data** | 0.105 | 0.840 | 0.981 | 0.834 |
|  | **EO** | 0.384 | 0.886 | 0.976 | 0.863 |
|  | **hybrid** | 0.277 | 0.865 | 0.977 | 0.819 |
| *An. leucosphyrus & An. latens* (12) | **data** | 0.055 | 0.757 | 0.902 | 0.665 |
|  | **EO** | 0.352 | 0.891 | 0.979 | 0.857 |
|  | **hybrid** | 0.270 | 0.864 | 0.976 | 0.813 |
| *An. maculatus* subgroup (471) | **data** | 0.149 | 0.959 | 0.994 | 0.950 |
|  | **EO** | 0.382 | 0.888 | 0.977 | 0.868 |
|  | **hybrid** | 0.232 | 0.902 | 0.985 | 0.878 |
| *An. minimus** (445) | **data** | 0.243 | 0.924 | 0.988 | 0.915 |
|  | **EO** | 0.488 | 0.844 | 0.963 | 0.807 |
|  | **hybrid** | 0.324 | 0.869 | 0.972 | 0.833 |
| *An. punctulatus** (379) | **data** | 0.231 | 0.918 | 0.989 | 0.899 |
|  | **EO** | 0.420 | 0.873 | 0.972 | 0.836 |
|  | **hybrid** | 0.303 | 0.880 | 0.978 | 0.847 |
| *An. sinensis* (568) | **data** | 0.168 | 0.952 | 0.994 | 0.938 |
|  | **EO** | 0.441 | 0.866 | 0.970 | 0.837 |
|  | **hybrid** | 0.253 | 0.904 | 0.981 | 0.878 |
| *An. stephensi* (261) | **data** | 0.165 | 0.936 | 0.990 | 0.920 |
|  | **EO** | 0.418 | 0.869 | 0.974 | 0.833 |
|  | **hybrid** | 0.295 | 0.878 | 0.975 | 0.850 |
| *An. subpictus** (410) | **data** | 0.402 | 0.847 | 0.970 | 0.814 |
|  | **EO** | 0.515 | 0.839 | 0.960 | 0.803 |
|  | **hybrid** | 0.432 | 0.835 | 0.957 | 0.798 |
| *An. sundaicus** (131) | **data** | 0.311 | 0.754 | 0.962 | 0.700 |
|  | **EO** | 0.654 | 0.777 | 0.928 | 0.736 |
|  | **hybrid** | 0.484 | 0.754 | 0.933 | 0.693 |

**Table 2.5.1**: Evaluation statistics for a range of buffer sizes. All maps were run using ‘hybrid’ data (a combination of data and 500 pseudo-presences generated within the EO range, but given a weight rating of half the true data) against 1000 pseudo-absences at 5 x 5 km resolution. The asterisk (*) denotes that a “species” is now recognized as a species complex.

|  |  | Metrics | | | |
| --- | --- | --- | --- | --- | --- |
| Species  (no. of presence data) | Buffer size (km) | Deviance  (0-1) | Correlation  (0-1) | Discrimination (AUC)  (0-1) | Kappa (κ)  (-1 to 1) |
| *An. aconitus* (424) | **500** | 0.299 | 0.877 | 0.976 | 0.848 |
|  | **1000** | 0.233 | 0.923 | 0.990 | 0.906 |
|  | **1500** | 0.288 | 0.888 | 0.978 | 0.850 |
| *An. annularis* (496) | **500** | 0.319 | 0.872 | 0.975 | 0.834 |
|  | **1000** | 0.229 | 0.934 | 0.987 | 0.916 |
|  | **1500** | 0.264 | 0.900 | 0.982 | 0.874 |
| *An. balabacensis* (14) | **500** | 0.465 | 0.727 | 0.917 | 0.663 |
|  | **1000** | 0.110 | 0.381 | 0.832 | 0.231 |
|  | **1500** | 0.419 | 0.768 | 0.939 | 0.696 |
| *An. barbirostris** (872) | **500** | 0.241 | 0.905 | 0.984 | 0.888 |
|  | **1000** | 0.201 | 0.948 | 0.992 | 0.939 |
|  | **1500** | 0.237 | 0.906 | 0.985 | 0.878 |
| *An. culicifacies** (550) | **500** | 0.325 | 0.868 | 0.973 | 0.829 |
|  | **1000** | 0.226 | 0.934 | 0.990 | 0.917 |
|  | **1500** | 0.238 | 0.909 | 0.986 | 0.893 |
| *An. dirus** (372) | **500** | 0.341 | 0.857 | 0.970 | 0.819 |
|  | **1000** | 0.176 | 0.935 | 0.994 | 0.918 |
|  | **1500** | 0.233 | 0.908 | 0.985 | 0.885 |
| *An. farauti** (1465) | **500** | 0.314 | 0.876 | 0.973 | 0.849 |
|  | **1000** | 0.266 | 0.925 | 0.982 | 0.901 |
|  | **1500** | 0.254 | 0.903 | 0.982 | 0.875 |
| *An. flavirostris* (103) | **500** | 0.442 | 0.779 | 0.940 | 0.707 |
|  | **1000** | 0.141 | 0.880 | 0.989 | 0.844 |
|  | **1500** | 0.333 | 0.845 | 0.968 | 0.798 |
| *An. fluviatilis** (83) | **500** | 0.269 | 0.885 | 0.976 | 0.850 |
|  | **1000** | 0.209 | 0.859 | 0.985 | 0.815 |
|  | **1500** | 0.297 | 0.881 | 0.974 | 0.856 |
| *An. koliensis* (325) | **500** | 0.343 | 0.849 | 0.970 | 0.792 |
|  | **1000** | 0.172 | 0.931 | 0.994 | 0.905 |
|  | **1500** | 0.207 | 0.927 | 0.989 | 0.909 |

**Table 2.5.2:** Evaluation statistics for a range of buffer sizes. All maps were run using ‘hybrid’ data (a combination of data and 500 pseudo-presences generated within the EO range, but given a weight rating of half the true data) against 1000 pseudo-absences at 5 x 5 km resolution. The asterisk (*) denotes that a “species” is now recognized as a species complex.

|  |  | Metrics | | | |
| --- | --- | --- | --- | --- | --- |
| Species  (no. of presence data) | Buffer size (km) | Deviance  (0-1) | Correlation  (0-1) | Discrimination (AUC)  (0-1) | Kappa (κ)  (-1 to 1) |
| *An. lesteri* (47) | **500** | 0.303 | 0.841 | 0.970 | 0.784 |
|  | **1000** | 0.277 | 0.865 | 0.977 | 0.819 |
|  | **1500** | 0.210 | 0.900 | 0.986 | 0.870 |
| *An. leucosphyrus & An. latens* (12) | **500** | 0.327 | 0.828 | 0.963 | 0.779 |
|  | **1000** | 0.055 | 0.757 | 0.902 | 0.665 |
|  | **1500** | 0.238 | 0.885 | 0.982 | 0.855 |
| *An. maculatus* subgroup (471) | **500** | 0.316 | 0.863 | 0.971 | 0.829 |
|  | **1000** | 0.149 | 0.959 | 0.994 | 0.950 |
|  | **1500** | 0.245 | 0.901 | 0.982 | 0.874 |
| *An. minimus** (445) | **500** | 0.377 | 0.847 | 0.960 | 0.821 |
|  | **1000** | 0.243 | 0.924 | 0.988 | 0.915 |
|  | **1500** | 0.296 | 0.883 | 0.978 | 0.854 |
| *An. punctulatus** (379) | **500** | 0.424 | 0.819 | 0.956 | 0.762 |
|  | **1000** | 0.231 | 0.918 | 0.989 | 0.899 |
|  | **1500** | 0.254 | 0.910 | 0.983 | 0.887 |
| *An. sinensis* (568) | **500** | 0.284 | 0.887 | 0.976 | 0.866 |
|  | **1000** | 0.168 | 0.952 | 0.994 | 0.938 |
|  | **1500** | 0.245 | 0.903 | 0.982 | 0.885 |
| *An. stephensi* (261) | **500** | 0.283 | 0.886 | 0.976 | 0.854 |
|  | **1000** | 0.165 | 0.936 | 0.990 | 0.920 |
|  | **1500** | 0.252 | 0.901 | 0.981 | 0.884 |
| *An. subpictus** (410) | **500** | 0.433 | 0.824 | 0.953 | 0.792 |
|  | **1000** | 0.402 | 0.847 | 0.970 | 0.814 |
|  | **1500** | 0.393 | 0.848 | 0.964 | 0.813 |
| *An. sundaicus** (131) | **500** | 0.607 | 0.664 | 0.893 | 0.547 |
|  | **1000** | 0.311 | 0.754 | 0.962 | 0.700 |
|  | **1500** | 0.429 | 0.787 | 0.948 | 0.704 |

**Table 2.6.1**: Evaluation statistics for a range of pseudo-absence:occurrence data ratios and constant values. All maps were run at a 5 x 5 km resolution using ‘hybrid’ data (a combination of data and 500 pseudo-presences generated within the EO range, but given a weight rating of half the true data) with the pseudo-absences taken from within a 1000km buffer. The asterisk (*) denotes that a “species” is now recognized as a species complex.

|  |  | Metrics | | | |
| --- | --- | --- | --- | --- | --- |
| Species  (no. of presence data) | Pseudo-absence:presence | Deviance  (0-1) | Correlation  (0-1) | Discrimination (AUC)  (0-1) | Kappa (κ)  (-1 to 1) |
| *An. aconitus* (424) | **1:1** | 0.271 | 0.881 | 0.977 | 0.849 |
|  | **2:1** | 0.300 | 0.882 | 0.978 | 0.845 |
|  | **5:1** | 0.253 | 0.884 | 0.980 | 0.854 |
|  | **10:1** | 0.195 | 0.867 | 0.978 | 0.825 |
|  | **1000 points** | 0.302 | 0.878 | 0.977 | 0.838 |
| *An. annularis* (496) | **1:1** | 0.265 | 0.889 | 0.980 | 0.865 |
|  | **2:1** | 0.328 | 0.874 | 0.974 | 0.851 |
|  | **5:1** | 0.251 | 0.878 | 0.979 | 0.844 |
|  | **10:1** | 0.194 | 0.861 | 0.979 | 0.821 |
|  | **1000 points** | 0.298 | 0.884 | 0.978 | 0.855 |
| *An. balabacensis* (14) | **1:1** | 0.145 | 0.495 | 0.841 | 0.127 |
|  | **2:1** | 0.277 | 0.359 | 0.788 | 0.117 |
|  | **5:1** | 0.413 | 0.549 | 0.868 | 0.424 |
|  | **10:1** | 0.430 | 0.708 | 0.924 | 0.633 |
|  | **1000 points** | 0.425 | 0.763 | 0.936 | 0.704 |
| *An. barbirostris** (872) | **1:1** | 0.271 | 0.888 | 0.980 | 0.851 |
|  | **2:1** | 0.263 | 0.894 | 0.982 | 0.862 |
|  | **5:1** | 0.214 | 0.881 | 0.983 | 0.843 |
|  | **10:1** | 0.159 | 0.863 | 0.983 | 0.814 |
|  | **1000 points** | 0.252 | 0.899 | 0.984 | 0.862 |
| *An. culicifacies** (550) | **1:1** | 0.350 | 0.850 | 0.967 | 0.826 |
|  | **2:1** | 0.283 | 0.889 | 0.979 | 0.859 |
|  | **5:1** | 0.264 | 0.868 | 0.973 | 0.830 |
|  | **10:1** | 0.197 | 0.852 | 0.974 | 0.809 |
|  | **1000 points** | 0.302 | 0.885 | 0.975 | 0.854 |

**Table 2.6.2**: Evaluation statistics for a range of pseudo-absence:occurrence data ratios and constant values. All maps were run at a 5 x 5 km resolution using ‘hybrid’ data (a combination of data and 500 pseudo-presences generated within the EO range, but given a weight rating of half the true data) with the pseudo-absences taken from within a 1000km buffer. The asterisk (*) denotes that a “species” is now recognized as a species complex.

|  |  | Metrics | | | |
| --- | --- | --- | --- | --- | --- |
| Species  (no. of presence data) | Pseudo-absence:presence | Deviance  (0-1) | Correlation  (0-1) | Discrimination (AUC)  (0-1) | Kappa (κ)  (-1 to 1) |
| *An. dirus** (372) | **1:1** | 0.281 | 0.860 | 0.976 | 0.821 |
|  | **2:1** | 0.292 | 0.878 | 0.976 | 0.842 |
|  | **5:1** | 0.257 | 0.872 | 0.980 | 0.838 |
|  | **10:1** | 0.201 | 0.859 | 0.980 | 0.807 |
|  | **1000 points** | 0.272 | 0.889 | 0.980 | 0.859 |
| *An. farauti** (1465) | **1:1** | 0.289 | 0.898 | 0.980 | 0.870 |
|  | **2:1** | 0.276 | 0.902 | 0.982 | 0.881 |
|  | **5:1** | 0.244 | 0.873 | 0.980 | 0.849 |
|  | **10:1** | 0.188 | 0.838 | 0.980 | 0.802 |
|  | **1000 points** | 0.303 | 0.883 | 0.974 | 0.851 |
| *An. flavirostris* (103) | **1:1** | 0.306 | 0.788 | 0.925 | 0.749 |
|  | **2:1** | 0.405 | 0.781 | 0.936 | 0.730 |
|  | **5:1** | 0.381 | 0.842 | 0.960 | 0.802 |
|  | **10:1** | 0.429 | 0.786 | 0.945 | 0.716 |
|  | **1000 points** | 0.406 | 0.800 | 0.952 | 0.739 |
| *An. fluviatilis** (83) | **1:1** | 0.270 | 0.836 | 0.968 | 0.792 |
|  | **2:1** | 0.339 | 0.854 | 0.966 | 0.826 |
|  | **5:1** | 0.295 | 0.887 | 0.977 | 0.854 |
|  | **10:1** | 0.201 | 0.903 | 0.980 | 0.879 |
|  | **1000 points** | 0.262 | 0.896 | 0.981 | 0.861 |
| *An. koliensis* (325) | **1:1** | 0.245 | 0.899 | 0.976 | 0.864 |
|  | **2:1** | 0.236 | 0.912 | 0.986 | 0.887 |
|  | **5:1** | 0.225 | 0.900 | 0.986 | 0.872 |
|  | **10:1** | 0.185 | 0.878 | 0.986 | 0.836 |
|  | **1000 points** | 0.273 | 0.893 | 0.981 | 0.864 |

**Table 2.6.3**: Evaluation statistics for a range of pseudo-absence:occurrence data ratios and constant values. All maps were run at a 5 x 5 km resolution using ‘hybrid’ data (a combination of data and 500 pseudo-presences generated within the EO range, but given a weight rating of half the true data) with the pseudo-absences taken from within a 1000km buffer. The asterisk (*) denotes that a “species” is now recognized as a species complex.

|  |  | Metrics | | | |
| --- | --- | --- | --- | --- | --- |
| Species  (no. of presence data) | Pseudo-absence:presence | Deviance  (0-1) | Correlation  (0-1) | Discrimination (AUC)  (0-1) | Kappa (κ)  (-1 to 1) |
| *An. lesteri* (47) | **1:1** | 0.209 | 0.743 | 0.945 | 0.648 |
|  | **2:1** | 0.223 | 0.816 | 0.968 | 0.774 |
|  | **5:1** | 0.266 | 0.867 | 0.974 | 0.830 |
|  | **10:1** | 0.265 | 0.886 | 0.980 | 0.864 |
|  | **1000 points** | 0.277 | 0.865 | 0.977 | 0.819 |
| *An. leucosphyrus & An. latens* (12) | **1:1** | 0.088 | 0.657 | 0.960 | 0.464 |
|  | **2:1** | 0.157 | 0.710 | 0.941 | 0.645 |
|  | **5:1** | 0.148 | 0.875 | 0.978 | 0.840 |
|  | **10:1** | 0.232 | 0.876 | 0.970 | 0.841 |
|  | **1000 points** | 0.270 | 0.864 | 0.976 | 0.813 |
| *An. maculatus* subgroup (471) | **1:1** | 0.293 | 0.860 | 0.973 | 0.825 |
|  | **2:1** | 0.277 | 0.884 | 0.978 | 0.850 |
|  | **5:1** | 0.257 | 0.871 | 0.976 | 0.836 |
|  | **10:1** | 0.168 | 0.884 | 0.977 | 0.854 |
|  | **1000 points** | 0.232 | 0.902 | 0.985 | 0.878 |
| *An. minimus** (445) | **1:1** | 0.322 | 0.856 | 0.970 | 0.826 |
|  | **2:1** | 0.326 | 0.870 | 0.971 | 0.833 |
|  | **5:1** | 0.267 | 0.868 | 0.975 | 0.828 |
|  | **10:1** | 0.212 | 0.840 | 0.973 | 0.786 |
|  | **1000 points** | 0.324 | 0.869 | 0.972 | 0.833 |
| *An. punctulatus** (379) | **1:1** | 0.306 | 0.867 | 0.969 | 0.830 |
|  | **2:1** | 0.302 | 0.886 | 0.977 | 0.854 |
|  | **5:1** | 0.272 | 0.878 | 0.979 | 0.853 |
|  | **10:1** | 0.230 | 0.848 | 0.975 | 0.810 |
|  | **1000 points** | 0.303 | 0.880 | 0.978 | 0.847 |

**Table 2.6.4**: Evaluation statistics for a range of pseudo-absence:occurrence data ratios and constant values. All maps were run at a 5 x 5 km resolution using ‘hybrid’ data (a combination of data and 500 pseudo-presences generated within the EO range, but given a weight rating of half the true data) with the pseudo-absences taken from within a 1000km buffer. The asterisk (*) denotes that a “species” is now recognized as a species complex.

|  |  | Metrics | | | |
| --- | --- | --- | --- | --- | --- |
| Species  (no. of presence data) | Pseudo-absence:presence | Deviance  (0-1) | Correlation  (0-1) | Discrimination (AUC)  (0-1) | Kappa (κ)  (-1 to 1) |
| *An. sinensis* (568) | **1:1** | 0.286 | 0.877 | 0.975 | 0.846 |
|  | **2:1** | 0.303 | 0.880 | 0.975 | 0.855 |
|  | **5:1** | 0.217 | 0.894 | 0.979 | 0.868 |
|  | **10:1** | 0.319 | 0.874 | 0.970 | 0.859 |
|  | **1000 points** | 0.253 | 0.904 | 0.981 | 0.878 |
| *An. stephensi* (261) | **1:1** | 0.280 | 0.855 | 0.972 | 0.815 |
|  | **2:1** | 0.252 | 0.899 | 0.984 | 0.868 |
|  | **5:1** | 0.236 | 0.896 | 0.983 | 0.861 |
|  | **10:1** | 0.205 | 0.880 | 0.979 | 0.839 |
|  | **1000 points** | 0.295 | 0.878 | 0.975 | 0.850 |
| *An. subpictus** (410) | **1:1** | 0.440 | 0.800 | 0.948 | 0.758 |
|  | **2:1** | 0.416 | 0.836 | 0.960 | 0.808 |
|  | **5:1** | 0.365 | 0.834 | 0.957 | 0.799 |
|  | **10:1** | 0.274 | 0.825 | 0.957 | 0.778 |
|  | **1000 points** | 0.432 | 0.835 | 0.957 | 0.798 |
| *An. sundaicus** (131) | **1:1** | 0.319 | 0.790 | 0.935 | 0.754 |
|  | **2:1** | 0.516 | 0.733 | 0.901 | 0.685 |
|  | **5:1** | 0.550 | 0.735 | 0.915 | 0.652 |
|  | **10:1** | 0.487 | 0.727 | 0.930 | 0.636 |
|  | **1000 points** | 0.484 | 0.754 | 0.933 | 0.693 |
