# Supplementary material for: The dominant Anopheles vectors of human malaria in the Asia-Pacific region: occurrence data, distribution maps and bionomic précis
Source: Parasit Vectors. 2011 May 25;4:89. doi: 10.1186/1756-3305-4-89 (PMC3127851; doi:10.1186/1756-3305-4-89)

Anopheles (*Cellia*) *aconitus* Dönitz, 1902

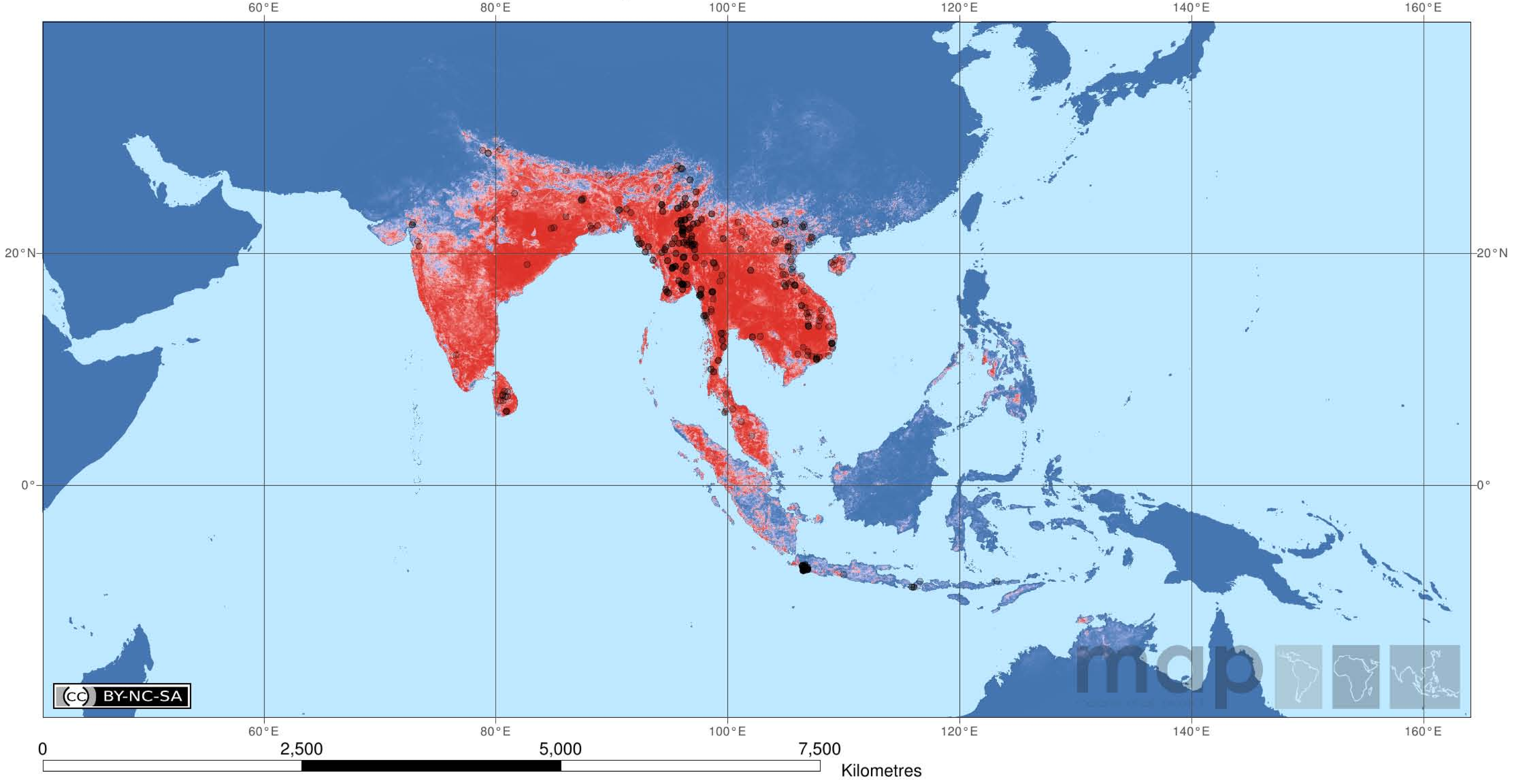

Mapping details: This map shows the predicted probability of occurrence of *An. aconitus* in Asia-Pacific. The map was created with the Boosted Regression Trees (BRT) technique using 424 occurrence points, 500 pseudo occurrence points generated from a stratified random sample within the expert opinion range (see inset), balanced by 4,240 pseudo absence points sampled within a 1,500 km buffer outside the expert opinion range. The pseudo presence data were given half the weight of observed occurrence data. Predictions are not shown beyond the 1,500 km buffer. The black dots show 424 records of occurrence for *An. aconitus* as detailed in Hay *et al.* [1].

Map statistics: Deviance=0.2059, Correlation=0.8577, Discrimination (AUC)=0.9801, Kappa=0.8085.

Environmental variables used: 1. LST (P1), 2. Prec (A1), 3. Prec (P1), 4. MIR (P1) and 5. Prec (max). Please see additional file 2 for abbreviations and definitions.

Copyright: Licensed to the Malaria Atlas Project (MAP; www.map.ox.ac.uk) under a Creative Commons Attribution 3.0 License (http://creativecommons.org/).

Citation: Sinka *et al.* (2011). The dominant *Anopheles* vectors of human malaria in the Asia-Pacific region: occurrence data, distribution maps and bionomic précis. Parasites & Vectors, 4: 89.

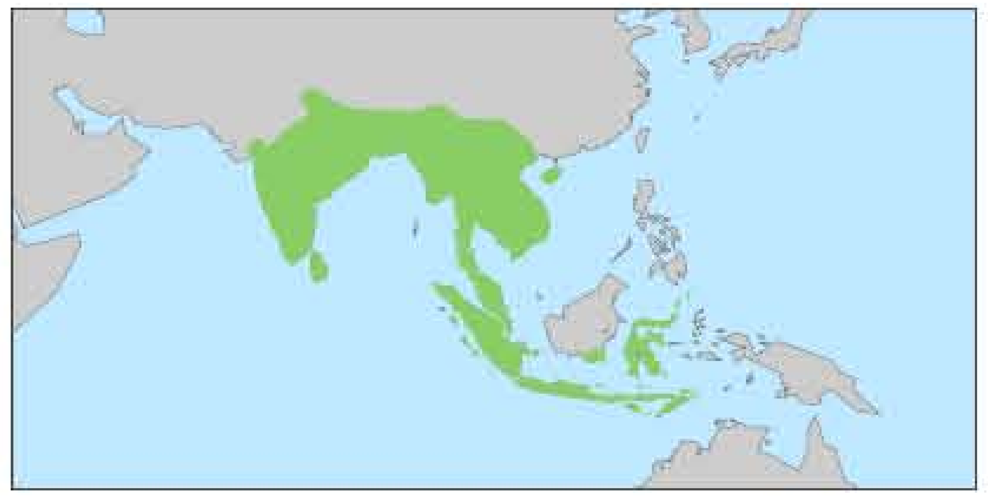

Probability

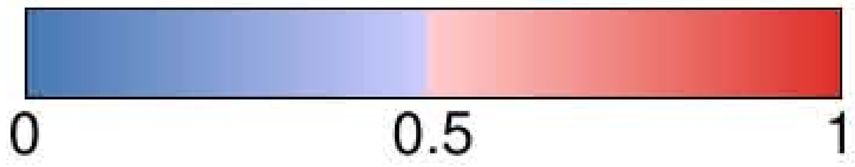

N

Anopheles (*Cellia*) *annularis* van der Wulp, 1884

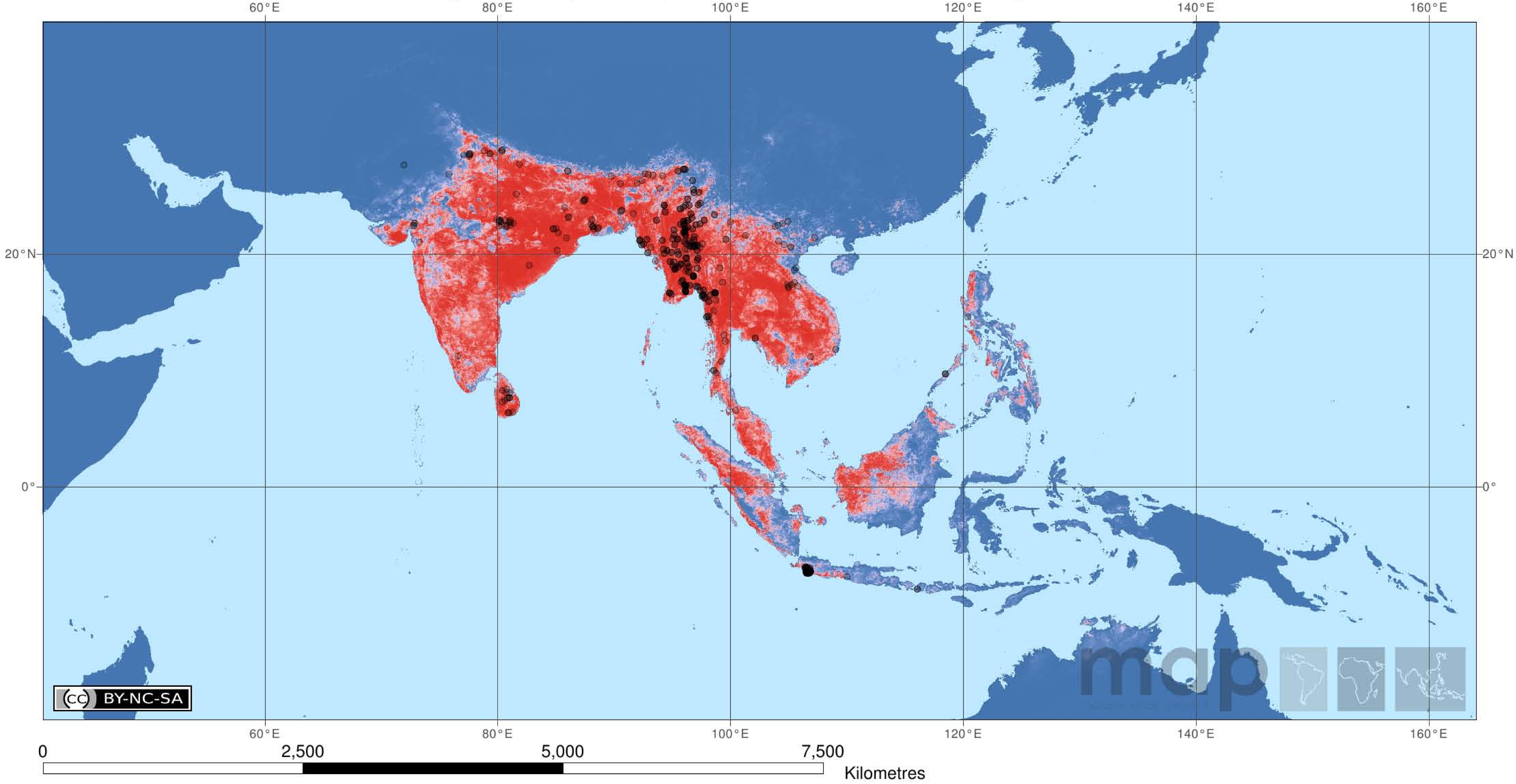

Mapping details: This map shows the predicted probability of occurrence of *An. annularis* in Asia-Pacific. The map was created with the Boosted Regression Trees (BRT) technique using 496 occurrence points, 500 pseudo occurrence points generated from a stratified random sample within the expert opinion range (see inset), balanced by 4,960 pseudo absence points sampled within a 1,500 km buffer outside the expert opinion range. The pseudo presence data were given half the weight of observed occurrence data. Predictions are not shown beyond the 1,500 km buffer. The black dots show 496 records of occurrence for *An. annularis* as detailed in Hay *et al.* [1].

Map statistics: Deviance=0.1822, Correlation=0.8676, Discrimination (AUC)=0.9843, Kappa=0.8222.

Environmental variables used: 1. LST (P1), 2. Prec (P1), 3. Prec (max), 4. Prec (A1) and 5. MIR (P1). Please see additional file 2 for abbreviations and definitions.

Copyright: Licensed to the Malaria Atlas Project (MAP; www.map.ox.ac.uk) under a Creative Commons Attribution 3.0 License (http://creativecommons.org/).

Citation: Sinka *et al.* (2011). The dominant *Anopheles* vectors of human malaria in the Asia-Pacific region: occurrence data, distribution maps and bionomic précis. Parasites & Vectors, 4: 89.

N

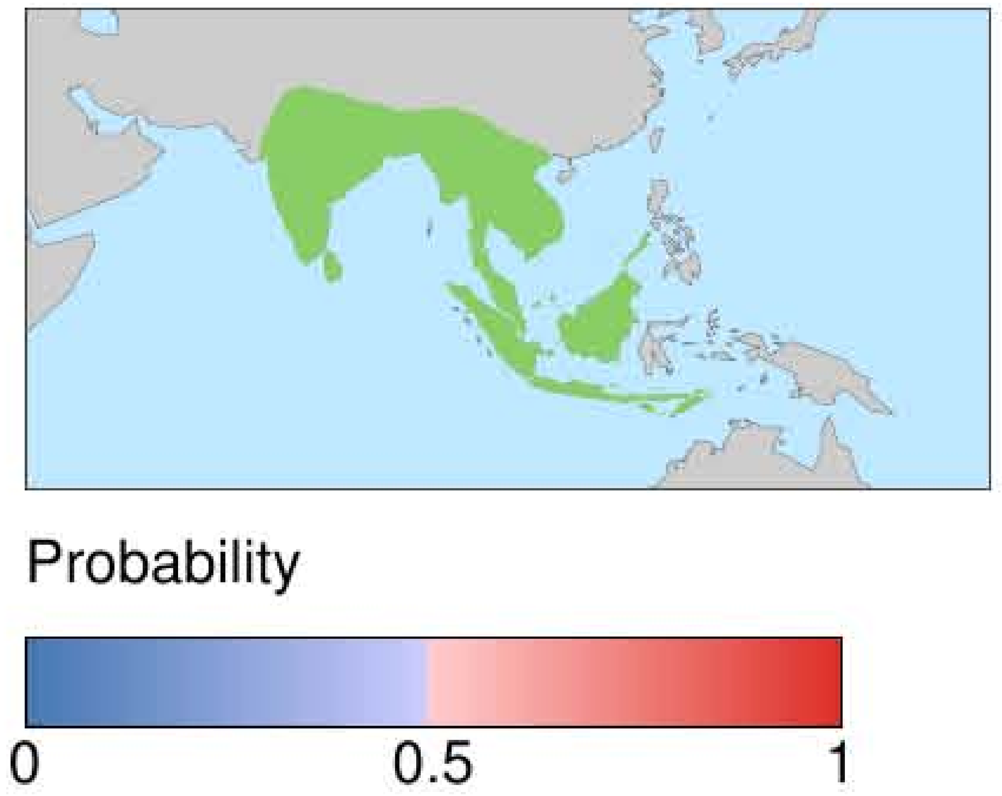

Anopheles (*Cellia*) *balabacensis* Baisas, 1936

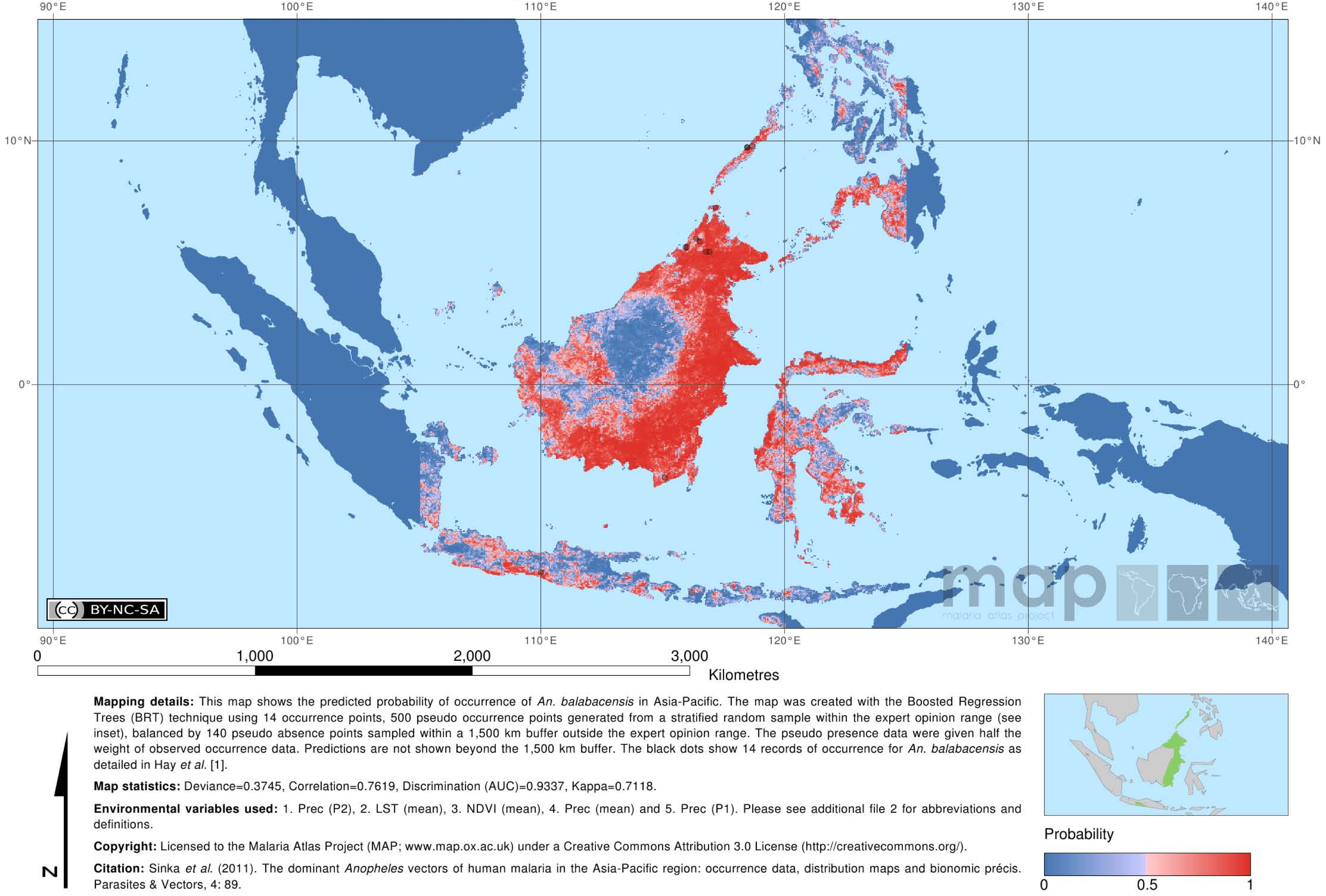

Mapping details: This map shows the predicted probability of occurrence of *An. balabacensis* in Asia-Pacific. The map was created with the Boosted Regression Trees (BRT) technique using 14 occurrence points, 500 pseudo occurrence points generated from a stratified random sample within the expert opinion range (see inset), balanced by 140 pseudo absence points sampled within a 1,500 km buffer outside the expert opinion range. The pseudo presence data were given half the weight of observed occurrence data. Predictions are not shown beyond the 1,500 km buffer. The black dots show 14 records of occurrence for *An. balabacensis* as detailed in Hay *et al.* [1].

Map statistics: Deviance=0.3745, Correlation=0.7619, Discrimination (AUC)=0.9337, Kappa=0.7118.

Environmental variables used: 1. Prec (P2), 2. LST (mean), 3. NDVI (mean), 4. Prec (mean) and 5. Prec (P1). Please see additional file 2 for abbreviations and definitions.

Copyright: Licensed to the Malaria Atlas Project (MAP; www.map.ox.ac.uk) under a Creative Commons Attribution 3.0 License (http://creativecommons.org/).

Citation: Sinka *et al.* (2011). The dominant *Anopheles* vectors of human malaria in the Asia-Pacific region: occurrence data, distribution maps and bionomic précis. Parasites & Vectors, 4: 89.

Anopheles (*Anopheles*) *barbirostris* species complex

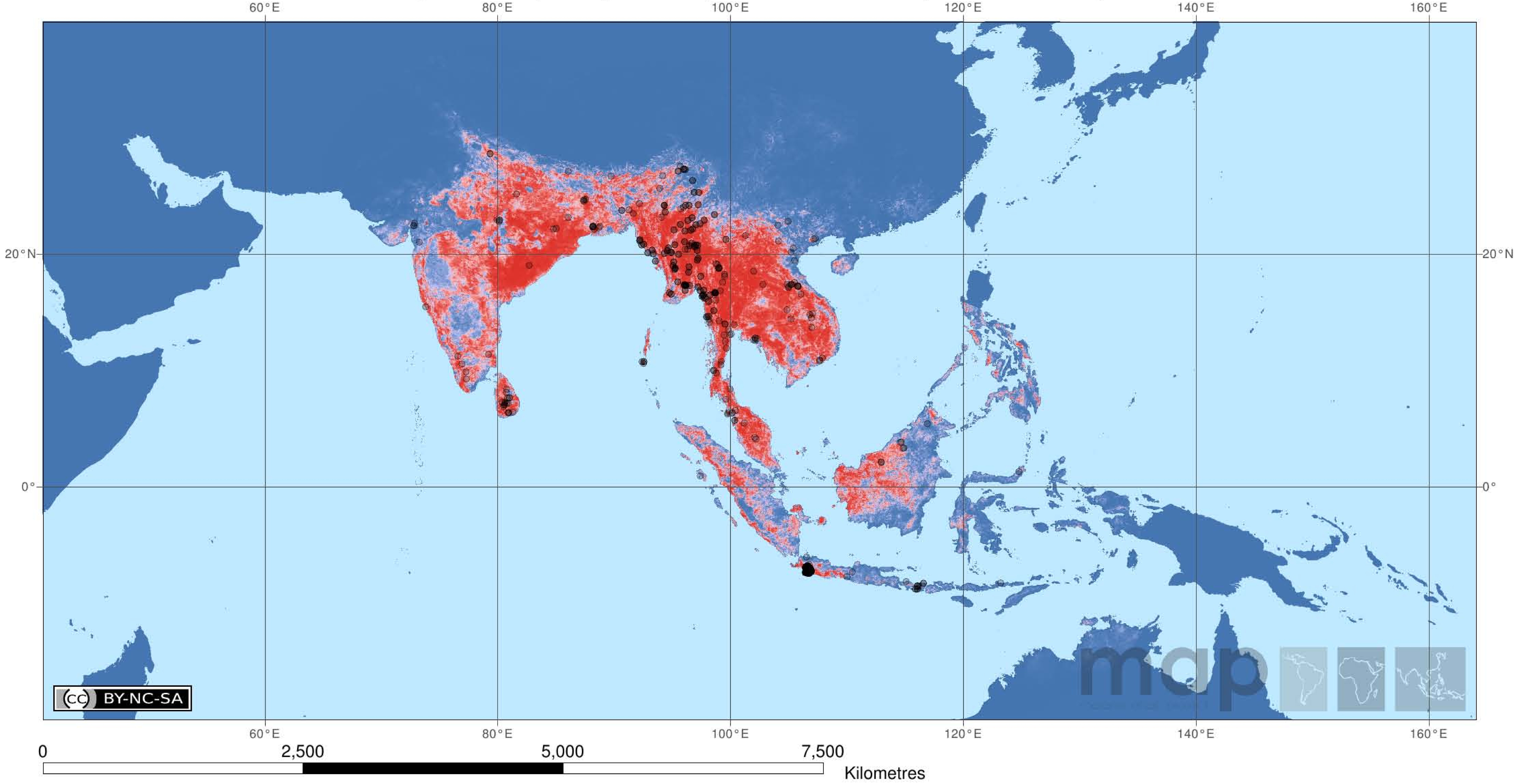

N

Mapping details: This map shows the predicted probability of occurrence of *An. barbirostris* in Asia-Pacific. The map was created with the Boosted Regression Trees (BRT) technique using 872 occurrence points, 500 pseudo occurrence points generated from a stratified random sample within the expert opinion range (see inset), balanced by 8,720 pseudo absence points sampled within a 1,500 km buffer outside the expert opinion range. The pseudo presence data were given half the weight of observed occurrence data. Predictions are not shown beyond the 1,500 km buffer. The black dots show 872 records of occurrence for *An. barbirostris* as detailed in Hay *et al.* [1].

Map statistics: Deviance=0.1562, Correlation=0.8655, Discrimination (AUC)=0.9853, Kappa=0.8267.

Environmental variables used: 1. Prec (max), 2. Prec (A1), 3. LST (P1), 4. Prec (min) and 5. Prec (P1). Please see additional file 2 for abbreviations and definitions.

Copyright: Licensed to the Malaria Atlas Project (MAP; www.map.ox.ac.uk) under a Creative Commons Attribution 3.0 License (http://creativecommons.org/).

Citation: Sinka *et al.* (2011). The dominant *Anopheles* vectors of human malaria in the Asia-Pacific region: occurrence data, distribution maps and bionomic précis. Parasites & Vectors, 4: 89.

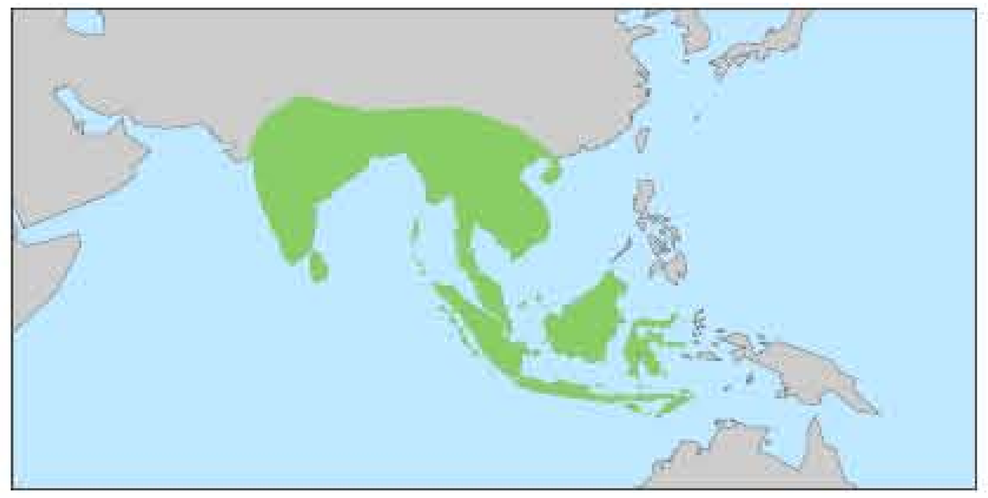

Probability

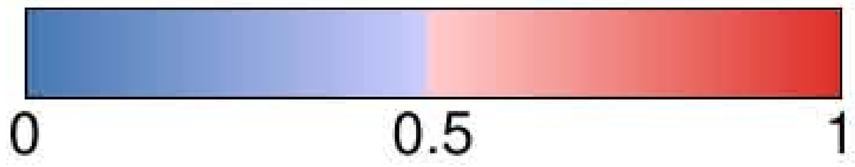

Anopheles (*Cellia*) *culicifacies* species complex

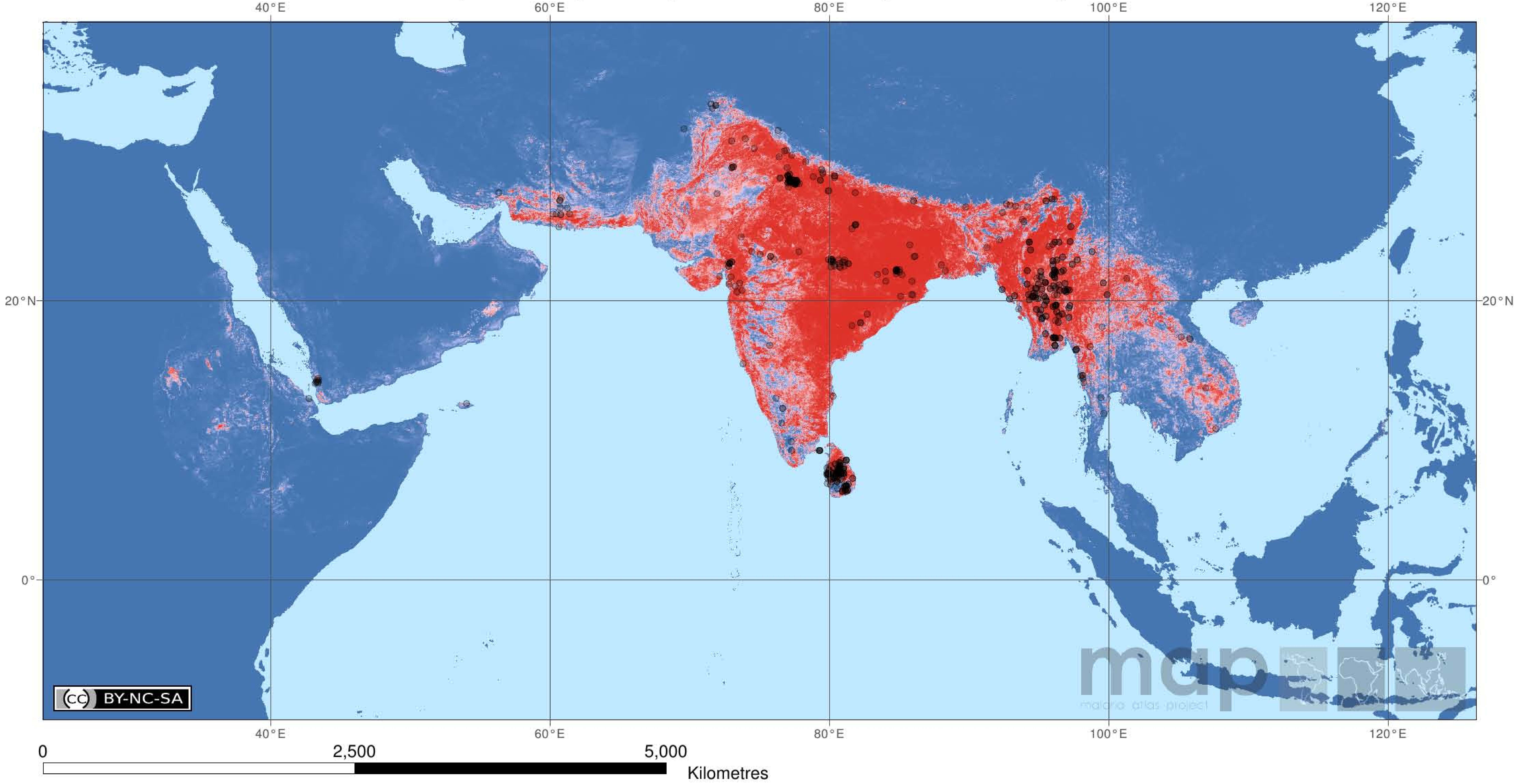

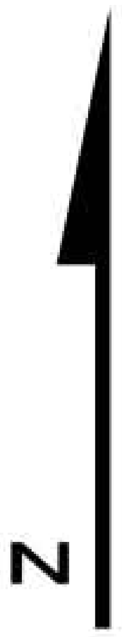

Mapping details: This map shows the predicted probability of occurrence of *An. culicifacies* in Asia-Pacific. The map was created with the Boosted Regression Trees (BRT) technique using 550 occurrence points, 500 pseudo occurrence points generated from a stratified random sample within the expert opinion range (see inset), balanced by 5,500 pseudo absence points sampled within a 1,500 km buffer outside the expert opinion range. The pseudo presence data were given half the weight of observed occurrence data. Predictions are not shown beyond the 1,500 km buffer. The black dots show 550 records of occurrence for *An. culicifacies* as detailed in Hay *et al.* [1].

Map statistics: Deviance=0.1857, Correlation=0.8666, Discrimination (AUC)=0.9769, Kappa=0.8317.

Environmental variables used: 1. NDVI (P1), 2. Prec (A2), 3. LST (P1), 4. Prec (P1) and 5. NDVI (min). Please see additional file 2 for abbreviations and definitions.

Copyright: Licensed to the Malaria Atlas Project (MAP; www.map.ox.ac.uk) under a Creative Commons Attribution 3.0 License (http://creativecommons.org/).

Citation: Sinka *et al.* (2011). The dominant *Anopheles* vectors of human malaria in the Asia-Pacific region: occurrence data, distribution maps and bionomic précis. Parasites & Vectors, 4: 89.

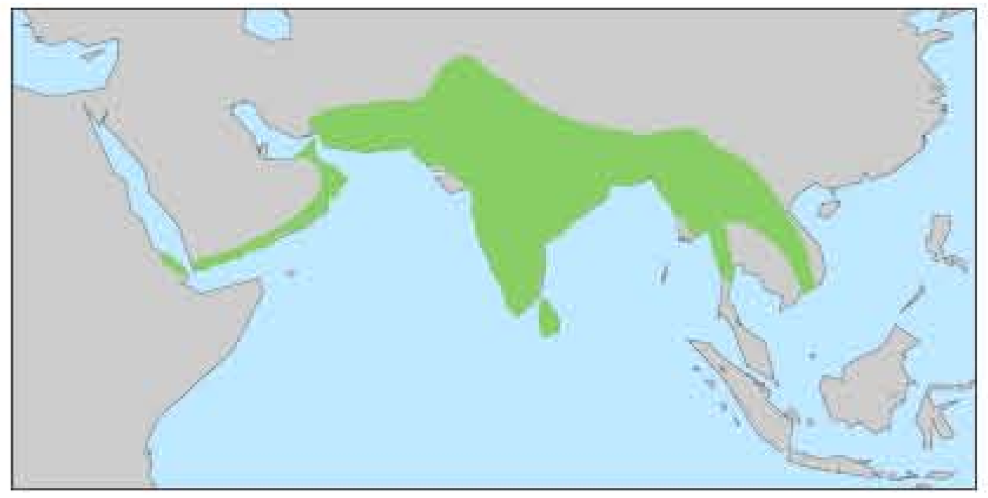

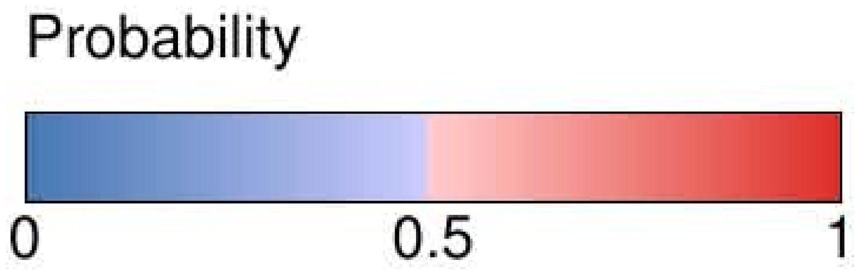

Anopheles (*Cellia*) *dirus* species complex

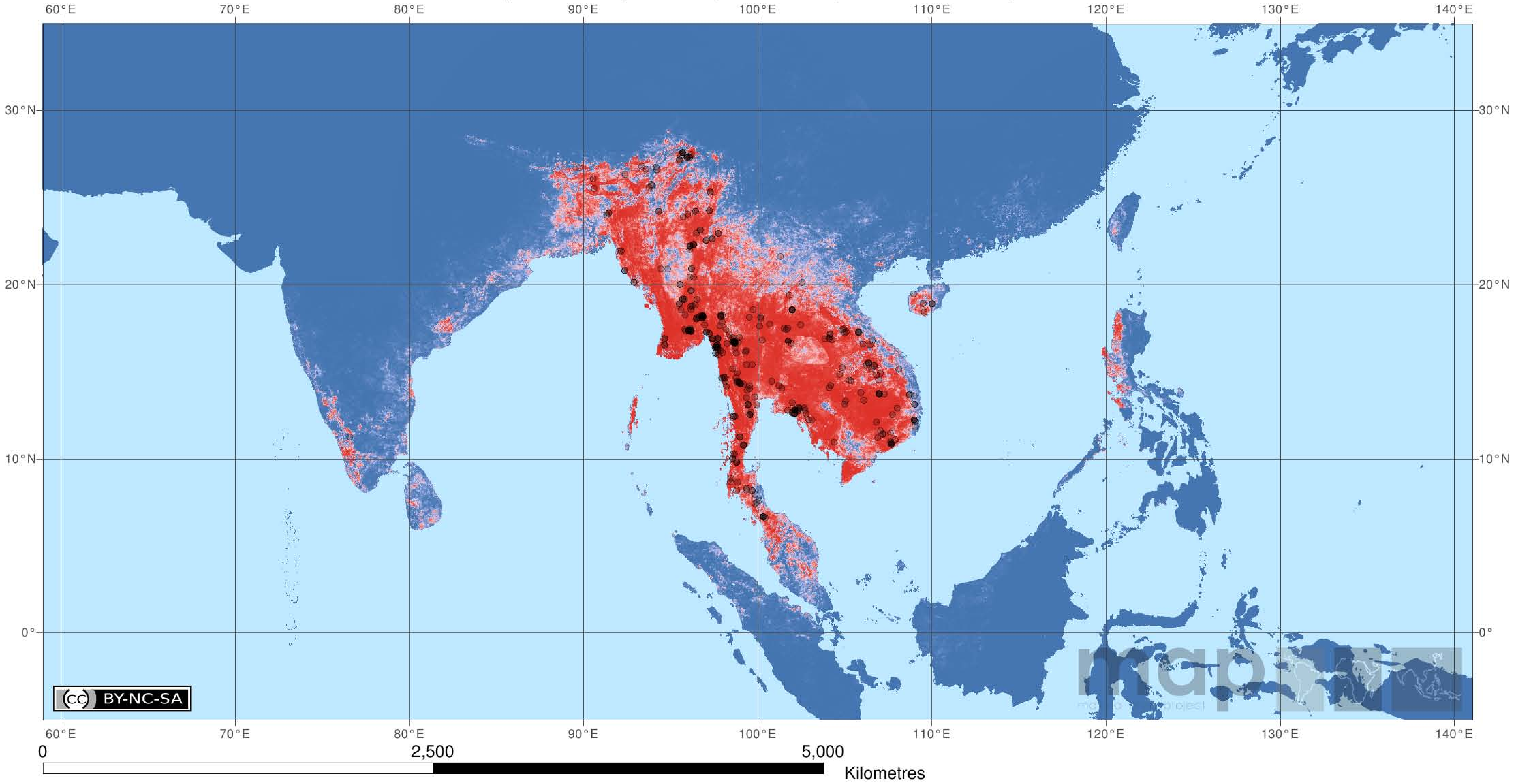

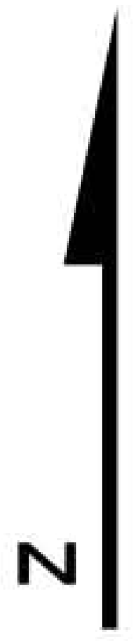

Mapping details: This map shows the predicted probability of occurrence of *An. dirus* in Asia-Pacific. The map was created with the Boosted Regression Trees (BRT) technique using 372 occurrence points, 500 pseudo occurrence points generated from a stratified random sample within the expert opinion range (see inset), balanced by 3,720 pseudo absence points sampled within a 1,500 km buffer outside the expert opinion range. The pseudo presence data were given half the weight of observed occurrence data. Predictions are not shown beyond the 1,500 km buffer. The black dots show 372 records of occurrence for *An. dirus* as detailed in Hay *et al.* [1].

Map statistics: Deviance=0.1738, Correlation=0.8793, Discrimination (AUC)=0.9857, Kappa=0.8451.

Environmental variables used: 1. LST (P1), 2. Prec (A1), 3. MIR (P1), 4. NDVI (mean) and 5. LST (P2). Please see additional file 2 for abbreviations and definitions.

Copyright: Licensed to the Malaria Atlas Project (MAP; www.map.ox.ac.uk) under a Creative Commons Attribution 3.0 License (http://creativecommons.org/).

Citation: Sinka *et al.* (2011). The dominant *Anopheles* vectors of human malaria in the Asia-Pacific region: occurrence data, distribution maps and bionomic précis. Parasites & Vectors, 4: 89.

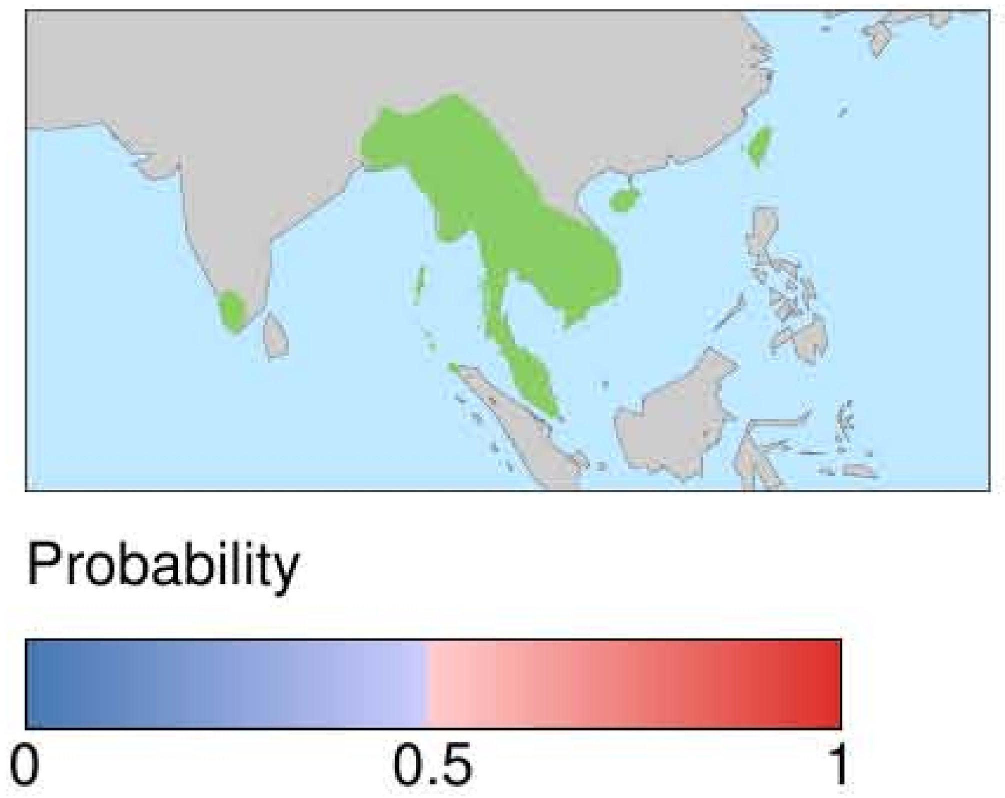

Anopheles (*Cellia*) *farauti* species complex

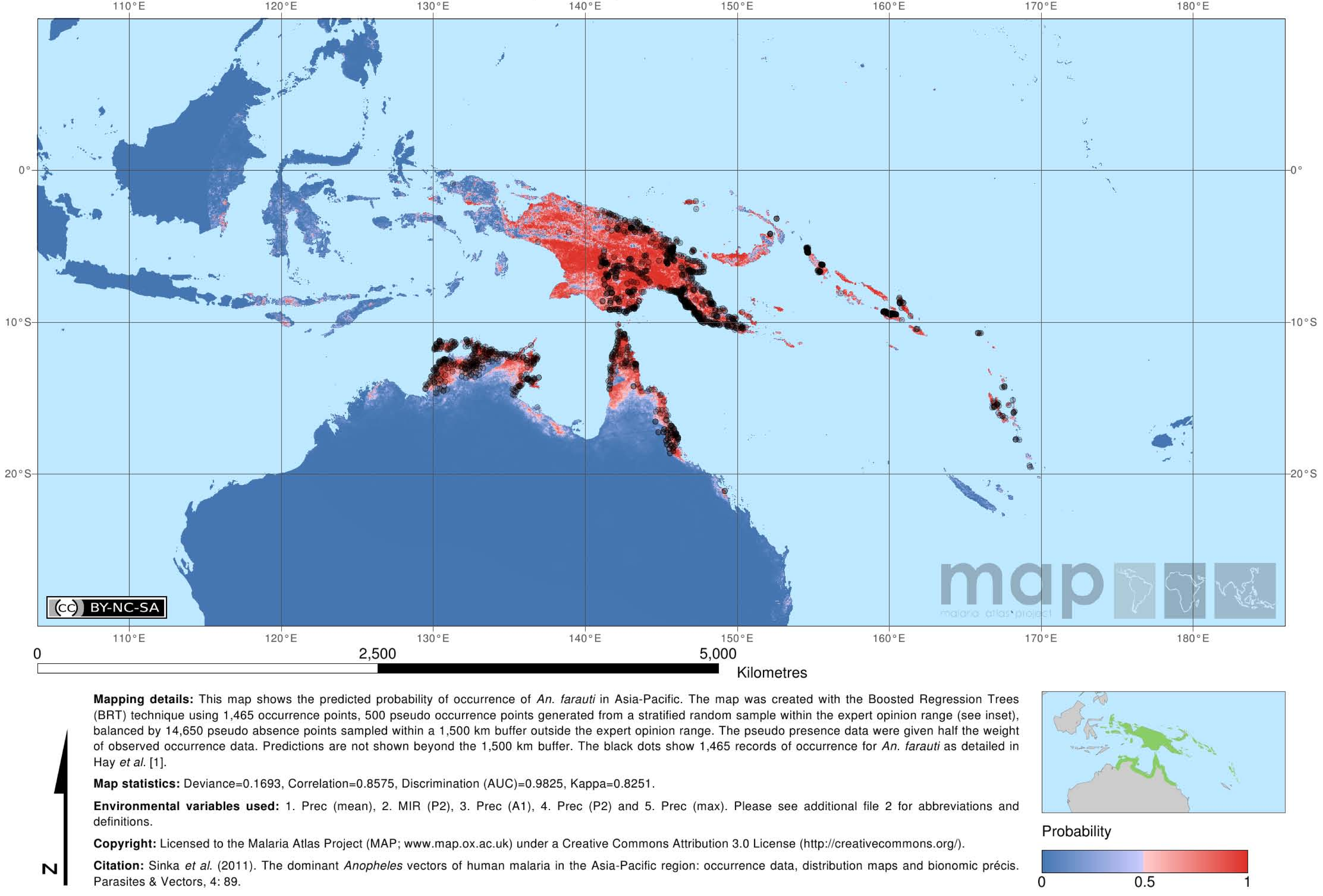

Mapping details: This map shows the predicted probability of occurrence of *An. farauti* in Asia-Pacific. The map was created with the Boosted Regression Trees (BRT) technique using 1,465 occurrence points, 500 pseudo occurrence points generated from a stratified random sample within the expert opinion range (see inset), balanced by 14,650 pseudo absence points sampled within a 1,500 km buffer outside the expert opinion range. The pseudo presence data were given half the weight of observed occurrence data. Predictions are not shown beyond the 1,500 km buffer. The black dots show 1,465 records of occurrence for *An. farauti* as detailed in Hay *et al.* [1].

Map statistics: Deviance=0.1693, Correlation=0.8575, Discrimination (AUC)=0.9825, Kappa=0.8251.

Environmental variables used: 1. Prec (mean), 2. MIR (P2), 3. Prec (A1), 4. Prec (P2) and 5. Prec (max). Please see additional file 2 for abbreviations and definitions.

Copyright: Licensed to the Malaria Atlas Project (MAP; www.map.ox.ac.uk) under a Creative Commons Attribution 3.0 License (http://creativecommons.org/).

Citation: Sinka *et al.* (2011). The dominant *Anopheles* vectors of human malaria in the Asia-Pacific region: occurrence data, distribution maps and bionomic précis. Parasites & Vectors, 4: 89.

Anopheles (*Cellia*) *flavirostris* (Ludlow, 1914)

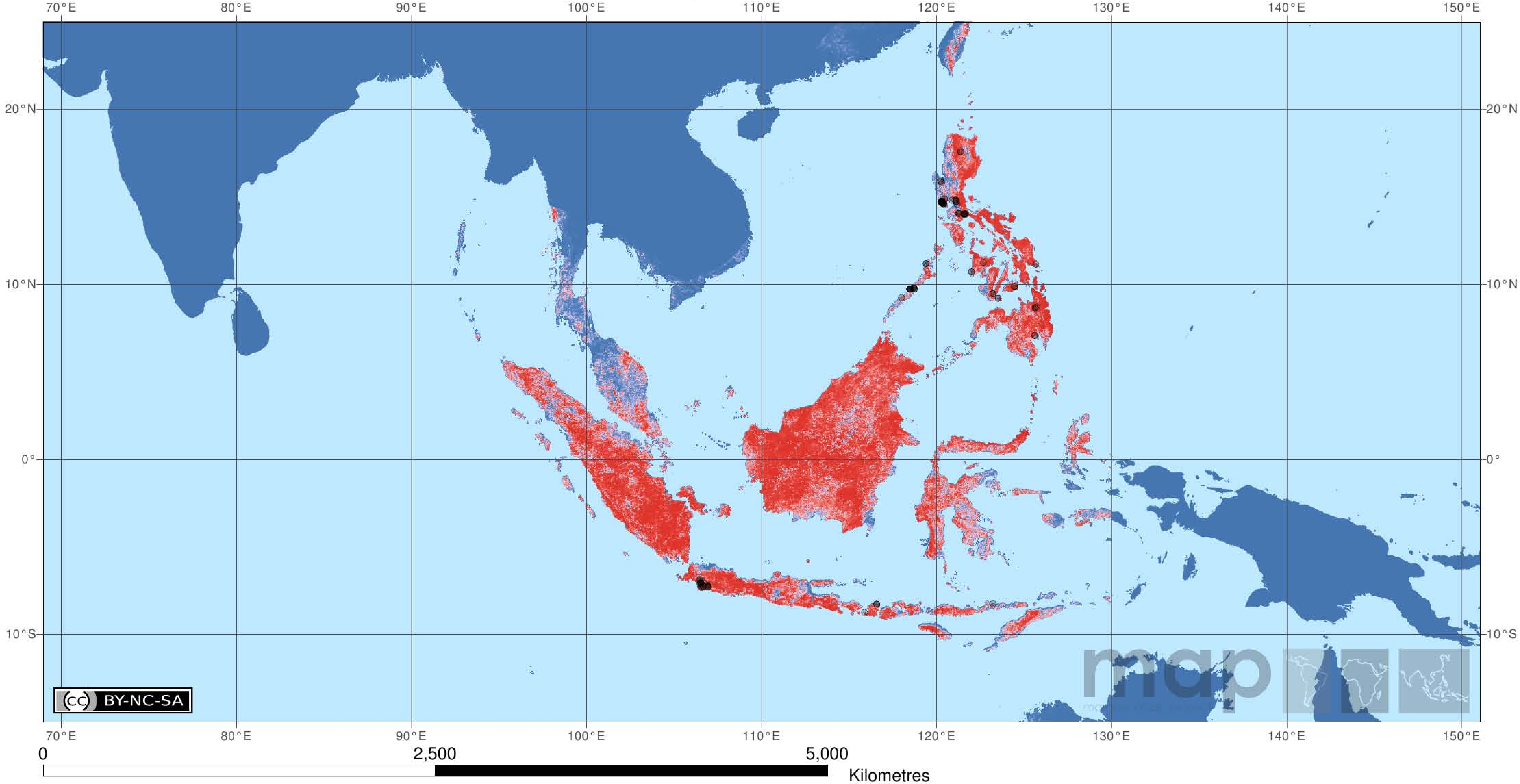

Mapping details: This map shows the predicted probability of occurrence of *An. flavirostris* in Asia-Pacific. The map was created with the Boosted Regression Trees (BRT) technique using 103 occurrence points, 500 pseudo occurrence points generated from a stratified random sample within the expert opinion range (see inset), balanced by 1,030 pseudo absence points sampled within a 1,500 km buffer outside the expert opinion range. The pseudo presence data were given half the weight of observed occurrence data. Predictions are not shown beyond the 1,500 km buffer. The black dots show 103 records of occurrence for *An. flavirostris* as detailed in Hay *et al.* [1].

Map statistics: Deviance=0.3328, Correlation=0.8442, Discrimination (AUC)=0.9685, Kappa=0.7955.

Environmental variables used: 1. Prec (mean), 2. Prec (P2), 3. GLOB (40), 4. Prec (min) and 5. Prec (P1). Please see additional file 2 for abbreviations and definitions.

Copyright: Licensed to the Malaria Atlas Project (MAP; www.map.ox.ac.uk) under a Creative Commons Attribution 3.0 License (http://creativecommons.org/).

Citation: Sinka *et al.* (2011). The dominant *Anopheles* vectors of human malaria in the Asia-Pacific region: occurrence data, distribution maps and bionomic précis. Parasites & Vectors, 4: 89.

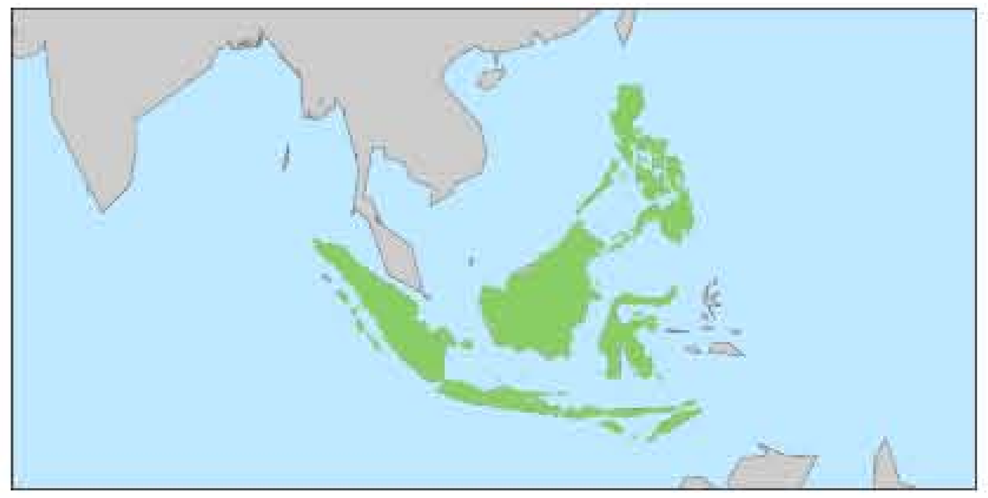

Probability

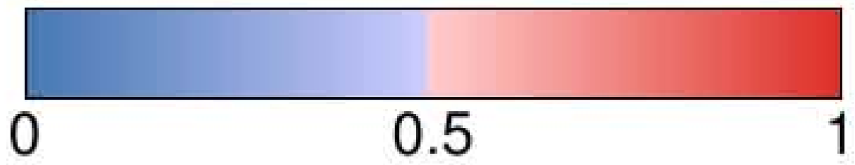

N

Anopheles (*Cellia*) *fluviatilis* species complex

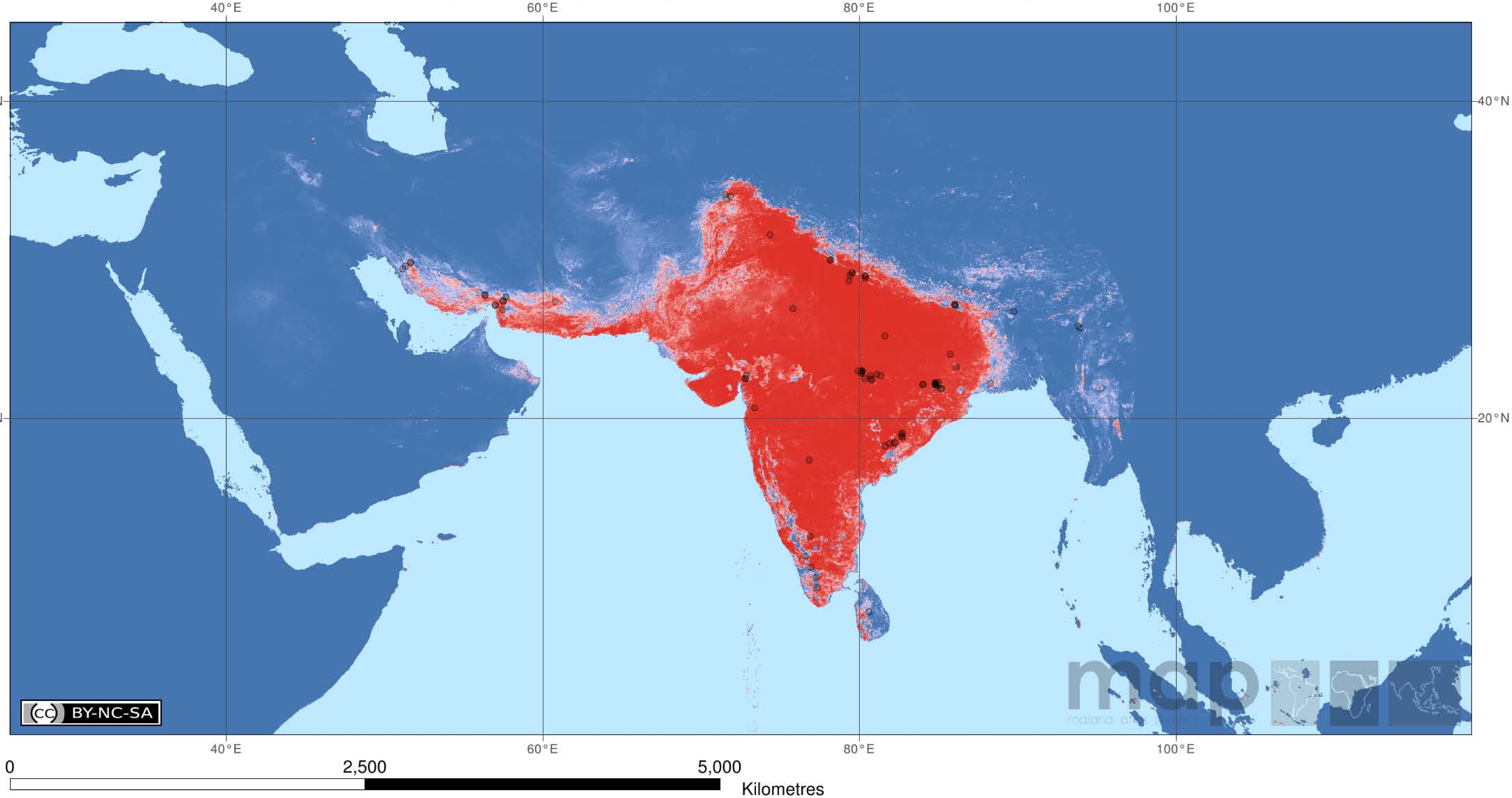

Mapping details: This map shows the predicted probability of occurrence of *An. fluviatilis* in Asia-Pacific. The map was created with the Boosted Regression Trees (BRT) technique using 83 occurrence points, 500 pseudo occurrence points generated from a stratified random sample within the expert opinion range (see inset), balanced by 830 pseudo absence points sampled within a 1,500 km buffer outside the expert opinion range. The pseudo presence data were given half the weight of observed occurrence data. Predictions are not shown beyond the 1,500 km buffer. The black dots show 83 records of occurrence for *An. fluviatilis* as detailed in Hay *et al.* [1].

Map statistics: Deviance=0.2021, Correlation=0.8994, Discrimination (AUC)=0.9797, Kappa=0.8689.

Environmental variables used: 1. Prec (P1), 2. LST (P1), 3. LST (P2), 4. Prec (A2) and 5. NDVI (P1). Please see additional file 2 for abbreviations and definitions.

Copyright: Licensed to the Malaria Atlas Project (MAP; www.map.ox.ac.uk) under a Creative Commons Attribution 3.0 License (http://creativecommons.org/).

Citation: Sinka *et al.* (2011). The dominant *Anopheles* vectors of human malaria in the Asia-Pacific region: occurrence data, distribution maps and bionomic précis. Parasites & Vectors, 4: 89.

N

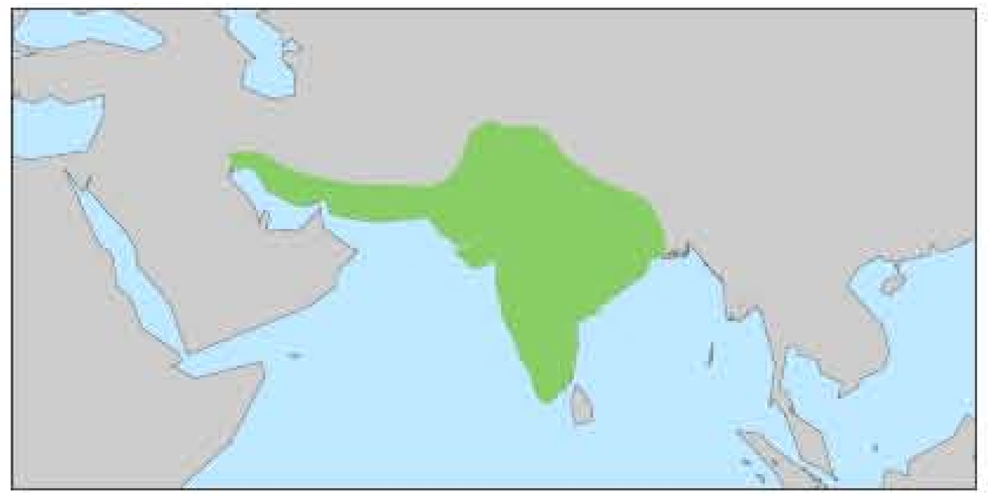

Probability

0 0.5 1

Anopheles (*Cellia*) *koliensis* Owen, 1945

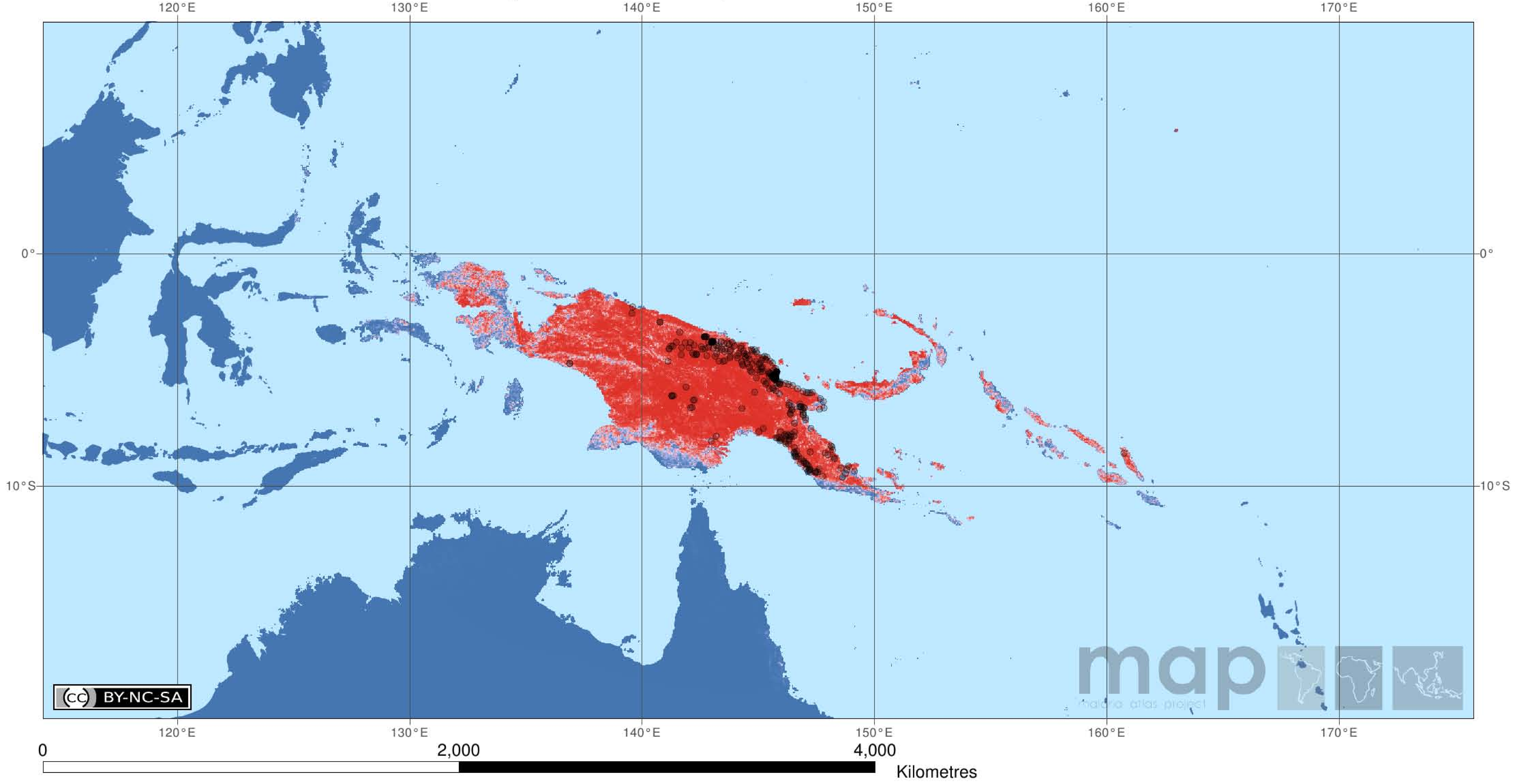

Mapping details: This map shows the predicted probability of occurrence of *An. koliensis* in Asia-Pacific. The map was created with the Boosted Regression Trees (BRT) technique using 325 occurrence points, 500 pseudo occurrence points generated from a stratified random sample within the expert opinion range (see inset), balanced by 3,250 pseudo absence points sampled within a 1,500 km buffer outside the expert opinion range. The pseudo presence data were given half the weight of observed occurrence data. Predictions are not shown beyond the 1,500 km buffer. The black dots show 325 records of occurrence for *An. koliensis* as detailed in Hay *et al.* [1].

Map statistics: Deviance=0.1339, Correlation=0.9218, Discrimination (AUC)=0.9933, Kappa=0.9046.

Environmental variables used: 1. Prec (mean), 2. LST (max), 3. Prec (P2), 4. LST (mean) and 5. Prec (min). Please see additional file 2 for abbreviations and definitions.

Copyright: Licensed to the Malaria Atlas Project (MAP; www.map.ox.ac.uk) under a Creative Commons Attribution 3.0 License (http://creativecommons.org/).

Citation: Sinka *et al.* (2011). The dominant *Anopheles* vectors of human malaria in the Asia-Pacific region: occurrence data, distribution maps and bionomic précis. Parasites & Vectors, 4: 89.

N

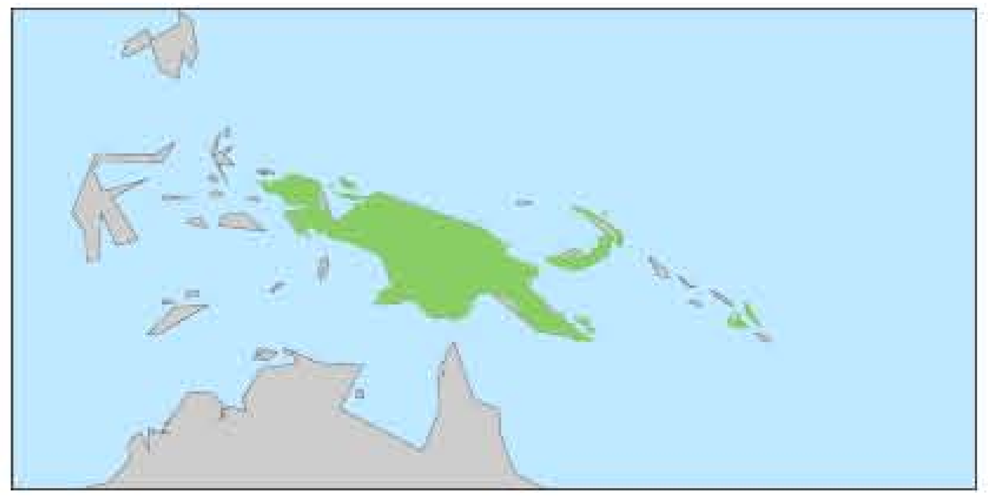

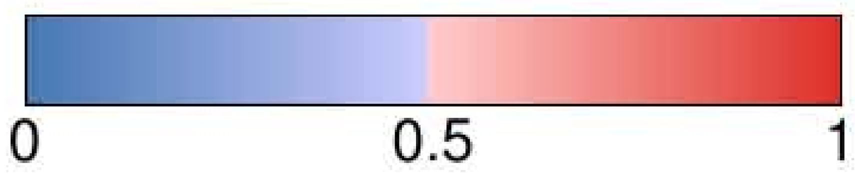

Anopheles (*Anopheles*) *lesteri* Baisas & Hu, 1936 (formerly *An. anthropophagus* in China)

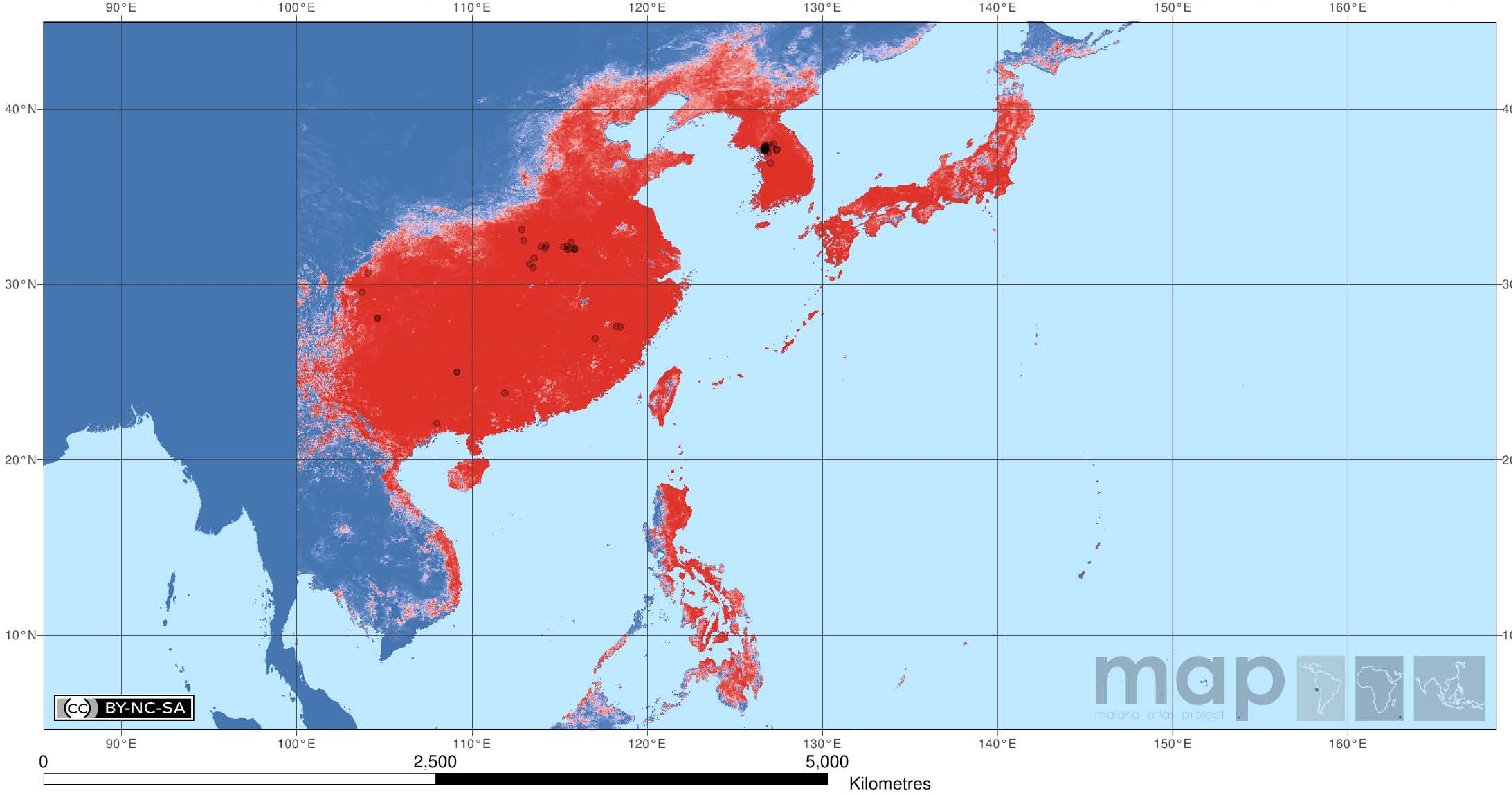

Mapping details: This map shows the predicted probability of occurrence of *An. lesteri* in Asia-Pacific. The map was created with the Boosted Regression Trees (BRT) technique using 47 occurrence points, 500 pseudo occurrence points generated from a stratified random sample within the expert opinion range (see inset), balanced by 470 pseudo absence points sampled within a 1,500 km buffer outside the expert opinion range. The pseudo presence data were given half the weight of observed occurrence data. Predictions are not shown beyond the 1,500 km buffer. The black dots show 47 records of occurrence for *An. lesteri* as detailed in Hay *et al.* [1].

Map statistics: Deviance=0.192, Correlation=0.9176, Discrimination (AUC)=0.9897, Kappa=0.8853.

Environmental variables used: 1. Prec (min), 2. LST (min), 3. LST (A1), 4. LST (mean) and 5. LST (P2). Please see additional file 2 for abbreviations and definitions.

Copyright: Licensed to the Malaria Atlas Project (MAP; www.map.ox.ac.uk) under a Creative Commons Attribution 3.0 License (http://creativecommons.org/).

Citation: Sinka *et al.* (2011). The dominant *Anopheles* vectors of human malaria in the Asia-Pacific region: occurrence data, distribution maps and bionomic précis. Parasites & Vectors, 4: 89.

N

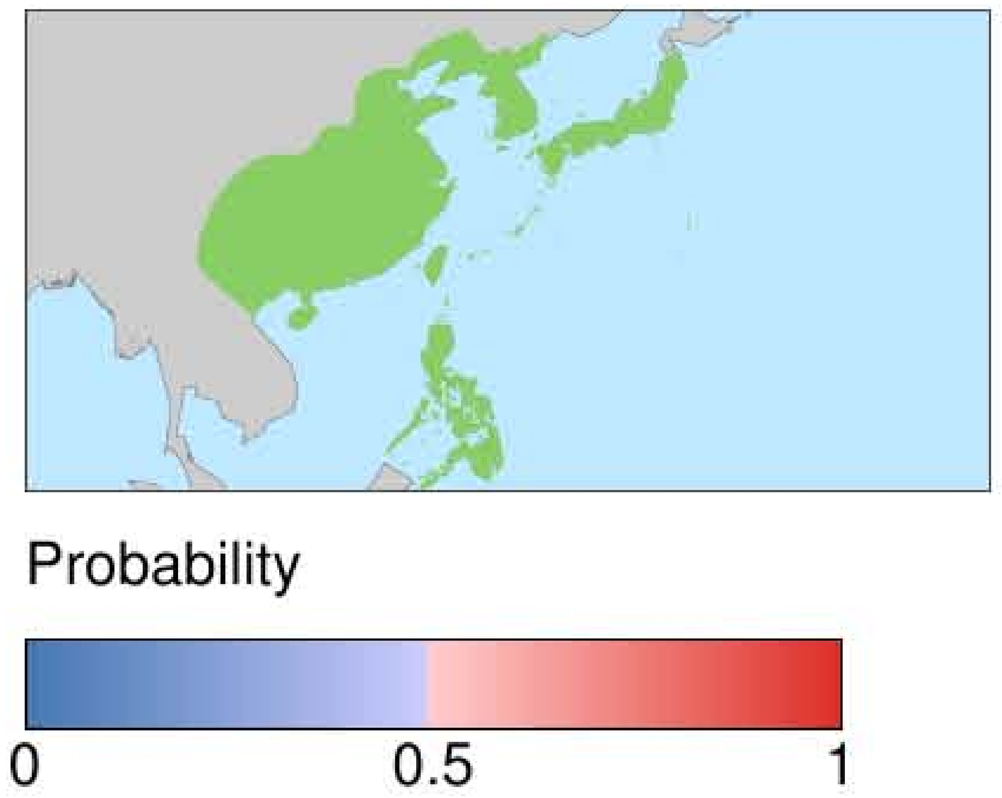

Anopheles (*Cellia*) *leucosphyrus* and *Anopheles* (*Cellia*) *latens*

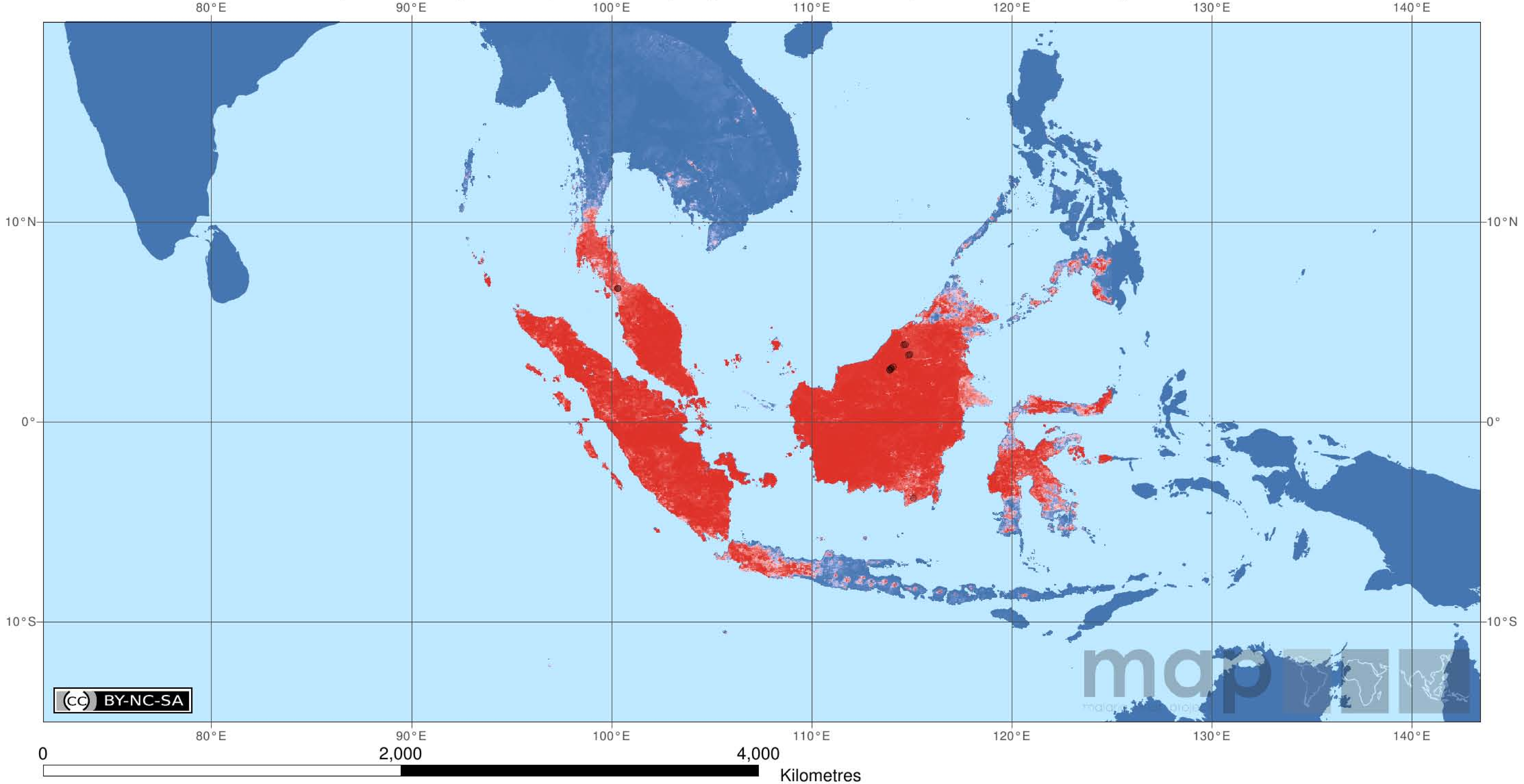

Mapping details: This map shows the predicted probability of occurrence of *Anopheles leucosphyrus* and *Anopheles latens* in Asia-Pacific. The map was created with the Boosted Regression Trees (BRT) technique using 12 occurrence points, 500 pseudo occurrence points generated from a stratified random sample within the expert opinion range (see inset), balanced by 120 pseudo absence points sampled within a 1,500 km buffer outside the expert opinion range. The pseudo presence data were given half the weight of observed occurrence data. Predictions are not shown beyond the 1,500 km buffer. The black dots show 12 records of occurrence for *An. leucosphyrus* as detailed in Hay *et al.* [1].

Map statistics: Deviance=0.1735, Correlation=0.9105, Discrimination (AUC)=0.9786, Kappa=0.9047.

Environmental variables used: 1. LST (mean), 2. LST (max), 3. LST (P2), 4. Prec (max) and 5. Prec (min). Please see additional file 2 for abbreviations and definitions.

Copyright: Licensed to the Malaria Atlas Project (MAP; www.map.ox.ac.uk) under a Creative Commons Attribution 3.0 License (http://creativecommons.org/).

Citation: Sinka *et al.* (2011). The dominant *Anopheles* vectors of human malaria in the Asia-Pacific region: occurrence data, distribution maps and bionomic précis. Parasites & Vectors, 4: 89.

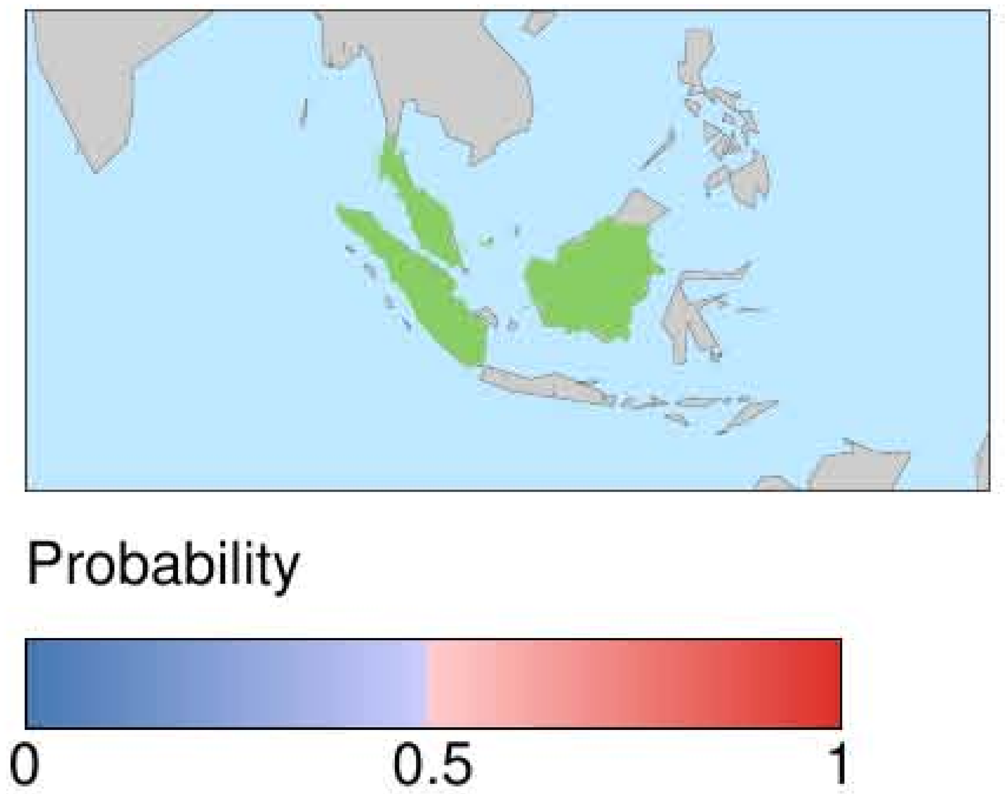

N

Anopheles (*Cellia*) *maculatus* Group

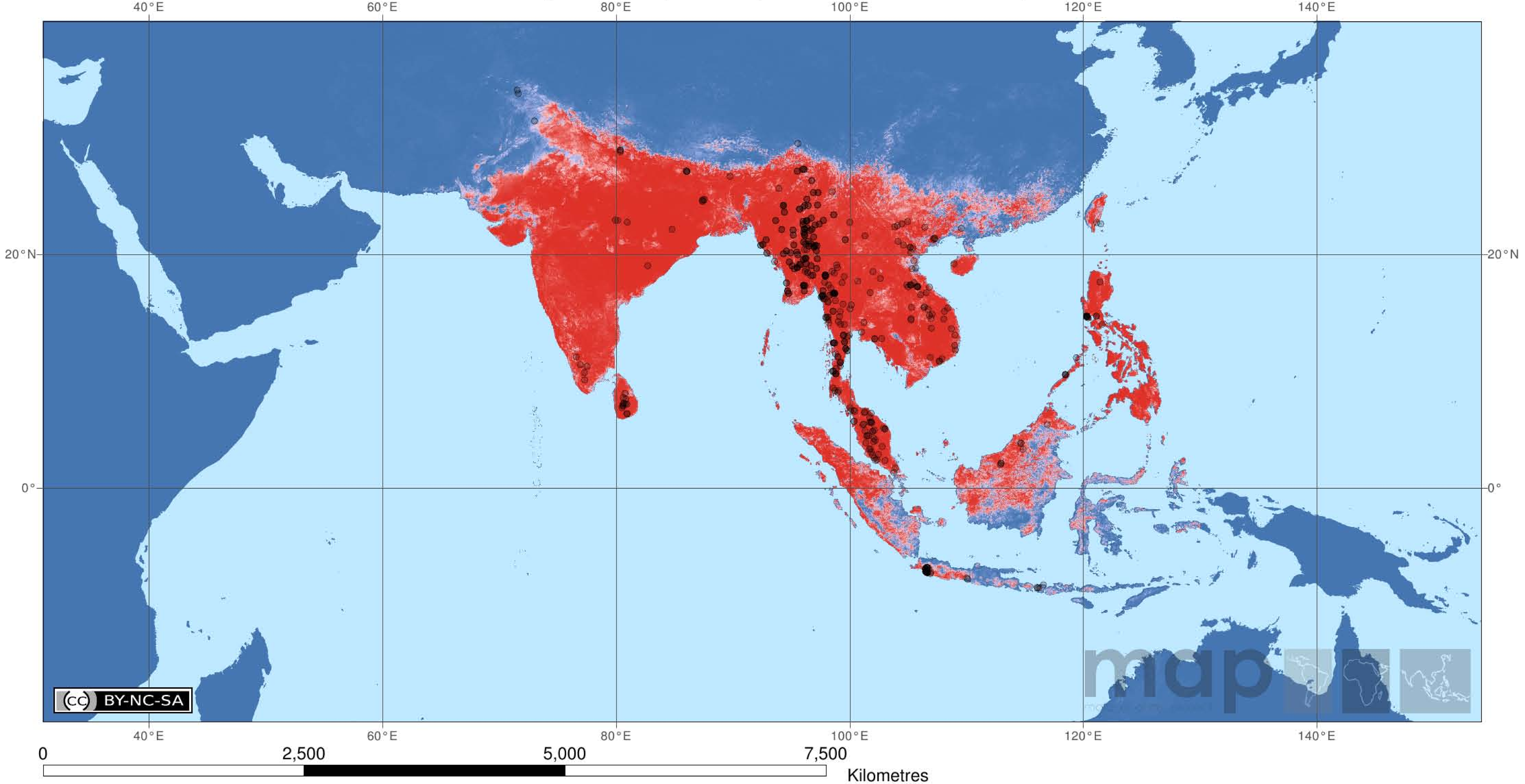

Mapping details: This map shows the predicted probability of occurrence of *An. maculatus* in Asia-Pacific. The map was created with the Boosted Regression Trees (BRT) technique using 471 occurrence points, 500 pseudo occurrence points generated from a stratified random sample within the expert opinion range (see inset), balanced by 4,710 pseudo absence points sampled within a 1,500 km buffer outside the expert opinion range. The pseudo presence data were given half the weight of observed occurrence data. Predictions are not shown beyond the 1,500 km buffer. The black dots show 471 records of occurrence for *An. maculatus* as detailed in Hay *et al.* [1].

Map statistics: Deviance=0.1565, Correlation=0.8935, Discrimination (AUC)=0.9785, Kappa=0.8683.

Environmental variables used: 1. LST (P1), 2. MIR (P1), 3. Prec (max), 4. Prec (P1) and 5. NDVI (mean). Please see additional file 2 for abbreviations and definitions.

Copyright: Licensed to the Malaria Atlas Project (MAP; www.map.ox.ac.uk) under a Creative Commons Attribution 3.0 License (http://creativecommons.org/).

Citation: Sinka *et al.* (2011). The dominant *Anopheles* vectors of human malaria in the Asia-Pacific region: occurrence data, distribution maps and bionomic précis. Parasites & Vectors, 4: 89.

N

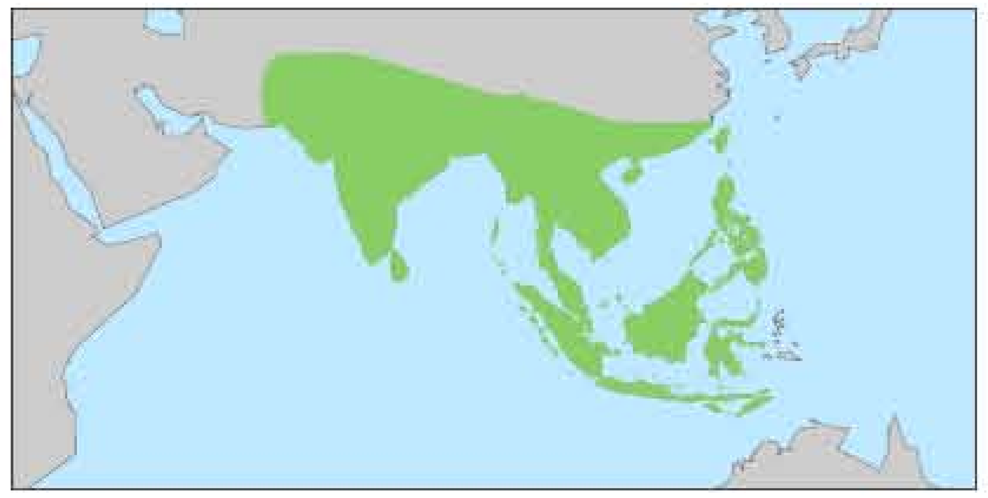

Probability

0 0.5 1

Anopheles (*Cellia*) *minimus* species complex

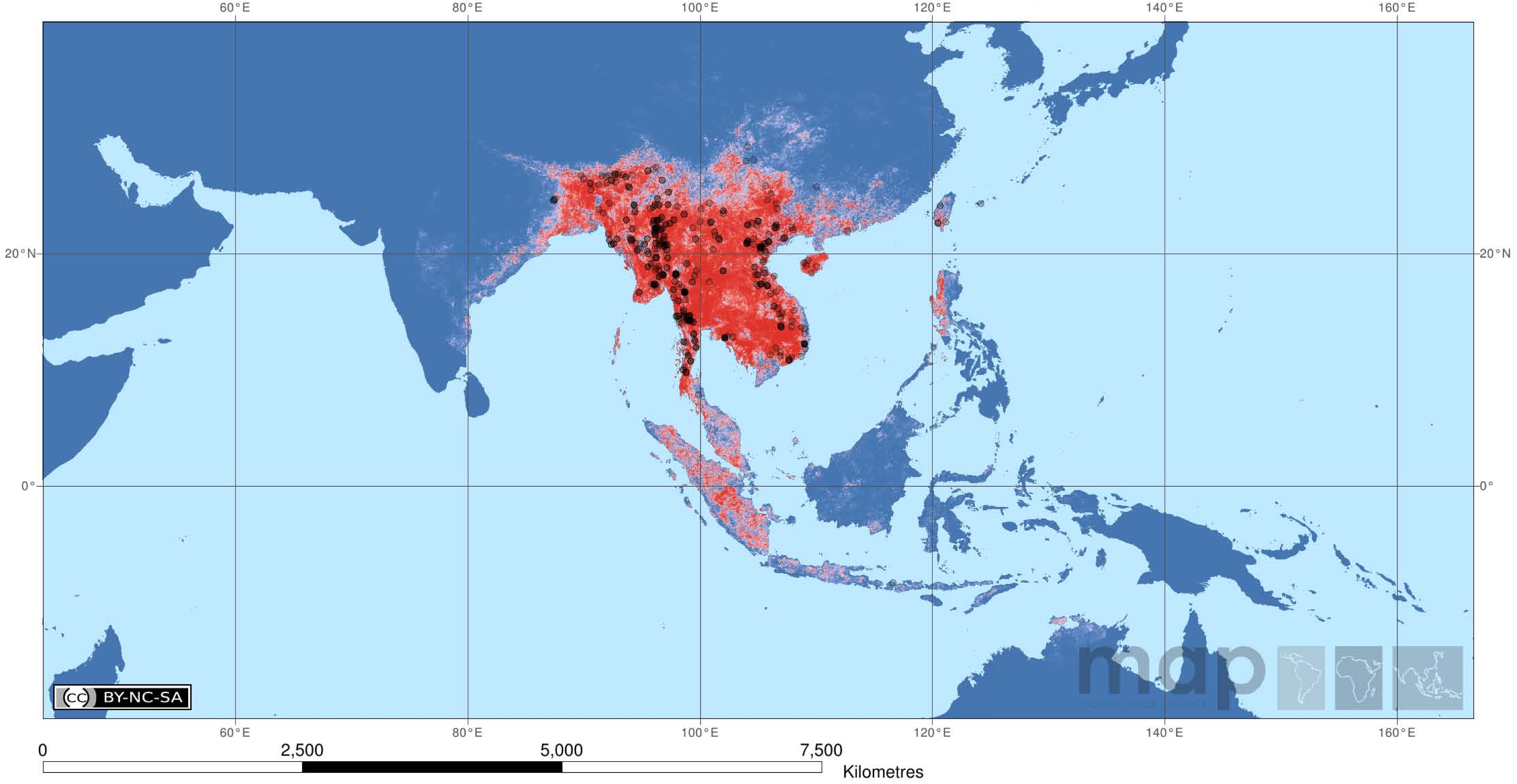

Mapping details: This map shows the predicted probability of occurrence of *An. minimus* in Asia-Pacific. The map was created with the Boosted Regression Trees (BRT) technique using 445 occurrence points, 500 pseudo occurrence points generated from a stratified random sample within the expert opinion range (see inset), balanced by 4,450 pseudo absence points sampled within a 1,500 km buffer outside the expert opinion range. The pseudo presence data were given half the weight of observed occurrence data. Predictions are not shown beyond the 1,500 km buffer. The black dots show 445 records of occurrence for *An. minimus* as detailed in Hay *et al.* [1].

Map statistics: Deviance=0.1927, Correlation=0.8562, Discrimination (AUC)=0.9793, Kappa=0.8124.

Environmental variables used: 1. LST (P1), 2. Prec (A1), 3. MIR (P1), 4. LST (mean) and 5. LST (P2). Please see additional file 2 for abbreviations and definitions.

Copyright: Licensed to the Malaria Atlas Project (MAP; www.map.ox.ac.uk) under a Creative Commons Attribution 3.0 License (http://creativecommons.org/).

Citation: Sinka *et al.* (2011). The dominant *Anopheles* vectors of human malaria in the Asia-Pacific region: occurrence data, distribution maps and bionomic précis. Parasites & Vectors, 4: 89.

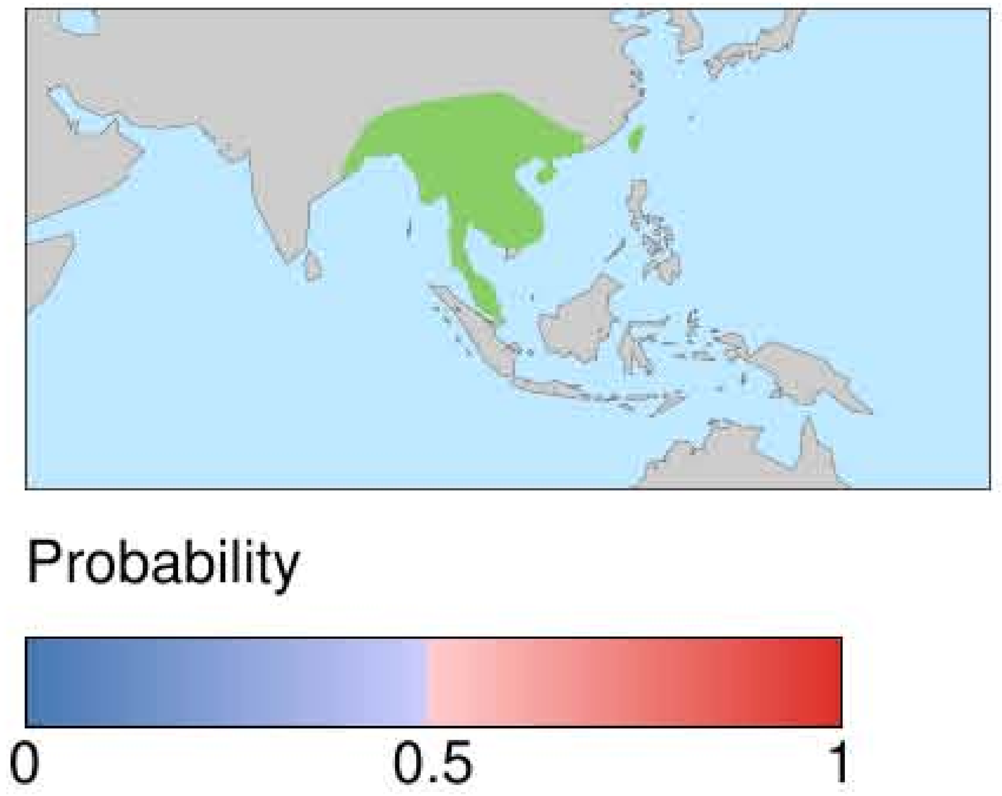

Anopheles (*Cellia*) *punctulatus* species complex

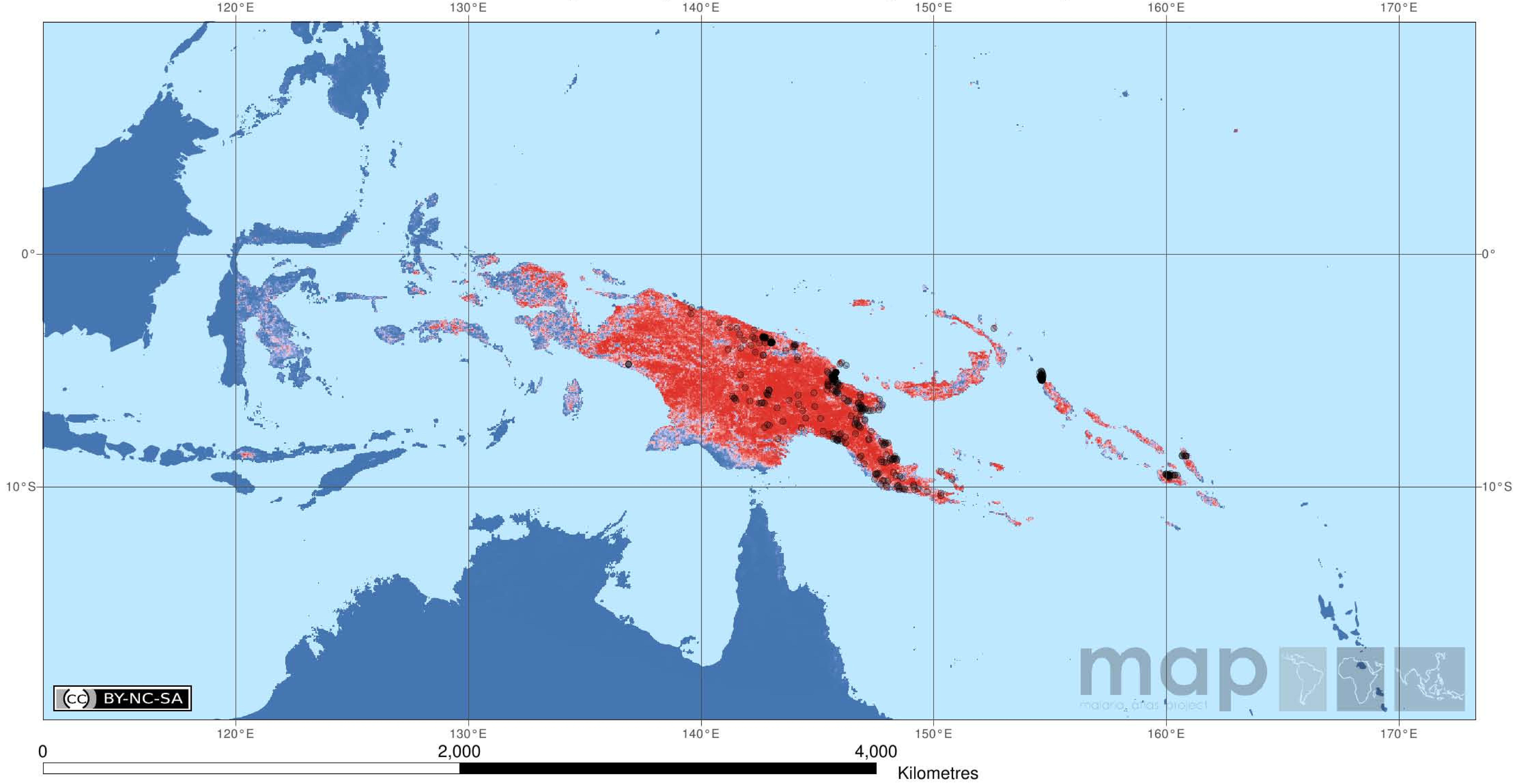

Mapping details: This map shows the predicted probability of occurrence of *An. punctulatus* in Asia-Pacific. The map was created with the Boosted Regression Trees (BRT) technique using 379 occurrence points, 500 pseudo occurrence points generated from a stratified random sample within the expert opinion range (see inset), balanced by 3,790 pseudo absence points sampled within a 1,500 km buffer outside the expert opinion range. The pseudo presence data were given half the weight of observed occurrence data. Predictions are not shown beyond the 1,500 km buffer. The black dots show 379 records of occurrence for *An. punctulatus* as detailed in Hay *et al.* [1].

Map statistics: Deviance=0.2033, Correlation=0.8668, Discrimination (AUC)=0.9815, Kappa=0.8323.

Environmental variables used: 1. LST (max), 2. NDVI (P1), 3. Prec (min), 4. MIR (mean) and 5. MIR (max). Please see additional file 2 for abbreviations and definitions.

Copyright: Licensed to the Malaria Atlas Project (MAP; www.map.ox.ac.uk) under a Creative Commons Attribution 3.0 License (http://creativecommons.org/).

Citation: Sinka *et al.* (2011). The dominant *Anopheles* vectors of human malaria in the Asia-Pacific region: occurrence data, distribution maps and bionomic précis. Parasites & Vectors, 4: 89.

N

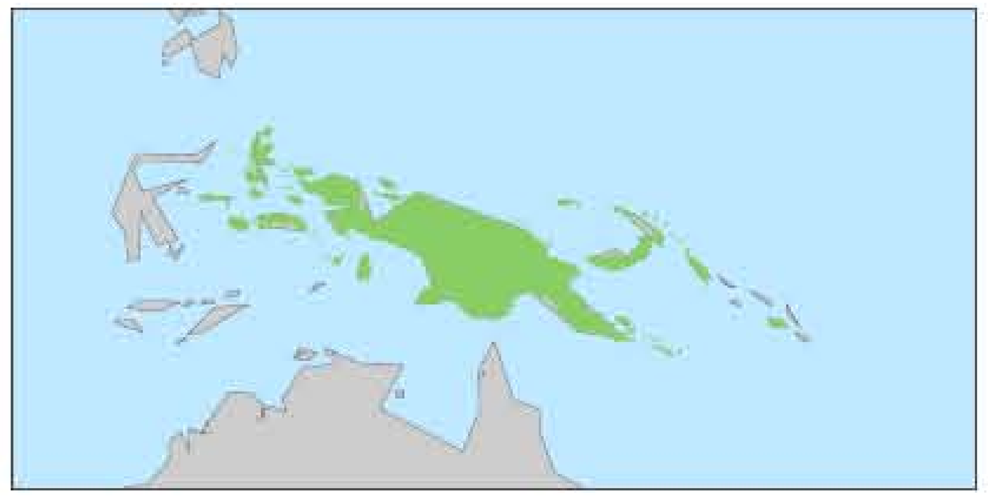

Probability

0 0.5 1

Anopheles (*Anopheles*) *sinensis* species complex

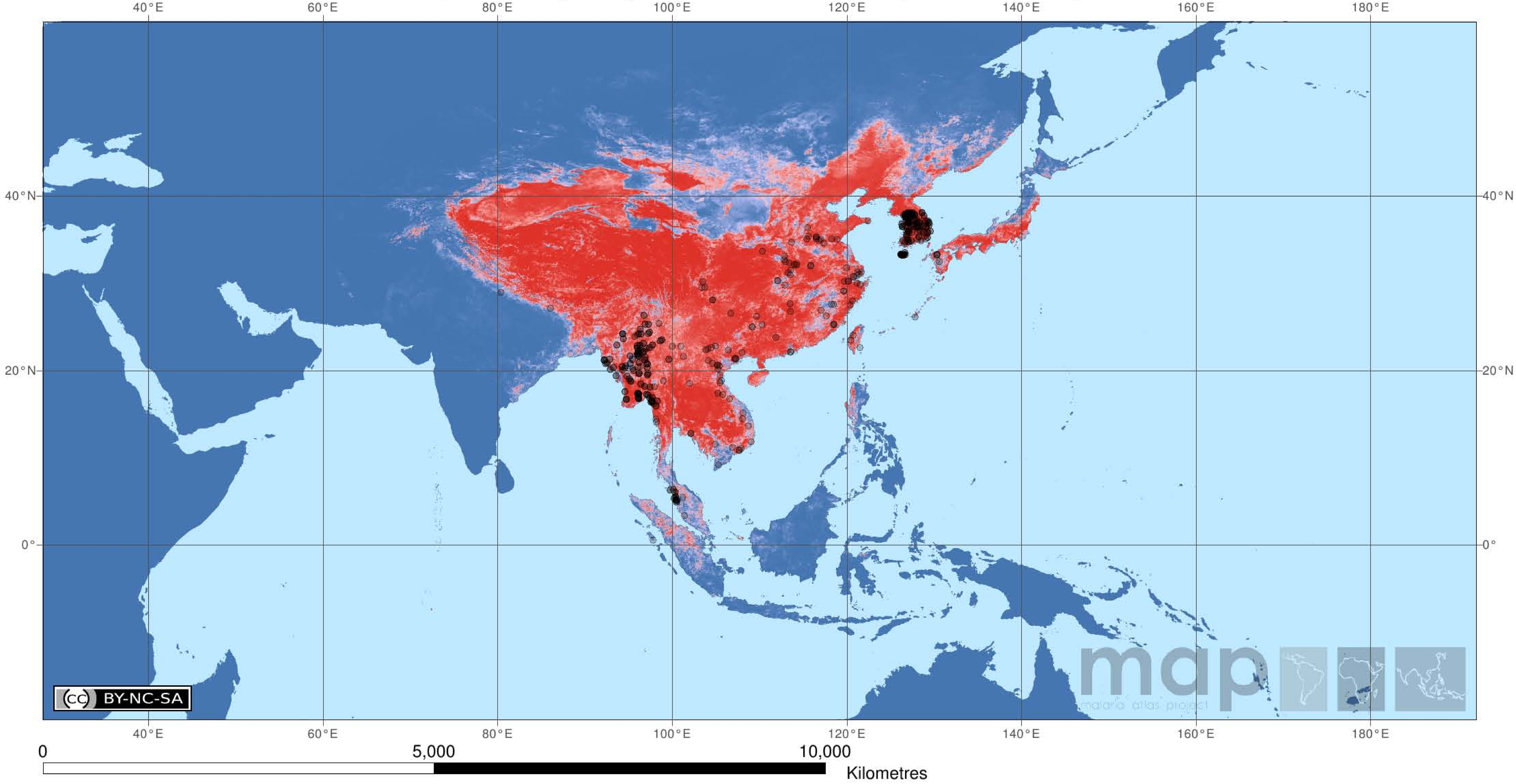

Mapping details: This map shows the predicted probability of occurrence of *An. sinensis* in Asia-Pacific. The map was created with the Boosted Regression Trees (BRT) technique using 568 occurrence points, 500 pseudo occurrence points generated from a stratified random sample within the expert opinion range (see inset), balanced by 5,680 pseudo absence points sampled within a 1,500 km buffer outside the expert opinion range. The pseudo presence data were given half the weight of observed occurrence data. Predictions are not shown beyond the 1,500 km buffer. The black dots show 568 records of occurrence for *An. sinensis* as detailed in Hay *et al.* [1].

Map statistics: Deviance=0.1608, Correlation=0.8835, Discrimination (AUC)=0.9806, Kappa=0.8508.

Environmental variables used: 1. Prec (mean), 2. DEM, 3. Prec (P1), 4. Prec (A1) and 5. LST (mean). Please see additional file 2 for abbreviations and definitions.

Copyright: Licensed to the Malaria Atlas Project (MAP; www.map.ox.ac.uk) under a Creative Commons Attribution 3.0 License (http://creativecommons.org/).

Citation: Sinka *et al.* (2011). The dominant *Anopheles* vectors of human malaria in the Asia-Pacific region: occurrence data, distribution maps and bionomic précis. Parasites & Vectors, 4: 89.

N

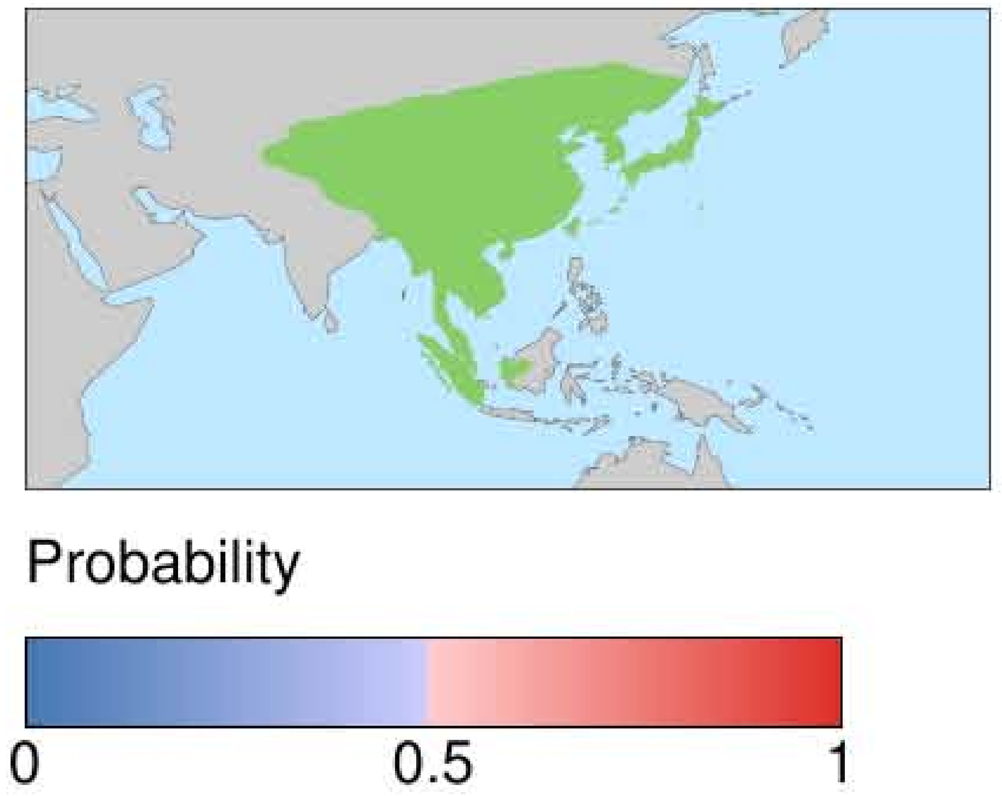

Anopheles (*Cellia*) *stephensi* Liston, 1901

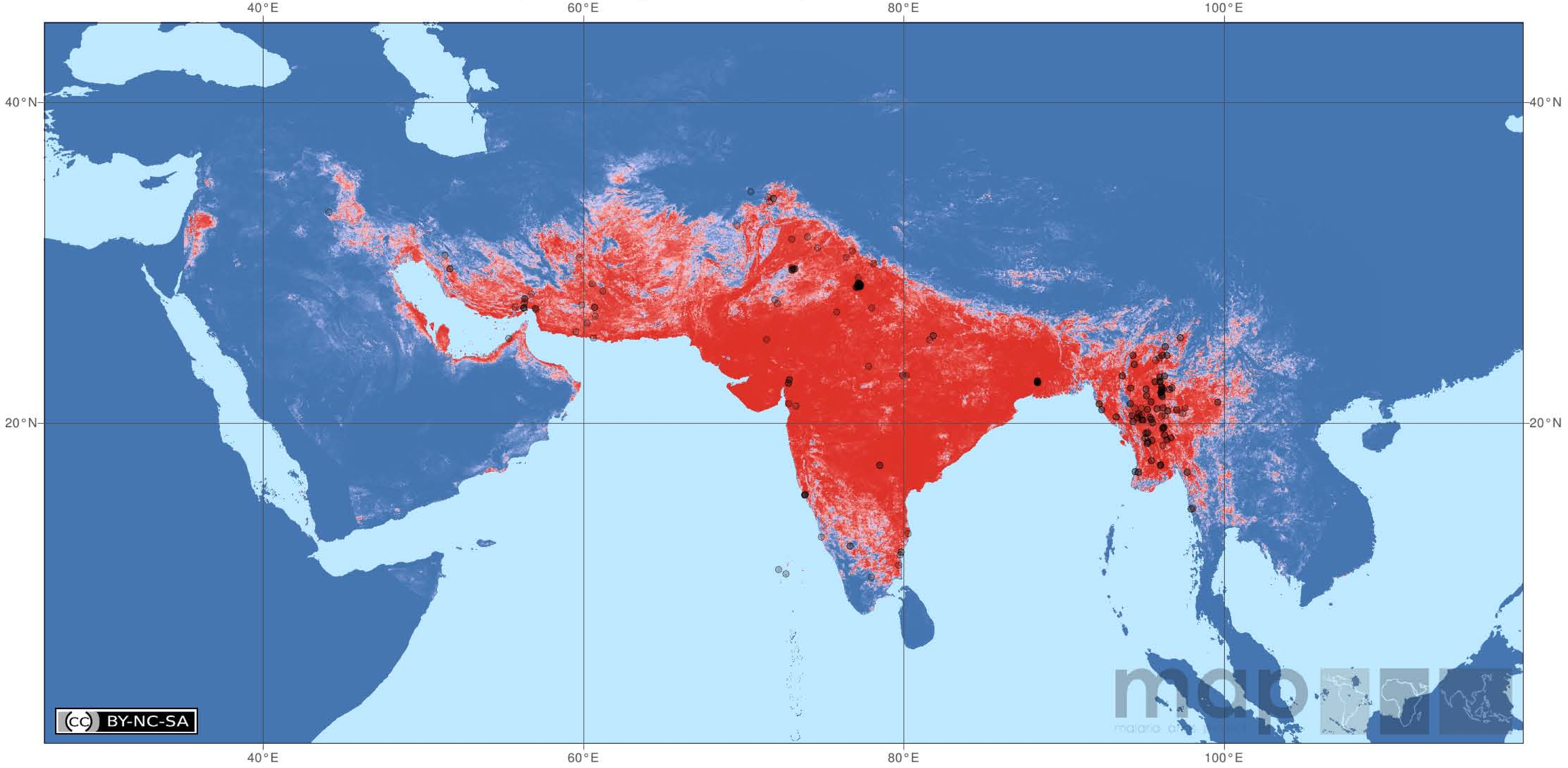

0 2,500 5,000
Kilometres

N

Mapping details: This map shows the predicted probability of occurrence of *An. stephensi* in Asia-Pacific. The map was created with the Boosted Regression Trees (BRT) technique using 261 occurrence points, 500 pseudo occurrence points generated from a stratified random sample within the expert opinion range (see inset), balanced by 2,610 pseudo absence points sampled within a 1,500 km buffer outside the expert opinion range. The pseudo presence data were given half the weight of observed occurrence data. Predictions are not shown beyond the 1,500 km buffer. The black dots show 261 records of occurrence for *An. stephensi* as detailed in Hay *et al.* [1].

Map statistics: Deviance=0.169, Correlation=0.9043, Discrimination (AUC)=0.9838, Kappa=0.8778.

Environmental variables used: 1. LST (P1), 2. NDVI (P1), 3. Prec (min), 4. Prec (P1) and 5. Prec (P2). Please see additional file 2 for abbreviations and definitions.

Copyright: Licensed to the Malaria Atlas Project (MAP; www.map.ox.ac.uk) under a Creative Commons Attribution 3.0 License (http://creativecommons.org/).

Citation: Sinka *et al.* (2011). The dominant *Anopheles* vectors of human malaria in the Asia-Pacific region: occurrence data, distribution maps and bionomic précis. Parasites & Vectors, 4: 89.

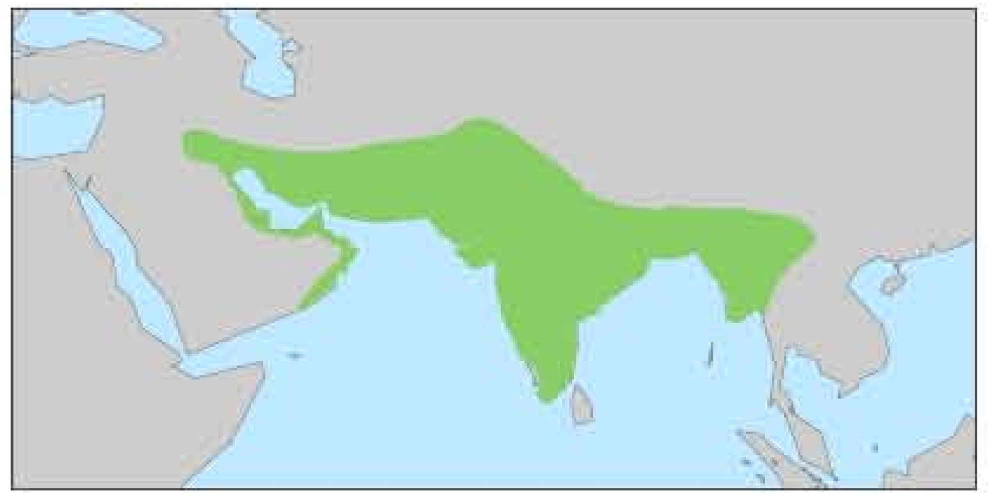

Probability

0 0.5 1

Anopheles (*Cellia*) *subpictus* species complex

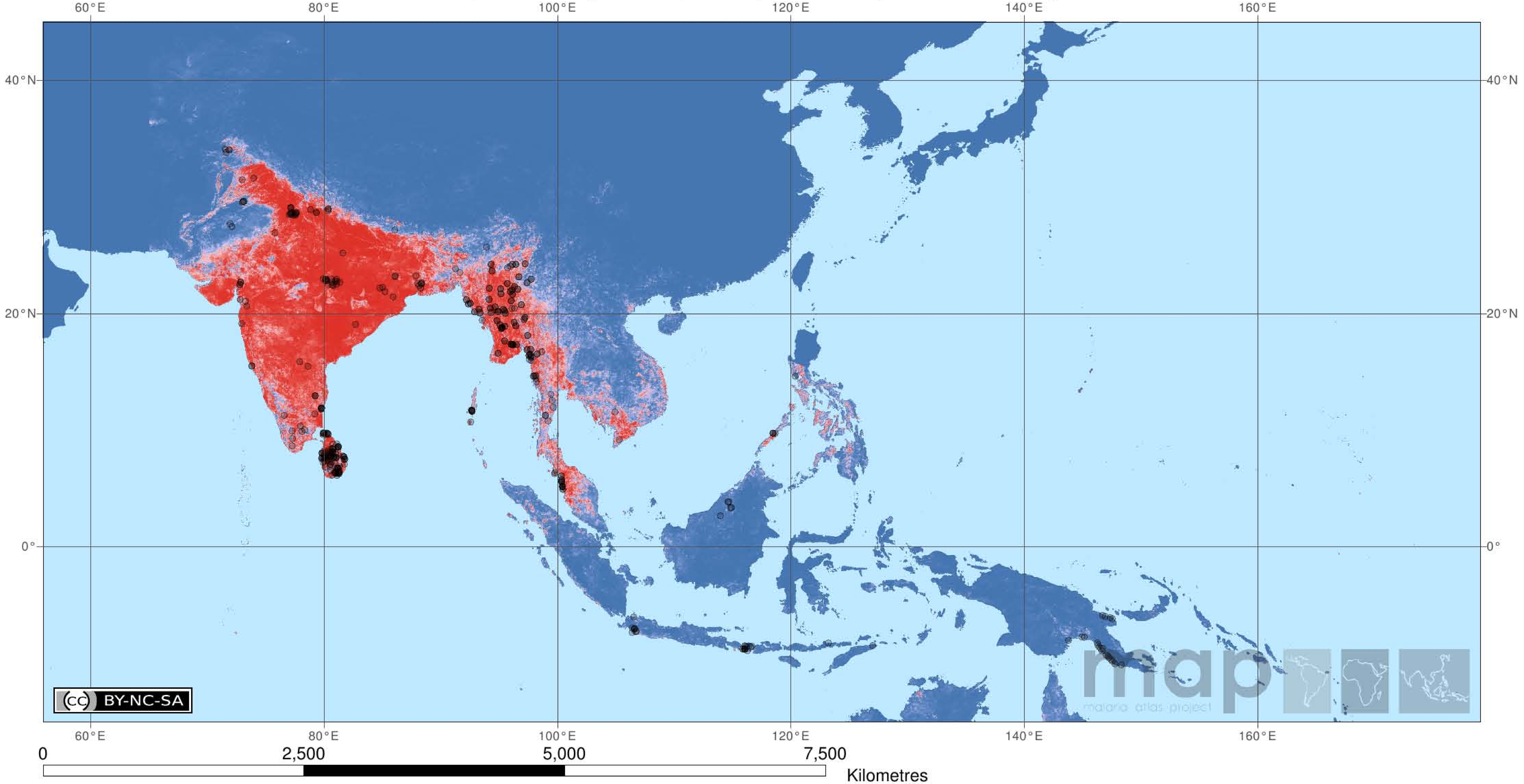

Mapping details: This map shows the predicted probability of occurrence of *An. subpictus* in Asia-Pacific. The map was created with the Boosted Regression Trees (BRT) technique using 410 occurrence points, 500 pseudo occurrence points generated from a stratified random sample within the expert opinion range (see inset), balanced by 4,100 pseudo absence points sampled within a 1,500 km buffer outside the expert opinion range. The pseudo presence data were given half the weight of observed occurrence data. Predictions are not shown beyond the 1,500 km buffer. The black dots show 410 records of occurrence for *An. subpictus* as detailed in Hay *et al.* [1].

Map statistics: Deviance=0.2812, Correlation=0.8186, Discrimination (AUC)=0.9598, Kappa=0.7798.

Environmental variables used: 1. LST (P1), 2. Prec (P1), 3. Prec (A2), 4. NDVI (P1) and 5. Prec (min). Please see additional file 2 for abbreviations and definitions.

Copyright: Licensed to the Malaria Atlas Project (MAP; www.map.ox.ac.uk) under a Creative Commons Attribution 3.0 License (http://creativecommons.org/).

Citation: Sinka *et al.* (2011). The dominant *Anopheles* vectors of human malaria in the Asia-Pacific region: occurrence data, distribution maps and bionomic précis. Parasites & Vectors, 4: 89.

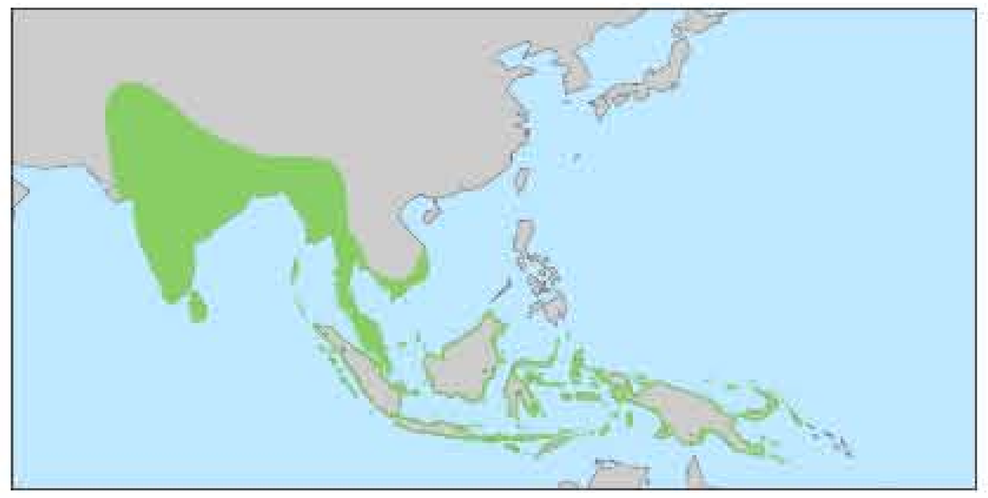

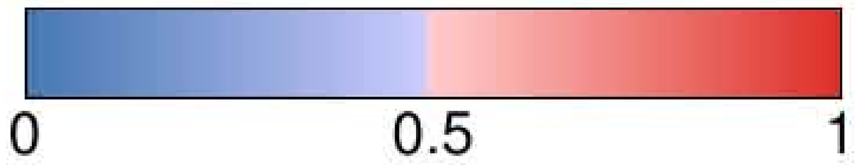

Anopheles (*Cellia*) *sundaicus* species complex

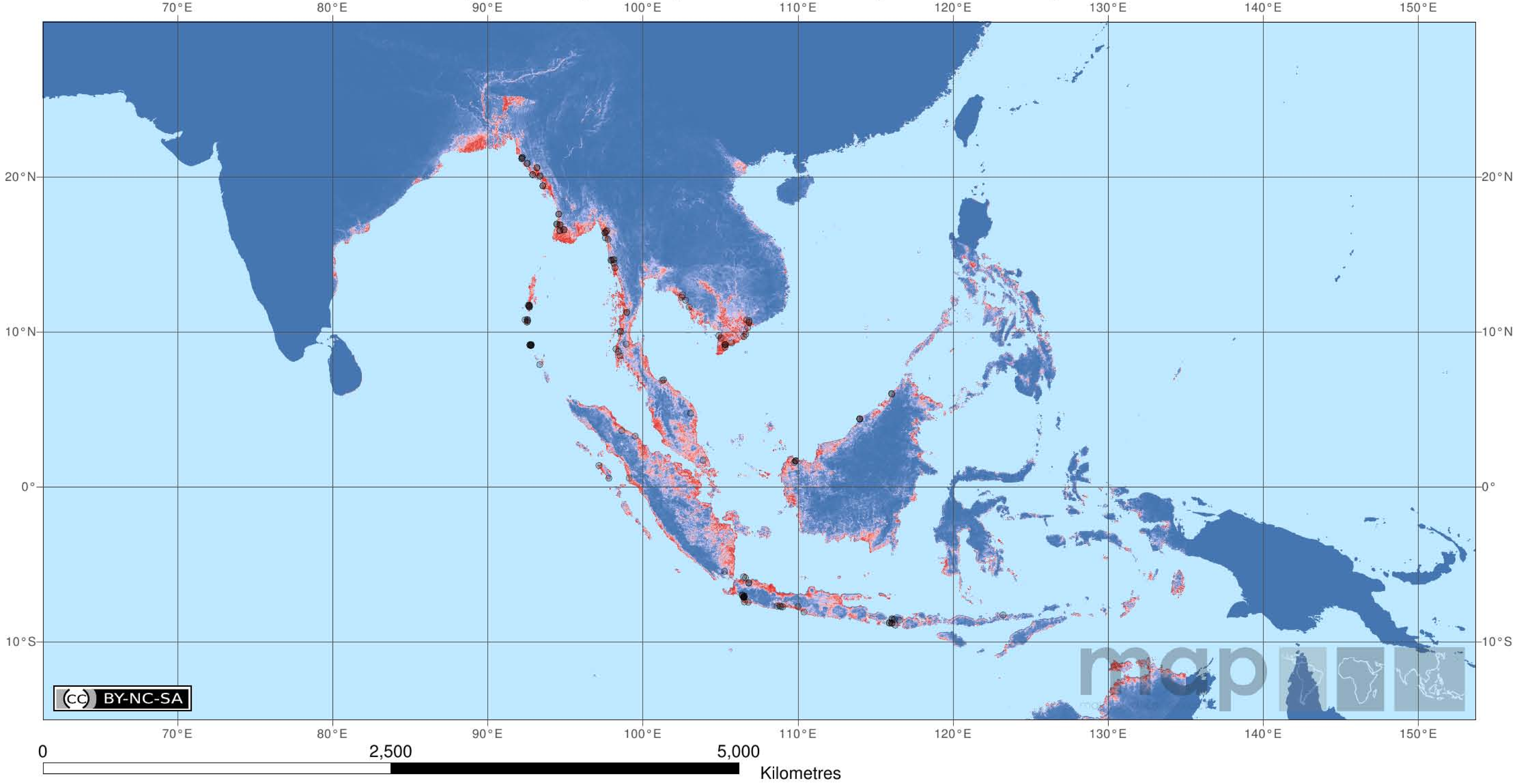

Mapping details: This map shows the predicted probability of occurrence of *An. sundaicus* in Asia-Pacific. The map was created with the Boosted Regression Trees (BRT) technique using 131 occurrence points, 500 pseudo occurrence points generated from a stratified random sample within the expert opinion range (see inset), balanced by 1,310 pseudo absence points sampled within a 1,500 km buffer outside the expert opinion range. The pseudo presence data were given half the weight of observed occurrence data. Predictions are not shown beyond the 1,500 km buffer. The black dots show 131 records of occurrence for *An. sundaicus* as detailed in Hay *et al.* [1].

Map statistics: Deviance=0.4601, Correlation=0.7433, Discrimination (AUC)=0.9353, Kappa=0.6612.

Environmental variables used: 1. DEM, 2. NDVI (P1), 3. Prec (mean), 4. Prec (max) and 5. LST (mean). Please see additional file 2 for abbreviations and definitions.

Copyright: Licensed to the Malaria Atlas Project (MAP; www.map.ox.ac.uk) under a Creative Commons Attribution 3.0 License (http://creativecommons.org/).

Citation: Sinka *et al.* (2011). The dominant *Anopheles* vectors of human malaria in the Asia-Pacific region: occurrence data, distribution maps and bionomic précis. Parasites & Vectors, 4: 89.

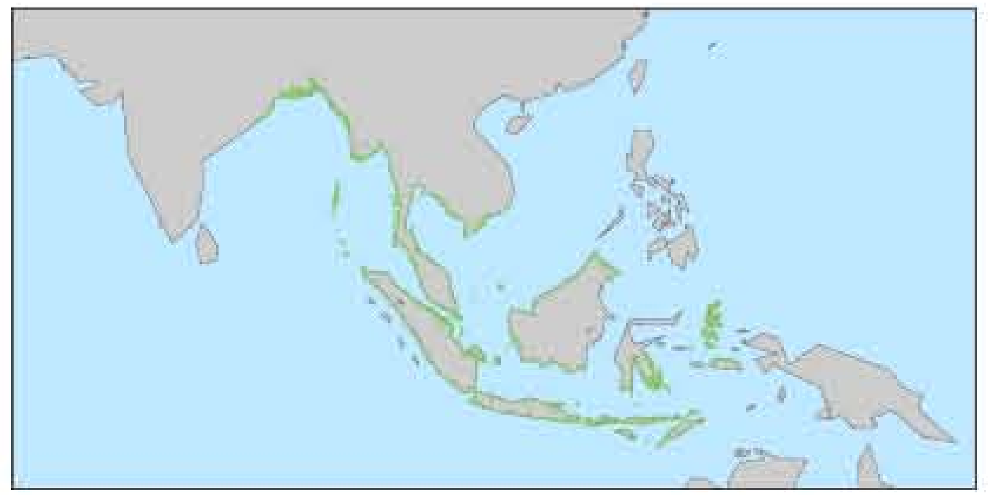

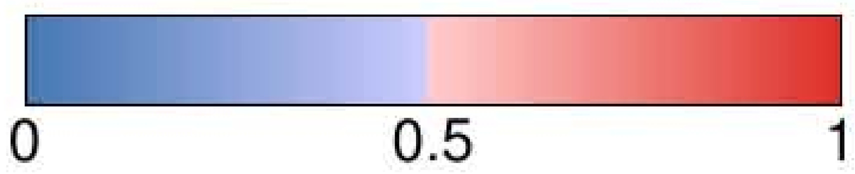

Supplement: Additional file 3 — Predictive species distribution maps for the 19 DVS of the Asian-Pacific region. [file 1756-3305-4-89-S3.PDF]
